# Highly dampened blood transcriptome response in HIV patients after respiratory infection

**DOI:** 10.1038/s41598-021-83876-9

**Published:** 2021-02-24

**Authors:** Subhashini A. Sellers, William A. Fischer, Mark T. Heise, Klaus Schughart

**Affiliations:** 1 Division of Pulmonary Diseases and Critical Care Medicine, Department of Medicine, University of North Carolina at Chapel Hill, Chapel Hill, NC USA; 2 Department of Genetics, University of North Carolina at Chapel Hill, Chapel Hill, NC USA; 3 Department of Microbiology and Immunology, University of North Carolina at Chapel Hill, Chapel Hill, NC USA; 4 Lineberger Comprehensive Cancer Center, University of North Carolina, Chapel Hill, NC USA; 5 Department of Infection Genetics, Helmholtz Centre for Infection Research, Brunswick, Germany; 6 University of Veterinary Medicine Hannover, Hannover, Germany; 7 Department of Microbiology, Immunology and Biochemistry, University of Tennessee Health Science Center, Memphis, TN USA

**Keywords:** Immunology, Infection, Infectious diseases, Innate immunity

## Abstract

Respiratory viral (RV) infections represent a major threat for human health worldwide. Persons with HIV (PWH) have a compromised immune response and are thought to be at higher risk for severe RV disease. However, very little is known about the host immune response to RV infection in PWH. Here, we investigated gene expression changes in the peripheral blood of PWH co-infected with RV. Only very few differentially expressed genes could be detected between PWH with and without RV infection, suggesting that the immune response to RV in PWH is strongly dampened. Our data provides important insights into the host response to RV infections in HIV patients.

## Introduction

Respiratory viral (RV) infections, including influenza and presently SARS-CoV-2 pose major threats to public health as they are responsible for epidemics and pandemics resulting in high morbidity and mortality worldwide^1,2^. The most severe influenza human pandemic in 1918 resulted in about 30 million fatal casualties^3^ and the current coronavirus pandemic has already resulted in over 700,000 deaths (WHO situation report 2020). Seasonal RV infections also represent a major health hazard causing morbidity, mortality, and enormous loss of the work force yearly^1,4,5^. Very little is known about the immune response in persons with HIV (PWH) after RV infection. Symptoms of RV infection appear to be similar in PWH and non-HIV patients^6^. However, in a study of 358 PWH infected with influenza virus (IV), lower respiratory tract disease incidence was 4–8 times higher than in non-HIV patients infected with IV only^7^. In addition, HIV-IV co-infected patients were more likely to have other bacterial infections^7–10^. Evidence from antiretroviral therapy (ART)-limited regimens demonstrates that HIV infection is a risk factor for higher rates of hospitalization, need for supplemental oxygen, and mechanical ventilation compared to PWH without RV infections^11,12^. Treatment with high-active antiretroviral therapy (HAART) decreased mortality after influenza infections by three to sixfold in PWH but still remained higher than in non-HIV patients^13^. Thus, PWH are thought to be at higher risk for more severe outcomes after RV infection. In a review of the literature in 2016, González Álverez et al.^13^ concluded that PWH may be at higher risk for bacterial superinfection because of their humoral and cellular immune suppression. In agreement with this, coinfection and immunosuppression were found to be a major risk factor for influenza mortality^8^. On the other hand, PWH demonstrate a diminished cytokine response, which may actually be protective against severe hyper-inflammatory responses, often observed in severe RV infections^13^. In general, evidence of the impact of HIV infection in RV severity is scarce and often contradictory because stratification of the immune status is often absent in RV-infected patients^13^.

To gain more insight into the host immune response to RV in PWH, we performed a blood transcriptome study in PWH after RV infection and aimed to identify differentially expressed genes (DEGs) compared to PWH without RV infection. We found only very few DEGs between HIV-RV co-infected and HIV-only patients. These results indicate that PWH mount a less robust immune response after infection with RV.

## Results

### Patient cohort

We collected samples from HIV-infected and HIV-RV co-infected patients which consisted of 40 HIV-only infected, and 18 HIV-RV co-infected patients (Table 1) collected between August 2015 and August 2018. In addition, we added 4 non-HIV/non-RV healthy controls to the transcriptome study for a trend analysis. Table S1 lists the clinical characteristics for each individual patient.

**Table 1:** Characteristics of cohort, stratified by infectious status.

	PWH with RV (n = 18)	PWH without RV (n = 40)	No HIV or RV (n = 4)
Male sex	9 (50%)	28 (70%)	2 (50%)
Age, median (IQR)	51 (39–60)	50 (20–72)	33 (27–40)
On ART	13 (72%)	27 (68%)	NA
CD4 count (cells/μL), median (IQR)	251 (84–589)	175 (15–586)	NA
Undetectable viral load	12 (67%)	24 (60%)	NA

*PWH* persons with HIV, *RV* respiratory virus, *ART* antiretroviral therapy.

### Identification of differentially expressed genes

**Figure 1:**
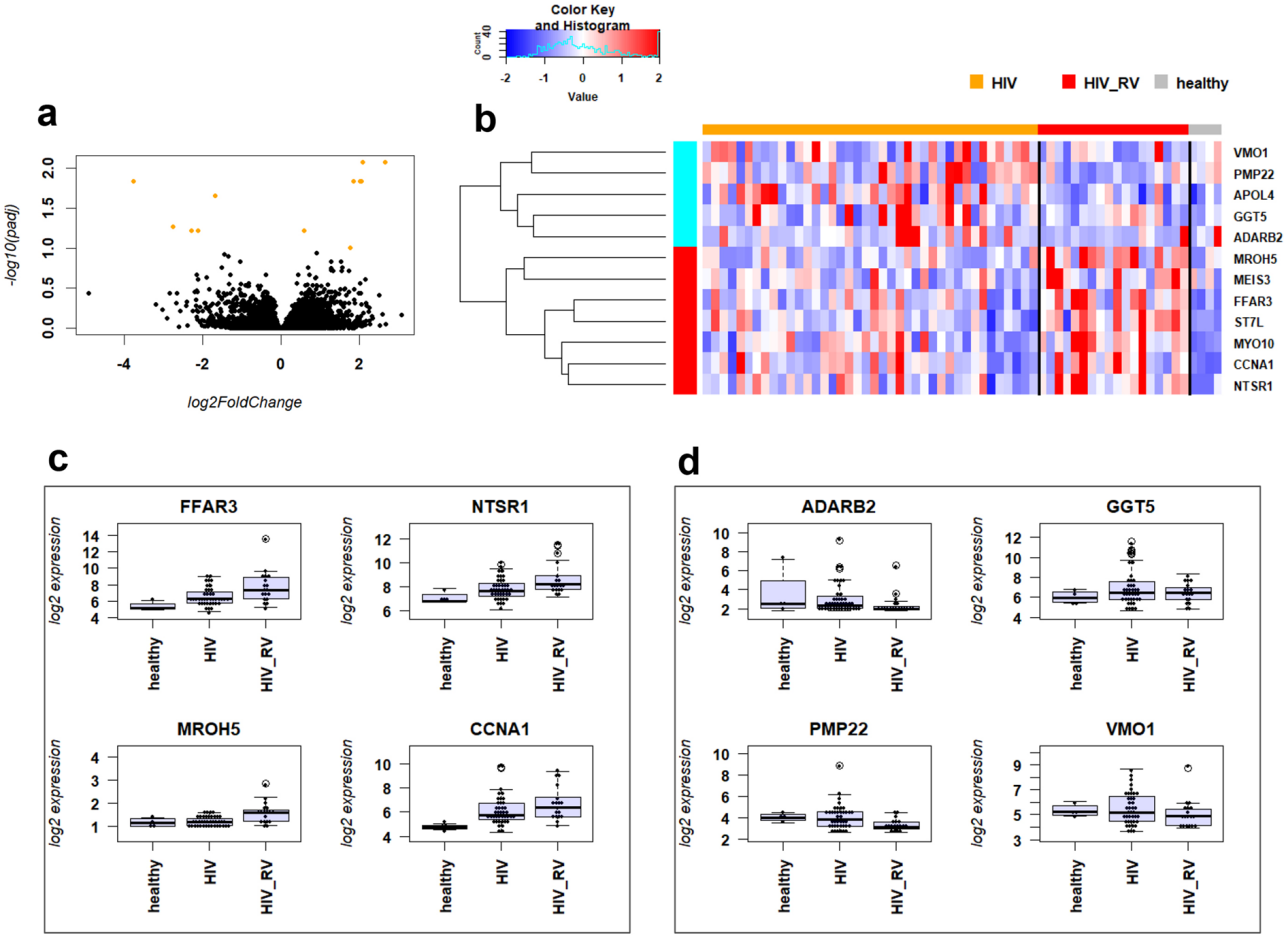
Visualization of DEGs in PWH cohort. (**a**) Volcano plot showing DEGs with thresholds of absolute fold change > 1.5 and a BH adjusted *p* value < 0.5 in yellow. (**b**) Heatmap of relative expression levels of DEGs identified between infected and non-infected PWH. Values were scaled by row. (**c**) Boxplot of individual gene expression levels as normalized log_2_ expression values for top four up-regulated DEGs. (**d**) Boxplot of individual gene expression levels as normalized log_2_ expression values for top four down-regulated DEGs. healthy: healthy controls; HIV: non-infected PWH; HIV_RV: PWH infected with respiratory virus. Gene name abbreviations: Free Fatty Acid Receptor 3 (*FFAR3*), Neurotensin Receptor 1 (*NTSR1*), Maestro Heat Like Repeat Family Member 5 (*MROH5*), Cyclin A1 (*CCNA1*), Adenosine Deaminase RNA Specific B2 (*ADARB2*), Gamma-Glutamyltransferase 5 (*GGT5*), Peripheral Myelin Protein 22 (*PMP22*), Vitelline Membrane Outer Layer 1 Homolog (*VMO1*).

We determined differentially expressed genes (DEGs) between non-infected and infected PWH using a threshold of absolute fold changes > 1.5 (log_2_ = 0.58496) and a BH multiple testing adjusted *p* value of < 0.1. We identified 12 DEGs, 7 up- and 5 down-regulated genes (Table S2). Figure 1 shows the corresponding Volcano plot (Fig. 1a), heatmap for all DEGs (Fig. 1b), and individual expression levels of the top four up- and down-regulated DEGs (Fig. 1c, d, respectively). These findings suggest that the immune response in PWH with RV infection is dampened compared to PWH baseline.

### Strongly dampened immune response in HIV compared to non-HIV patients

In our PWH cohort, we found extremely few DEGs when contrasting infected to non-infected PWH. This may be due to a biological difference or simply due to a small sample size. Therefore, we performed a power analysis to determine if the sample size in our cohort would in principle will be large enough to detect DEGs with a fold change of 1.5 and a false discovery rate of 0.1. Using the R package ssizeRNA^14^ which has been developed for power calculations of RNAseq data, we calculated a power of 0.83 to detect 5% of genes as DEGs in a two group comparison with a sample size of 18 for the smaller group. Thus, if the number of DEGs in our data set was 1000 or more, we would have detected them with an 83% chance (80% is generally used as threshold for statistical analyses). If the number of DEGs in our data set was 500 or less, the power to detect DEGs with these thresholds drops to 0.64. Thus, we conclude that the sample size in our cohort was sufficiently large for detecting many more DEGs for the thresholds chosen.

**Figure 2:**
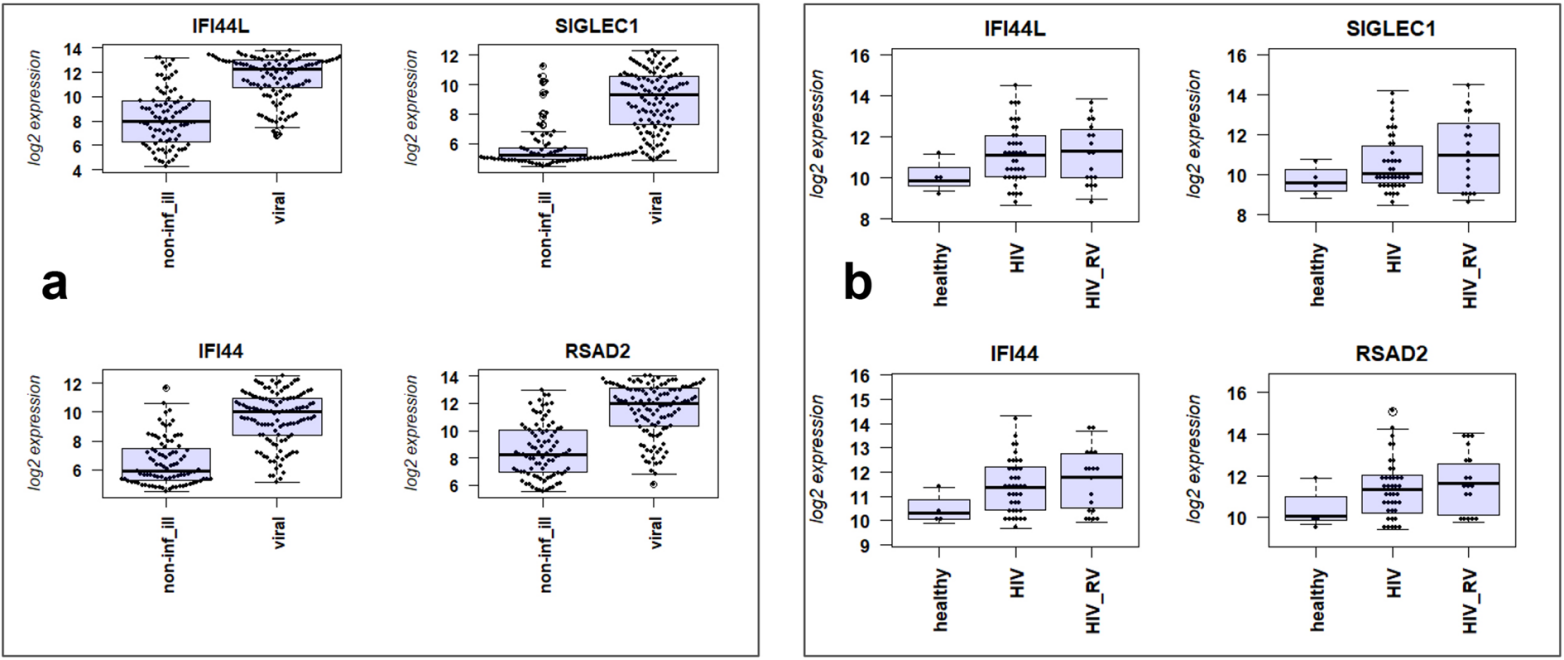
Comparison of expression levels of top regulated genes from non-HIV cohort. Boxplot of individual gene expression levels as normalized log_2_ expression values. The top four genes up-regulated in the non-HIV cohort are shown for the non HIV-cohort in (**a**) and for the PWH cohort in (**b**). All pairwise differences in (**a**) are statistically significant (*p* < 0.01); all differences in (**b**) are not significantly different. For non-HIV cohort: non-inf-ill: control group in non-HIV infected patients who had lung conditions but no infections, viral: patients infected with respiratory virus in non-HIV cohort. For PWH cohort: healthy: healthy controls; HIV: non-infected PWH; HIV_RV: PWH infected with respiratory virus. Gene name abbreviations: Interferon Induced Protein 44 Like (*IFI44L*), Sialic Acid Binding Ig Like Lectin 1 (*SIGLEC1*), Interferon Induced Protein 44 (*IFI44*), Radical S-Adenosyl Methionine Domain Containing 2 (*RSAD2*).

To further support our hypothesis, we analyzed a published data set in which patients with respiratory infections were compared to controls in non-HIV patients^15^. The published non-HIV cohort consisted of 115 RV-infected patients and 88 controls (patients with non-infectious illness). In this cohort, we detected 3833 DEGs when contrasting infected to controls, 1902 up-regulated and 1931 down-regulated genes, using a threshold of absolute fold change > 1.5 and a BH multiple testing adjusted *p* value of < 0.05 (Table S3). The expression levels of the top four genes [Interferon Induced Protein 44 Like (*IFI44L*), Sialic Acid Binding Ig Like Lectin 1 (*SIGLEC1*), Interferon Induced Protein 44 (*IFI44*), Radical S-Adenosyl Methionine Domain Containing 2 (*RSAD2*)] up-regulated in non-HIV patients are shown in Fig. 2. Most remarkably, no significant differences between non-infected and RV-infected PWH were found in our HIV-cohort (Fig. 2) for these genes.

The published data set has larger group sizes for infected and controls than our PWH cohort. Therefore, we simulated the sample sizes from our data set and randomly selected 18 subjects with RV infections and 40 controls (sample sizes in our cohort) from the published non-HIV data set of Tsalik et al.^15^. For these randomly selected samples, we calculated the number of differentially expressed genes using the same thresholds as in our HIV study. The simulation was repeated 50 times. We detected on average 3738.56 DEGs (SEM = 137.99)—many more than the 12 DEGs that were detected in our PWH cohort. These results demonstrate that it is possible to detect a large number of differentially expressed genes in patients without HIV.

Taken together, the above findings support our hypothesis that the immune response in PWH is strongly dampened during respiratory viral infection compared to non-HIV infected patients.

### Limitations

Our study has several limitations. We identified very few differentially expressed genes. This should in principle not be due to the small sample size of infected (18) versus non-infected HIV (40) patients, as we showed in our power analysis and the simulation of published data for non-HIV patients. However, it is possible that more DEGs would be detected if the sample size was increased.

The main emphasis of our study was to compare PWH with and without RV infection. Therefore, we included only a few healthy control samples from non-infected non-HIV patients. The sample size for these controlled samples was much too small to perform statistically significant contrasts, and we only used this group to describe trends. We speculate that in HIV patients, the expression of many innate immune genes was already upregulated, regardless of respiratory viral co-infection status. In order to support this speculation, it would be necessary to include a larger set of infected and non-infected samples from patients without HIV.

The sample size of PWH (18) was too small to identify significant effects of covariates, such as sex, viral load, cell counts, and anti-retroviral treatment. Nevertheless, we contrasted patients with high (> 10^2^; 6 samples) to patients with low (0; 12 samples) viral loads in the group of RV-infected PWH. We only found two DEGs (*KLHDC7B* upregulated, and *ZFP57* down-regulated). We conclude that that the sample sizes were too low for a meaningful comparison and that studies on the effect of covariates will have to be repeated with much larger sample sizes.

Our analysis was based on RNASeq as the method for transcriptome analysis. The published data set which we used for our comparison to a non-HIV cohort was obtained from a microarray study. Therefore, although very unlikely, it is possible that the observed differences in outcome are due to different technologies being used.

Lastly, as is the case for many human studies, replication in another independent cohort will be important to confirm our findings.

## Discussion

Here, we analyzed gene expression changes in PWH after respiratory infections. We identified 12 DEGs in the comparison of non-RV-infected patients to RV-infected patients. Below, we discuss the top four up-and down-regulated DEGs that were at least fourfold different as examples. However, this should not be taken to mean that others are less relevant.

The top four genes up-regulated in PWH with RV infection were *FFAR3, NTSR1, MROH5, CCNA1*. The Free Fatty Acid Receptor 3 (*FFAR3*) gene encodes a G protein-coupled receptor that is activated by short chain fatty acids (SCFAs). It plays a role in the regulation of energy homeostasis and in intestinal immunity, regulating the production of chemokines and cytokines in intestinal epithelial cells^16^. Neurotensin Receptor 1 (*NTSR1*) encodes a protein that belongs to the superfamily of G-protein coupled receptors. NTSR1 mediates the functions of neurotensin, e.g. hypotension, hyperglycemia, hypothermia, antinociception, and intestinal motility and secretion^16^. Maestro Heat Like Repeat Family Member 5 (*MROH5*) does not have any known biological functions. Cyclin A1 (*CCNA1*) encodes a protein of the cyclin family. It functions as regulator of CDK kinases characterized by a periodicity in the cell cycle. It binds to other cell cycle regulators, such as retinoblastoma (Rb) family proteins, transcription factor E2F-1, and the p21 family proteins^16^.

The top four genes down-regulated in PWH with RV infection were *ADARB2, GGT5, PMP22, VMO1*. Adenosine Deaminase RNA Specific B2 (*ADARB2*) encodes a protein that is a member of the double-stranded RNA adenosine deaminase family of RNA-editing enzymes^16^. Gamma-Glutamyltransferase 5 (*GGT5*) encodes a protein that is a member of the gamma-glutamyl transpeptidase gene family. It converts leukotriene C4 to leukotriene D4^16^. Peripheral Myelin Protein 22 (*PMP22*) encodes an integral membrane protein that is a major component of myelin in the peripheral nervous system. Mutations in this gene are associated with various neurological diseases^16^. Vitelline Membrane Outer Layer 1 Homolog (*VMO1*) has no known biological function^16^.

In conclusion, there was no obvious shared biological function for the identified DEGs. Also, with the exception of *FFAR3*, none of the genes has a known function in host immunity or pathogen defense. Performing a functional analysis (like KEGG, Reactome or GO) was not appropriate, since the number of DEGs was so small.

In total, surprisingly few DEGs were identified in our PWH cohort compared to what would be expected in a data set with this sample size, based on our power analysis. In addition, we simulated the sample sizes in a published data set from non-HIV patients, and this analysis indicated that a much larger number of DEGs would have been observed in a similarly powered cohort of non-HIV patients. Furthermore, the most strongly regulated genes in this non-HIV cohort did not show any significant differences in our PWH cohort. Therefore, we conclude from our analyses of gene expression changes in the peripheral blood that in PWH, inflammatory responses in the blood were not activated by respiratory viral infection in PWH to the same extent that they are in non-HIV patients, and the host immune response in PWH patients to RV infections is strongly dampened.

Although we could not confirm all findings that were cited in a review by González Álvarez et al.^13^, our observations are in agreement with the general conclusions from their studies, namely that the host response to RV infection is very different and compromised in PWH compared to patients without HIV. This finding may underlie the observation that PWH are at higher risk for severe influenza disease.

González Álvarez et al.^13^ also reported that CXCL10 was consistently increased in PWH. In our dataset, we actually observed a lower level of *CXCL10* expression in HIV-RV co-infected versus PWH without RV. However, this difference was not significant. It should be noted that we analyzed the gene expression changes and not protein levels in the blood. CXCL10 protein is also secreted into the blood stream from lung tissues after IV infection. The *CXCL10* expression levels were, however, strongly and significantly increased in the non-HIV cohort after RV infection (Table S3).

Previous work described a weak B-cell response to influenza vaccination in PWH^17^. However, we could not detect a significant down-regulation of B cell markers (*CD79A, MS4A1, LINC00926, CD79B, TCL1A, HLA-DQA1, VPREB3, HLA-DQB1, CD74, HLA-DRA*) in our patient cohort. It should be noted that we studied transcriptome responses of all cells in peripheral blood and the B-cell signature may not be apparent because it increases at later stages in the course of an infection. It is expected to be absent during the early stages which we studied, when people present with symptoms.

In conclusion, PWH mount a minimal immune response in the peripheral blood after infection with respiratory viruses compared to patients without HIV. Our results represent insights to better understand the host response to RV infections in PWH with a compromised immune response at the molecular level.

## Methods

### Ethics statement

All methods were performed in accordance with the relevant guidelines and regulations. Informed consent was obtained for study participation and/or their legal guardians. The protocols for this study have been approved by the IRB of the University of North Carolina at Chapel Hill, IRB Numbers: 13-2140 and 15-3179.

### Sample collection and preparation of RNA

Peripheral blood in the HIV cohort was collected into Tempus Blood RNA Tubes (Applied Biosystems) and RNA isolated using the MagMAX Sample Extraction kit (Life Technologies, Germany) as described by the manufacturer. The non-HIV cohort sampling of total blood and RNA preparation (PAXgene Blood RNA Kit; Qiagen) has been described in detail previously^15^.

### Sequencing

Quality and integrity of total RNA was controlled on the Bioanalyzer Instrument (Agilent Technologies; Waldbronn, Germany). For the library construction, globin mRNA was depleted from total RNA using the GLOBINclear Kit, human (ThermoFisher, Invitrogen). Subsequently, a strand-specific RNA sequencing library was generated using NEBNext Ultra II Directional RNA Library Prep Kit (New England Biolabs) according to manufacturer’s protocols. The libraries were sequenced on Illumina HiSeq 4000 using HiSeq 3000/4000 SBS Kit (300 cycles) with an average of 51 M reads per sample.

### Data processing and analysis

Reads were quality checked with package FastQC^18^ (version 0.11.4), then trimmed using Trimgalore (version 0.4.4^19^) with default settings. Trimmed reads were mapped to human genome annotation hg38 (ENSMBL hg38 release 91) using STAR^20^ (version 2.5.2b) with default settings. Number of raw reads per sample: mean of 50,875,283), trimmed reads per sample: mean of 50,817,247), uniquely mapped reads per sample: mean of 48,400,288), and percentage of mapped reads per sample: mean of 95%). Mapped reads were counted using RsubRead^21^ (version 1.32.4). Raw counts were normalized and log_2_ transformed using DESeq2^22^ (version 1.16.1). Differentially expressed genes were identified using the function DESeqDataSetFromMatrix and DESeq in the package DESeq2. Subsequently, analysis and visualization of expression data was performed using the R software package^23^ (version 3.4.0) on the log_2_ normalized expression levels generated by the function rlogTransformation in the package DESeq2. The R package ssizeRNA (version 1.3.2)^14^ was used for a power calculation to detect DEGs in our cohort with the following parameter settings: FDR = 0.1; sample size = 18; dispersion = 0.1, number of genes = 20,000; number of simulations = 10, up-regulated genes = 50%; mean counts for control group calculated from our data = 2079). For beeswarm graphs of expression levels, package beeswarm^24^ (version 0.2.3.) was used. Published data set for non-HIV patients: Processed array data were downloaded from the GEO expression database (http://www.ncbi.nlm.nih.gov/geo/, ID: GSE63990) and analyzed using the R software^25^. Downloaded data was log_2_-transformed and quantile normalized. Only data from RV-infected patients and controls (non-infection-illness) was used in this study; patients with bacterial infections were not included. For identification of differentially expressed genes (DEGs), the LIMMA package^26^ (version 3.32.4) using BH correction for multiple testing^27^. For the simulation studies, the corresponding number of samples (18 for controls and 40 for infected) were randomly selected from the data set and DEGs were identified using a threshold of absolute fold changes > 1.5 (log2 = 0.58496) and a BH multiple testing adjusted *p* value of < 0.1 which is identical to the method of identifying DEGs in our PWH cohort. The simulations were repeated 50 times and the average number of DEGs and the corresponding standard error of means (SEM) was determined.

## Supplementary Information

Supplementary Information.

## Data Availability

Raw and processed data are available at GEO (https://www.ncbi.nlm.nih.gov/geo/); ID: GSE155352 for our HIV-cohort. Supplements: Table S1: Patient characteristics for individual samples, Table S2: DEGs for PWH infected with RV versus PWH without RV infection, Table S3: DEGs of contrast between healthy controls and patients infected with RV in the non-HIV cohort (data from^15^).
