# Author Correction: Highly dampened blood transcriptome response in HIV patients after respiratory infection

**DOI:** 10.1038/s41598-021-90793-4

**Published:** 2021-05-31

**Authors:** Subhashini A. Sellers, William A. Fischer, Mark T. Heise, Klaus Schughart

**Affiliations:** 1 Division of Pulmonary Diseases and Critical Care Medicine, Department of Medicine, University of North Carolina at Chapel Hill, Chapel Hill, NC USA; 2 Department of Genetics, University of North Carolina at Chapel Hill, Chapel Hill, NC USA; 3 Department of Microbiology and Immunology, University of North Carolina At Chapel Hill, Chapel Hill, NC USA; 4 Lineberger Comprehensive Cancer Center, University of North Carolina, Chapel Hill, NC USA; 5 Department of Infection Genetics, Helmholtz Centre for Infection Research, Brunswick, Germany; 6 University of Veterinary Medicine Hannover, Hannover, Germany; 7 Department of Microbiology, Immunology and Biochemistry, University of Tennessee Health Science Center, Memphis, TN USA

Correction to: *Scientific Reports* 10.1038/s41598-021-83876-9, published online 24 February 2021

The original version of this Article contained an error in the title of the paper, where the term “respiratory infection” was incorrectly given as “influenza infection”.

In addition, in the Discussion section,

“Here, we analyzed gene expression changes in PWH after influenza infections.”

now reads:

“Here, we analyzed gene expression changes in PWH after respiratory infections.”

The original Article and accompanying Supplementary Information file have been corrected.

