# Supplementary material for: Highly dampened blood transcriptome response in HIV patients after respiratory infection
Source: Sci Rep. 2021 Feb 24;11:4465. doi: 10.1038/s41598-021-83876-9 (PMC7904929; doi:10.1038/s41598-021-83876-9)
Supplement: Supplementary file 1 — Supplementary Information. [file 41598_2021_83876_MOESM1_ESM.pdf]

Supplements

Highly dampened blood transcriptome response in HIV patients after respiratory infection

Authors

Subhashini A. Sellers[1], William A. Fischer II[1], Mark T. Heise[2,3,4], and Klaus Schughart[5,6,7#]

KS: ORCID: 0000-0002-6824-7523

Author affiliations

[1]Division of Pulmonary Diseases and Critical Care Medicine, Department of Medicine, University of North Carolina at Chapel Hill, Chapel Hill, NC, USA

[2]Department of Genetics, University of North Carolina at Chapel Hill, Chapel Hill, NC, USA

[3]Department of Microbiology and Immunology, University of North Carolina at Chapel Hill, Chapel Hill, NC, USA

[4]Lineberger Comprehensive Cancer Center, University of North Carolina, Chapel Hill, NC, USA

[5]Department of Infection Genetics, Helmholtz Centre for Infection Research, Braunschweig, Germany

[6]University of Veterinary Medicine Hannover, Hannover, Germany

[7]Department of Microbiology, Immunology and Biochemistry, University of Tennessee Health Science Center, Memphis, Tennessee, USA

[#]corresponding author: KS (e-mail: kls@helmholtz-hzi.de)

Supplement Table S1. Description of patient characteristics.

sample_ID	status	Age	sex	HIV_titer
INFG_010	HIV	56	female	0
INFG_011	HIV	43	female	1586
INFG_012	HIV	54	male	0
INFG_013	HIV	48	male	0
INFG_014	HIV_vir	19	female	0
INFG_015	HIV	44	male	0
INFG_019	HIV	62	female	0
INFG_020	HIV	60	male	0
INFG_021	HIV_vir	50	male	0
INFG_022	HIV_vir	46	male	24400
INFG_023	HIV_vir	39	female	5614
INFG_024	HIV	55	male	656766
INFG_026	HIV	60	male	0
INFG_027	HIV	56	male	854
INFG_028	HIV	49	male	7917
INFG_029	HIV_vir	60	male	0
INFG_030	HIV_vir	67	male	0
INFG_031	HIV_vir	65	female	0
INFG_032	HIV	65	male	0
INFG_033	HIV	51	female	81129
INFG_035	HIV	20	female	55322
INFG_036	HIV_vir	38	female	0
INFG_037	HIV	62	male	1949
INFG_042	HIV_vir	55	male	0
INFG_043	HIV	72	male	57875
INFG_044	HIV	66	male	0
INFG_045	HIV	43	male	24923
INFG_046	HIV_vir	27	male	190507
INFG_048	HIV	21	female	0
INFG_050	HIV	57	male	0
INFG_051	HIV_vir	48	female	26698
INFG_052	HIV_vir	61	female	0
INFG_053	HIV_vir	51	male	0
INFG_055	HIV	65	male	3825
INFG_056	HIV	70	male	0
INFG_059	HIV	21	female	0
INFG_060	HIV	46	male	0
INFG_061	HIV	46	male	0
INFG_062	HIV_vir	67	female	0
INFG_063	HIV	46	male	0
INFG_064	HIV	56	female	0
INFG_065	HIV	21	female	38025
INFG_066	HIV_vir	21	female	138
INFG_067	HIV	35	female	73325
INFG_068	HIV	30	male	471237
INFG_070	HIV	53	male	568
INFG_072	HIV_vir	60	male	0
INFG_073	HIV	53	female	0

INFG_075	HIV	52	male	29917
INFG_076	HIV	59	male	827
INFG_078	HIV_vir	44	female	933923
INFG_080	HIV_vir	54	male	0
INFG_HIRE_020	HIV	30	male	0
INFG_HIRE_021	hlty_ctrl	24	male	0
INFG_HIRE_023	HIV	31	male	0
INFG_HIRE_024	HIV	39	female	0
INFG_HIRE_026	HIV	27	male	0
INFG_HIRE_027	hlty_ctrl	44	female	0
INFG_HIRE_028	hlty_ctrl	35	female	0
INFG_HIRE_029	HIV	39	male	0
INFG_HIRE_031	hlty_ctrl	30	male	0
INFG_HIRE_032	HIV	27	male	0

**Supplement Table S2.** List of DEGs from contrasting RV-infected PWH to PWH			
ENSEMBL_gene_ID	gene_symbol	log2FoldChange	padj
ENSG00000185736	ADARB2	-3,752476172	0,014564306
ENSG00000099998	GGT5	-2,753010089	0,053856311
ENSG00000109099	PMP22	-2,2845005	0,060211889
ENSG00000182853	VMO1	-2,108325097	0,060211889
ENSG00000100336	APOL4	-1,687021617	0,022202112
ENSG00000007341	ST7L	0,594157962	0,060211889
ENSG00000105419	MEIS3	1,760103003	0,099081947
ENSG00000145555	MYO10	1,847511673	0,014564306
ENSG00000133101	CCNA1	2,009271176	0,014564306
ENSG00000226807	MROH5	2,040943357	0,014564306
ENSG00000101188	NTSR1	2,083877569	0,008424109
ENSG00000185897	FFAR3	2,65239769	0,008424109

Supplement Table S3. List of differentially expressed genes after contrasting samples from patients infected with respiratory virus versus controls.

ID	Gene.Symbol	logFC	adj.P.Val
204439_at	IFI44L	3,29363645	2,05E-22
219519_s_at	SIGLEC1	3,22380541	1,10E-25
214059_at	IFI44	3,06208846	3,10E-25
213797_at	RSAD2	2,96839346	1,56E-20
203153_at	IFIT1	2,9475513	2,50E-22
205569_at	LAMP3	2,67868064	2,17E-19
218986_s_at	DDX60	2,5237856	2,76E-26
200986_at	SERPING1	2,50423835	9,07E-21
205552_s_at	OAS1	2,39354909	1,77E-26
214453_s_at	IFI44	2,39187126	1,64E-17
218400_at	OAS3	2,34566369	3,82E-22
202270_at	GBP1	2,33108301	4,33E-24
219863_at	HERC5	2,23277384	2,16E-20
219352_at	HERC6	2,22126204	8,21E-28
204972_at	OAS2	2,20319903	9,34E-24
202086_at	MX1	2,17656979	7,98E-23
222154_s_at	SPATS2L	2,16687941	2,59E-18
202145_at	LY6E	2,16092504	1,69E-23
38241_at	BTN3A3	2,05213475	7,44E-34
205483_s_at	ISG15	2,03796783	5,29E-17
219211_at	USP18	2,00070213	8,06E-19
202869_at	OAS1	1,94416819	1,04E-17
210705_s_at	TRIM5	1,93529634	2,74E-30
204747_at	IFIT3	1,93376782	4,34E-21
216383_at	RPL18A /// RPL18AP3	1,92625434	4,96E-24
202411_at	IFI27	1,91040737	2,25E-06
205660_at	OASL	1,90650847	2,27E-20
219684_at	RTP4	1,8519242	2,45E-20
220018_at	CBLL1	1,83047761	4,26E-28
209328_x_at	HIGD2A /// LOC100506614	1,81100744	1,18E-23
210042_s_at	CTSZ	1,80528092	2,49E-24
202430_s_at	PLSCR1	1,79992941	5,45E-17
212681_at	EPB41L3	1,79859183	4,38E-26
206995_x_at	SCARF1	1,75967021	1,39E-26
202269_x_at	GBP1	1,752387	1,04E-19
218943_s_at	DDX58	1,72014565	4,77E-19
206632_s_at	APOBEC3B	1,70888553	1,01E-10
210657_s_at	Sep 04	1,69968883	1,26E-14
216191_s_at	TRDV3	1,69894225	5,48E-18
210607_at	FLT3LG	1,69371717	6,49E-23
218543_s_at	PARP12	1,68869825	6,89E-25
212224_at	ALDH1A1	1,68766048	6,53E-23
207777_s_at	SP140	1,67761541	1,13E-25
219209_at	IFIH1	1,67319178	2,78E-16
219777_at	GIMAP6	1,67314051	6,94E-30
210797_s_at	OASL	1,67001861	7,39E-20
217933_s_at	LAP3	1,65836781	1,50E-20

218152_at	HMG20A	1,65414987	2,26E-26
202901_x_at	CTSS	1,65271992	1,12E-25
204224_s_at	GCH1	1,65035072	3,34E-19
204821_at	BTN3A3	1,64999391	4,68E-32
211367_s_at	CASP1	1,62877786	1,26E-22
202375_at	SEC24D	1,61562088	2,47E-24
37943_at	ZFYVE26	1,60622649	1,37E-31
212845_at	SAMD4A	1,59309274	4,88E-23
202672_s_at	ATF3	1,58766432	7,62E-17
200923_at	LGALS3BP	1,57383309	5,93E-18
222279_at	LOC285830	1,56442029	4,36E-28
206133_at	XAF1	1,54783861	4,94E-17
221865_at	C9orf91	1,54650205	2,57E-26
214835_s_at	SUCLG2	1,54495505	6,64E-26
209619_at	CD74	1,5433597	4,48E-26
215718_s_at	PHF3	1,53713727	2,37E-24
203148_s_at	TRIM14	1,53349258	8,94E-31
210770_s_at	CACNA1A	1,52924653	3,29E-25
201043_s_at	ANP32A	1,51585497	4,97E-23
207425_s_at	Sep 09	1,51238611	1,19E-24
203690_at	TUBGCP3	1,49040721	1,90E-19
221090_s_at	OGFOD1	1,49003512	7,17E-30
204142_at	ENOSF1	1,45565005	3,70E-24
205099_s_at	CCR1	1,4543577	5,33E-24
219192_at	UBAP2	1,45168956	5,93E-25
221234_s_at	BACH2	1,44025113	7,14E-18
217412_at	TRD@	1,42806298	2,59E-23
205269_at	LCP2	1,42173176	6,81E-29
210873_x_at	APOBEC3A	1,42131981	2,75E-26
209417_s_at	IFI35	1,41729933	9,18E-20
203932_at	HLA-DMB	1,41586674	3,09E-23
AFFX-HUMRGE/M10098_3_at		1,40594831	9,64E-20
219716_at	APOL6	1,40437231	9,39E-20
220146_at	TLR7	1,40376098	7,56E-21
219256_s_at	SH3TC1	1,40196516	2,68E-23
201139_s_at	SSB	1,38838404	2,22E-25
201649_at	UBE2L6	1,38051516	7,96E-24
221558_s_at	LEF1	1,3783929	2,75E-15
212709_at	NUP160	1,37170959	1,66E-23
205992_s_at	IL15	1,36640402	1,05E-19
209846_s_at	BTN3A2	1,36584187	8,76E-28
211864_s_at	MYOF	1,36533864	8,49E-18
205801_s_at	RASGRP3	1,36480893	1,81E-25
214049_x_at	CD7	1,36436307	1,35E-19
210458_s_at	TANK	1,35817527	1,19E-17
219364_at	DHX58	1,34920829	3,35E-20
204700_x_at	C1orf107	1,34756116	1,88E-27
200685_at	SRSF11	1,3434676	1,51E-16
205887_x_at	MSH3	1,34131213	3,18E-25
204129_at	BCL9	1,33325692	2,07E-23

202663_at	WIPF1	1,33260924	1,56E-22
214678_x_at	ZFX	1,32969331	4,33E-23
214590_s_at	UBE2D1	1,32893159	1,39E-14
201762_s_at	PSME2	1,32750901	1,03E-31
201880_at	ARIH1	1,32668495	1,24E-23
220059_at	STAP1	1,3233859	2,28E-24
201339_s_at	SCP2	1,31976964	5,44E-19
209861_s_at	METAP2	1,31767896	1,27E-22
202820_at	AHR	1,31545974	2,25E-17
204791_at	NR2C1	1,3129078	8,64E-22
206914_at	CRTAM	1,31143569	4,07E-25
221531_at	WDR61	1,30271695	1,10E-23
208436_s_at	IRF7	1,29886379	2,80E-16
216859_x_at		1,29882707	2,84E-25
219433_at	BCOR	1,29820493	1,97E-24
209773_s_at	RRM2	1,29487616	1,67E-11
215731_s_at	MPHOSPH9	1,29338211	4,59E-22
209457_at	DUSP5	1,28788355	2,37E-22
221893_s_at	ADCK2	1,28578064	4,67E-28
218256_s_at	NUP54	1,28472789	3,59E-20
203533_s_at	CUL5	1,27975109	4,22E-21
213684_s_at	PDLIM5	1,27803607	2,63E-26
37152_at	PPARD	1,27356688	3,69E-24
219691_at	SAMD9	1,27026288	3,90E-14
221652_s_at	C12orf11	1,26824191	7,70E-23
208935_s_at	LGALS8	1,2643665	7,49E-22
209640_at	PML	1,26431033	3,28E-21
221208_s_at	C11orf61	1,26385527	4,26E-25
215524_x_at	TRAC /// TRAJ17 /// TRAV20	1,26145339	9,68E-23
203646_at	FDX1	1,25788872	4,26E-24
219037_at	RRP15	1,25723614	1,62E-27
209374_s_at	IGHM	1,25561052	2,80E-12
206503_x_at	PML	1,25332588	1,90E-24
216705_s_at	ADA	1,25213687	6,56E-28
215176_x_at	IGK@ /// IGKC	1,25034897	1,25E-09
203900_at	KIAA0467	1,24644554	7,18E-26
221680_s_at	ETV7	1,24330757	4,69E-14
212999_x_at	HLA-DQB1	1,24144815	1,14E-11
203939_at	NT5E	1,24126677	5,34E-22
218696_at	EIF2AK3	1,24079163	2,28E-19
219700_at	PLXDC1	1,24045475	1,01E-21
217346_at	LOC100293160 /// PPIA	1,23926708	1,79E-23
200887_s_at	STAT1	1,23907908	2,89E-20
213348_at	CDKN1C	1,23707801	7,71E-11
208087_s_at	ZBP1	1,23545925	1,09E-16
52731_at	AMBRA1	1,23030816	3,29E-30
212657_s_at	IL1RN	1,2282637	2,68E-23
221634_at	RPL23AP7	1,22744064	2,33E-21
207655_s_at	BLNK	1,22684759	4,82E-16
218050_at	UFM1	1,2263196	7,85E-23

44673_at	SIGLEC1	1,22531951	3,74E-13
210970_s_at	IBTK	1,22093142	5,26E-19
210317_s_at	YWHAE	1,22056765	1,26E-19
212459_x_at	SUCLG2	1,21613136	1,70E-22
204533_at	CXCL10	1,21359646	3,30E-11
200733_s_at	PTP4A1	1,2132503	5,58E-14
211478_s_at	DPP4	1,2120398	8,80E-19
222011_s_at	TCP1	1,21167139	1,06E-25
203347_s_at	MTF2	1,21074559	7,78E-20
218172_s_at	DERL1	1,20931762	4,42E-21
203236_s_at	LGALS9	1,20549584	3,57E-25
204544_at	HPS5	1,20296946	6,89E-25
209551_at	YIPF4	1,20051021	3,76E-16
210691_s_at	CACYBP	1,1968147	1,96E-24
201689_s_at	TPD52	1,19641967	2,33E-18
213606_s_at	ARHGDIA	1,19359981	3,87E-15
209717_at	EVI5	1,19348359	4,16E-18
202043_s_at	SMS	1,19266906	2,33E-23
204276_at	TK2	1,1903517	7,33E-25
212247_at	NUP205	1,18917822	5,64E-30
209593_s_at	TOR1B	1,18812016	5,00E-18
41577_at	PPP1R16B	1,1876678	3,65E-21
217800_s_at	NDFIP1	1,18725182	1,16E-17
201209_at	HDAC1	1,1869975	2,43E-24
217371_s_at	IL15	1,18526684	3,75E-25
217838_s_at	EVL	1,18512767	5,92E-22
203642_s_at	COBLL1	1,18480473	1,19E-22
207753_at	ZNF304	1,18293206	7,45E-24
214881_s_at	UBTF	1,18065131	9,89E-22
220704_at	IKZF1	1,17886737	5,05E-27
213182_x_at	CDKN1C	1,17730893	8,82E-11
214705_at	INADL	1,1767904	1,88E-19
216177_at		1,17674035	2,34E-24
203177_x_at	TFAM	1,17532546	1,96E-15
214214_s_at	C1QBP	1,17434451	9,99E-24
201202_at	PCNA	1,17413126	6,40E-24
221782_at	DNAJC10	1,17353023	2,92E-18
212526_at	SPG20	1,1731218	2,52E-21
209205_s_at	LMO4	1,16940259	1,93E-29
206545_at	CD28	1,16623577	2,32E-15
214182_at		1,16288518	5,29E-22
218452_at	SMARCAL1	1,16119547	5,35E-27
209684_at	RIN2	1,15671682	4,33E-11
211013_x_at	PML	1,15593064	3,08E-23
203358_s_at	EZH2	1,15327595	8,62E-25
36920_at	MTM1	1,15289949	1,27E-19
206724_at	CBX4	1,15262739	6,49E-19
222140_s_at	GPR89A /// GPR89B /// GPR89C	1,1494188	6,46E-25
208863_s_at	SRSF1	1,1488671	1,43E-20
212780_at	SOS1	1,14610757	3,25E-23

202666_s_at	ACTL6A	1,14247959	1,85E-23
207167_at	CD101	1,14237666	6,76E-18
218842_at	RPAP3	1,14195127	1,22E-19
203049_s_at	TTC37	1,14081175	3,95E-19
220175_s_at	/ CBWD3 /// CBWD5 /// CBWD6 /// CBW	1,14017672	1,39E-19
203405_at	PSMG1	1,13751746	5,02E-23
203401_at	PRPS2	1,13730686	3,96E-20
204616_at	UCHL3	1,13682108	2,96E-20
204552_at	INPP4A	1,13338664	1,01E-18
65630_at	TMEM80	1,13314142	1,97E-24
201016_at	EIF1AX	1,13080811	8,14E-18
217394_at		1,1304169	9,21E-18
203465_at	MRPL19	1,12966074	1,48E-26
213856_at	CD47	1,12863436	1,15E-25
212659_s_at	IL1RN	1,1263487	3,89E-18
221728_x_at	XIST	1,12427487	0,002123831
214061_at	WDR67	1,1216317	1,19E-24
218538_s_at	MRS2	1,12144742	1,68E-22
204639_at	ADA	1,11920863	1,49E-24
215796_at		1,11795003	9,88E-21
214218_s_at	XIST	1,11789697	0,003872489
201280_s_at	DAB2	1,11478392	9,25E-16
203595_s_at	IFIT5	1,11419243	4,97E-09
202720_at	TES	1,11354072	1,94E-29
209268_at	VPS45	1,11334913	1,87E-21
219570_at	KIF16B	1,1133374	5,33E-24
203085_s_at	TGFB1	1,11194274	3,22E-22
202026_at	SDHD	1,11139746	2,69E-19
209520_s_at	NCBP1	1,11111986	4,67E-24
213303_x_at	ZBTB7A	1,11002703	2,73E-26
221268_s_at	SGPP1	1,11002243	3,82E-17
204275_at	SOLH	1,10966967	9,59E-22
218446_s_at	FAM18B1	1,10947427	2,30E-15
220038_at	C8orf44 /// SGK3	1,10682741	1,16E-20
213109_at	TNIK	1,10630661	1,81E-25
203199_s_at	MTRR	1,10559976	1,62E-22
206637_at	P2RY14	1,10448368	3,06E-06
213246_at	C14orf109	1,10443505	1,64E-20
213048_s_at		1,10084839	7,74E-22
202824_s_at	TCEB1	1,10080479	4,35E-17
218999_at	TMEM140	1,1001398	6,46E-19
213294_at	EIF2AK2	1,09948844	2,54E-15
215921_at	NPIPL3	1,09833435	2,89E-19
64408_s_at	CALML4	1,09768903	3,28E-25
213133_s_at	GCSH /// LOC100329108	1,09651067	5,33E-24
219979_s_at	C11orf73	1,0949256	6,59E-22
213537_at	HLA-DPA1	1,09424203	2,60E-14
214850_at	LOC100170939	1,09421024	2,81E-18
211721_s_at	ZNF551	1,09373386	1,39E-23
210017_at	MALT1	1,0927178	8,68E-20

55872_at	ZNF512B	1,09027386	1,00E-19
207614_s_at	CUL1	1,0900101	8,17E-28
212966_at	HIC2	1,08712515	9,99E-24
222369_at	NAA40	1,08701091	2,10E-21
201323_at	EBNA1BP2	1,08589432	3,44E-24
201688_s_at	TPD52	1,08555494	1,01E-14
206332_s_at	IFI16	1,08404196	4,74E-25
202930_s_at	SUCLA2	1,08248934	1,34E-15
211733_x_at	SCP2	1,08198287	3,21E-19
55081_at	MICALL1	1,08126307	4,29E-27
205260_s_at	ACYP1	1,0809897	1,47E-20
218443_s_at	DAZAP1	1,08036703	5,06E-27
208810_at	DNAJB6 /// TMEM135	1,07967875	9,37E-16
210279_at	GPR18	1,07967621	2,93E-19
209841_s_at	LRRN3	1,07868217	1,71E-13
219043_s_at	LOC285359 /// PDCL3	1,07822789	3,60E-24
222146_s_at	TCF4	1,07796789	5,24E-24
214193_s_at	C1orf107	1,07690024	8,32E-27
32723_at	CSTF1	1,07667483	5,45E-25
207545_s_at	NUMB	1,07611204	1,35E-13
205254_x_at	TCF7	1,07373911	2,00E-13
209451_at	TANK	1,07284226	1,73E-11
221221_s_at	KLHL3	1,07243164	8,78E-22
212613_at	BTN3A2	1,07204233	2,27E-23
212594_at	PDCD4	1,07199395	3,60E-24
210649_s_at	ARID1A	1,07044656	2,65E-18
218570_at	KBTBD4 /// PTPMT1	1,06967352	5,18E-27
205291_at	IL2RB	1,06864168	4,07E-16
205771_s_at	AKAP7	1,06759388	2,08E-19
218147_s_at	GLT8D1	1,06744537	4,28E-20
203689_s_at	FMR1	1,06710108	5,77E-10
32541_at	PPP3CC	1,06678162	8,78E-25
214876_s_at	TUBGCP5	1,0665827	2,59E-24
213530_at	RAB3GAP1	1,0655229	2,89E-25
206553_at	OAS2	1,06504998	6,53E-14
207002_s_at	PLAGL1	1,06344191	1,11E-14
213459_at	RPL37A	1,06306967	7,86E-25
221514_at	UTP14A	1,0626834	5,50E-28
206710_s_at	EPB41L3	1,06229093	7,80E-16
36612_at	FAM168A	1,0604506	5,70E-26
217143_s_at	TRD@	1,05998574	1,76E-11
218700_s_at	RAB7L1	1,05935142	1,78E-29
214933_at	CACNA1A	1,05686869	2,63E-21
209523_at	TAF2	1,05675788	7,77E-18
211240_x_at	CTNND1	1,05618903	9,68E-23
205518_s_at	CMAH	1,055902	7,86E-14
209544_at	RIPK2	1,05587029	2,33E-26
217979_at	TSPAN13	1,05544544	7,64E-15
34031_i_at	KRIT1	1,0548295	8,58E-22
203258_at	DRAP1	1,05358794	2,88E-29

219279_at	DOCK10	1,04791236	4,65E-16
209310_s_at	CASP4	1,0475291	8,67E-17
214617_at	PRF1	1,0469927	2,01E-14
201015_s_at	JUP	1,04685151	3,45E-12
200749_at		1,04625079	3,80E-17
221965_at	MPHOSPH9	1,04604676	2,83E-19
213773_x_at	NSUN5	1,04347133	8,06E-24
206770_s_at	SLC35A3	1,04336087	2,05E-24
217502_at	IFIT2	1,04226677	4,83E-08
205027_s_at	MAP3K8	1,04154146	1,43E-11
202049_s_at	ZMYM4	1,03689868	1,66E-23
218598_at	RINT1	1,03444793	1,32E-23
218594_at	HEATR1	1,03408769	6,84E-25
209657_s_at	HSF2	1,03387374	1,63E-19
214845_s_at	CALU	1,03386627	1,37E-22
211991_s_at	HLA-DPA1	1,03265906	2,70E-18
210385_s_at	ERAP1	1,03225446	4,48E-25
218578_at	CDC73	1,03037276	2,94E-17
213027_at	TROVE2	1,02968508	1,33E-12
210001_s_at	SOCS1	1,02848292	1,33E-08
210972_x_at	TRAC /// TRAJ17 /// TRAV20	1,02841157	3,12E-12
202783_at	NNT	1,02820182	2,01E-20
218713_at	NARG2	1,02617025	1,73E-20
222204_s_at	RRN3	1,02535911	4,49E-18
204205_at	APOBEC3G	1,02491515	9,94E-27
211965_at	ZFP36L1	1,02236661	5,62E-12
218918_at	MAN1C1	1,02106946	8,28E-17
213018_at	GATAD1	1,02103859	7,50E-23
219123_at	ZNF232	1,02099095	5,79E-24
218223_s_at	PLEKHO1	1,0203856	1,33E-23
203787_at	SSBP2	1,0203112	4,16E-17
202748_at	GBP2	1,02007061	3,38E-27
218500_at	C8orf55	1,01976259	7,21E-27
208751_at	NAPA	1,01929594	1,06E-16
211953_s_at	IPO5	1,01873968	7,57E-18
218967_s_at	PTER	1,01812755	1,66E-19
209674_at	CRY1	1,01787938	8,47E-15
215332_s_at	CD8B	1,01752302	1,45E-13
222239_s_at	INTS6	1,01750928	1,76E-15
60815_at	POLR2J4	1,01684583	7,37E-22
218501_at	ARHGEF3	1,01592158	2,97E-16
203404_at	ARMCX2	1,01350349	1,88E-24
206983_at	CCR6	1,01338249	6,07E-19
204236_at	FLI1	1,01112064	5,33E-17
217299_s_at	NBN	1,01091875	2,22E-17
205885_s_at	ITGA4	1,00905663	2,14E-12
217019_at	RPS4X /// RPS4XP6	1,00878596	5,16E-24
213206_at	GOSR2	1,00844982	1,41E-27
215440_s_at	BEX4	1,00842781	2,86E-16
203991_s_at	KDM6A	1,00721191	2,39E-12

215754_at	SCARB2	1,00685629	1,11E-23
215936_s_at	KIAA1033	1,00664426	9,22E-16
209368_at	EPHX2	1,00514524	1,06E-18
203291_at	CNOT4	1,00489905	6,46E-24
200629_at	WARS	1,00344564	2,42E-14
202953_at	C1QB	1,00302256	2,12E-08
201798_s_at	MYOF	1,00297343	6,25E-11
201138_s_at	SSB	1,00283581	2,50E-21
213826_s_at		1,00282191	2,59E-18
202971_s_at	DYRK2	1,00091685	1,06E-12
214551_s_at	CD7	1,0004106	4,29E-12
203629_s_at	COG5	1,00035515	2,78E-23
219204_s_at	SRR	1,00015155	2,92E-25
219363_s_at	MTERFD1	0,99947369	1,24E-19
213158_at		0,99925651	1,60E-17
202309_at	MTHFD1	0,99598393	1,07E-24
210093_s_at	MAGOH	0,99593051	3,47E-22
220576_at	PGAP1	0,99538205	2,50E-20
209967_s_at	CREM	0,9945423	1,92E-17
217842_at	LUC7L2	0,99448249	7,52E-22
205005_s_at	NMT2	0,99444402	1,43E-12
219006_at	NDUFAF4	0,99432223	5,44E-27
222286_at	SNAPC3	0,99339858	1,21E-27
221766_s_at	FAM46A	0,99307174	1,54E-19
211798_x_at	IGLJ3	0,99305371	5,98E-11
202007_at	NID1	0,99295241	7,22E-15
219288_at	C3orf14	0,9923963	4,34E-18
211776_s_at	EPB41L3	0,99199215	1,32E-16
218989_x_at	SLC30A5	0,99198739	7,26E-14
213980_s_at	CTBP1	0,99122214	8,65E-25
222108_at	AMIGO2	0,98951387	2,37E-18
202069_s_at	IDH3A	0,98927005	9,86E-22
212168_at	RBM12	0,98870197	1,12E-18
55662_at	C10orf76	0,98836941	5,00E-24
219031_s_at	NIP7	0,98750138	5,31E-29
204857_at	MAD1L1	0,98702409	2,39E-15
209602_s_at	GATA3	0,98696711	2,21E-17
212678_at	NF1	0,98611227	7,56E-18
217403_s_at	ZNF227	0,9857202	5,45E-19
216112_at		0,98481775	8,13E-22
53720_at	C19orf66	0,98456615	1,60E-21
221645_s_at	ZNF83	0,98453592	2,25E-13
206828_at	TXK	0,98375574	3,93E-13
206760_s_at	FCER2	0,98351362	2,48E-17
222128_at	NSUN6	0,98349167	3,96E-22
210612_s_at	SYNJ2	0,9832893	2,28E-16
214751_at	ZNF468	0,9830804	3,59E-20
208938_at	PRCC	0,98302963	4,36E-25
217631_at	GTPBP4	0,98260394	2,90E-23
211715_s_at	BDH1	0,98218536	1,19E-23

219512_at	DSN1	0,9820699	1,20E-24
209412_at	TRAPPC10	0,98148867	6,56E-14
201690_s_at	TPD52	0,98016025	2,24E-12
204516_at	ATXN7	0,97858619	4,91E-27
221480_at	HNRNPD	0,978537	1,56E-23
216558_x_at	i1 /// IGHG2 /// IGHG3 /// IGHM /// IGHV	0,97767423	3,70E-21
218305_at	IPO4	0,97758122	3,23E-30
212056_at	KIAA0182	0,97645776	8,74E-21
209922_at	BRAP	0,97576542	4,54E-21
203753_at	TCF4	0,97450791	4,20E-21
210540_s_at	B4GALT4	0,97390559	9,62E-16
214949_at		0,97377173	8,91E-23
212735_at	KIAA0226	0,97347218	5,51E-20
215671_at	PDE4B	0,97327025	9,38E-20
208407_s_at	CTNND1	0,97322642	1,76E-17
222243_s_at	TOB2	0,97279287	9,58E-24
212855_at	DCUN1D4	0,97267925	5,14E-18
204025_s_at	PDCD2	0,97255272	3,30E-24
209306_s_at	SWAP70	0,97156281	1,40E-14
209308_s_at	BNIP2	0,97130694	3,73E-08
204085_s_at	CLN5	0,97030735	2,01E-17
221803_s_at	NRBF2	0,96925223	3,88E-11
221891_x_at	HSPA8	0,96868859	2,79E-27
213734_at	WSB2	0,96795088	1,73E-19
218886_at	PAK1IP1	0,96780309	3,26E-23
201140_s_at	RAB5C	0,96749588	5,09E-23
204283_at	FARS2	0,96696858	3,31E-22
209787_s_at	HMGN4	0,96565939	3,50E-23
219143_s_at	RPP25	0,96558383	1,45E-27
207713_s_at	RBCK1	0,9655696	1,46E-23
215093_at	NSDHL	0,96537877	1,79E-25
212755_at	MON2	0,96356126	3,44E-23
205241_at	SCO2	0,96354378	1,50E-12
201801_s_at	SLC29A1	0,96333314	2,92E-18
205933_at	SETBP1	0,96330085	5,08E-21
201448_at	TIA1	0,9632288	9,80E-12
212846_at	RRP1B	0,96160069	8,62E-18
219178_at	QTRTD1	0,96139244	4,85E-25
216401_x_at		0,9612833	3,46E-09
202599_s_at	NRIP1	0,96072161	6,11E-12
208813_at	GOT1	0,96043293	3,10E-25
221841_s_at	KLF4	0,95931895	9,33E-15
209625_at	PIGH	0,95884628	1,11E-25
214298_x_at	Sep 06	0,95801411	6,16E-17
216044_x_at	FAM69A	0,9576777	1,52E-17
49306_at	RASSF4	0,95747781	5,90E-19
209741_x_at	SCAPER	0,95747049	1,05E-27
203264_s_at	ARHGEF9	0,95731665	9,47E-25
AFFX-HUMISGF3A/M97935_5_at	STAT1	0,95637192	1,44E-14
202326_at	EHMT2	0,95586709	5,81E-18

200939_s_at	RERE	0,95584444	4,87E-21
218432_at	FBXO3	0,95515342	5,43E-13
218331_s_at	C10orf18	0,954969	2,23E-18
212386_at	TCF4	0,95470913	1,27E-19
209049_s_at	ZMYND8	0,95420185	1,82E-20
221540_x_at	F2H2 /// GTF2H2B /// GTF2H2C /// GTF2H	0,95364817	1,88E-15
212380_at	FTSJD2	0,95352024	3,48E-14
218247_s_at	MEX3C	0,95248722	9,72E-14
218096_at	AGPAT5	0,95236736	1,30E-21
219734_at	SIDT1	0,95172163	1,89E-18
222182_s_at	CNOT2	0,95135079	7,21E-26
208958_at	ERP44	0,95045814	3,54E-26
220266_s_at	KLF4	0,94825246	1,51E-21
218572_at	CHMP4A	0,9471562	5,56E-27
220418_at	UBASH3A	0,94672954	7,07E-13
210203_at	CNOT4	0,94645782	3,70E-24
217954_s_at	PHF3	0,94527067	2,49E-16
218647_s_at	YRDC	0,94516367	4,13E-24
200875_s_at	NOP56	0,94515596	1,45E-19
219513_s_at	SH2D3A	0,94502898	1,22E-23
204211_x_at	EIF2AK2	0,944733	1,75E-17
205292_s_at	HNRNPA2B1	0,94429143	3,21E-26
218494_s_at	SLC2A4RG	0,94382017	1,17E-15
222151_s_at	CEP63	0,94300333	4,29E-16
219276_x_at	C9orf82	0,94281363	2,57E-16
201746_at	TP53	0,94194037	1,55E-17
212070_at	GPR56	0,94162035	6,62E-11
219283_at	C1GALT1C1	0,94032981	9,74E-12
206095_s_at	SRSF10	0,94023882	5,27E-12
212074_at	SUN1	0,93926681	1,33E-13
202446_s_at	PLSCR1	0,93909003	2,09E-09
216944_s_at	ITPR1	0,93872609	6,89E-22
210802_s_at	DIMT1L	0,93829653	1,52E-21
213097_s_at	DNAJC2	0,93783958	1,84E-24
215595_x_at		0,93684382	2,84E-25
203596_s_at	IFIT5	0,93631784	1,67E-08
205312_at	SPI1	0,9362766	2,50E-16
208896_at	DDX18	0,93609587	4,68E-17
201719_s_at	EPB41L2	0,93505004	4,36E-25
209025_s_at	SYNCRIP	0,93324955	1,08E-15
202600_s_at	NRIP1	0,93311764	4,09E-14
202613_at	CTPS	0,93133761	4,07E-23
207283_at	RPL23AP32	0,93129242	6,38E-26
210076_x_at	SERBP1	0,93125289	4,63E-24
217477_at	PIP5K1B	0,9311852	5,89E-21
205076_s_at	MTMR11	0,9307712	1,69E-15
205748_s_at	RNF126	0,92973066	2,76E-18
205153_s_at	CD40	0,92934369	1,84E-22
218297_at	FAM188A	0,92850335	1,66E-18
220019_s_at	ZNF224	0,92749177	8,34E-28

215201_at	REPS1	0,92657206	8,19E-26
211569_s_at	HADH	0,92640448	6,35E-22
220482_s_at	SERGEF	0,92621147	1,97E-22
212911_at	DNAJC16	0,92490433	3,46E-20
218179_s_at	C4orf41	0,92412598	9,15E-20
214945_at	LOC100507397	0,92385584	2,17E-14
213703_at	LOC150759	0,92365667	1,25E-15
209626_s_at	OSBPL3	0,92343065	2,13E-21
214149_s_at	ATP6V0E1	0,92337389	1,13E-16
218622_at	NUP37	0,9230584	3,78E-18
221919_at	LOC100506653	0,92238102	1,30E-19
208862_s_at	CTNND1	0,92145206	2,00E-21
218244_at	NOL8	0,92135216	2,26E-20
211532_x_at	KIR2DS2	0,92011753	5,14E-12
218511_s_at	PNPO	0,91912969	9,86E-30
216288_at	CYSLTR1	0,91912848	4,61E-25
202502_at	ACADM	0,91820612	3,70E-13
221589_s_at	LOC100506517	0,91759474	4,65E-17
205547_s_at	TAGLN	0,91637087	4,31E-15
218643_s_at	CRIPT	0,91621706	3,55E-14
209431_s_at	PATZ1	0,91590936	1,30E-20
220315_at	PARP11	0,91501992	8,24E-18
210057_at	SMG1	0,91487843	6,02E-23
212825_at	PAXIP1	0,9139612	3,29E-16
204049_s_at	PHACTR2	0,91386036	2,23E-17
203023_at	NOP16	0,91376887	5,58E-26
210786_s_at	FLI1	0,91259813	4,13E-11
218322_s_at	ACSL5	0,91259451	5,46E-25
208310_s_at	C7orf28B /// CCZ1	0,91238765	8,11E-17
36564_at	RNF19B	0,91188376	2,14E-18
52164_at	C11orf24	0,91159463	5,06E-27
202307_s_at	TAP1	0,9109297	1,63E-19
213054_at	HAUS5	0,91092687	1,00E-22
201948_at	GNL2	0,9104802	5,72E-25
205565_s_at	FXN	0,9103249	6,84E-21
202438_x_at	IDS	0,90981856	3,27E-12
203481_at	FAM178A	0,90957269	4,32E-16
211996_s_at	/// LOC348162 /// LOC613037 /// LOC72	0,90800446	5,64E-17
215009_s_at	LOC100499177	0,90795647	4,46E-15
205664_at	KIN	0,90694878	3,64E-14
205426_s_at	HIP1	0,90647585	7,02E-13
202950_at	CRYZ	0,90611556	8,92E-16
216748_at	PYHIN1	0,90521388	1,25E-17
200658_s_at	PHB	0,9046079	9,56E-22
211828_s_at	TNIK	0,90428741	6,76E-18
202982_s_at	ACOT1 /// ACOT2	0,90382634	4,43E-23
222149_x_at	GOLGA8DP /// GOLGA8E /// GOLGA8G //	0,90370381	2,36E-15
205248_at	DOPEY2	0,90361286	7,14E-16
208794_s_at	SMARCA4	0,90336051	6,42E-18
201300_s_at	PRNP	0,90305376	4,76E-09

1294_at	UBA7	0,90192239	1,67E-24
204299_at	SRSF10	0,90179052	1,82E-11
205996_s_at	AK2	0,90155194	2,33E-23
204352_at	TRAF5	0,90144889	1,22E-12
202396_at	TCERG1	0,90141291	3,83E-12
208912_s_at	CNP	0,90054132	6,37E-22
221568_s_at	LIN7C	0,89954338	1,31E-19
209731_at	NTHL1	0,89917075	3,36E-23
218536_at	MRS2	0,89878805	1,59E-22
206188_at	ZNF623	0,89807501	1,02E-22
219751_at	SETD6	0,89782781	7,13E-20
203449_s_at	TERF1	0,89768846	2,12E-17
217985_s_at	BAZ1A	0,89735982	8,98E-18
208042_at	AGGF1	0,89716453	8,26E-22
201624_at	DARS	0,89687215	1,31E-13
212609_s_at	AKT3	0,89594316	3,39E-20
218491_s_at	THYN1	0,89587537	2,36E-23
201836_s_at	SUPT7L	0,8955807	3,35E-22
214129_at	PDE4DIP	0,89535104	1,26E-20
213838_at	NOL7	0,89530906	3,04E-23
203403_s_at	RNF6	0,89468588	1,33E-08
209163_at	CYB561	0,89393251	5,23E-19
210053_at	TAF5	0,89334195	6,38E-20
217027_x_at	KPNB1	0,89320494	3,11E-25
207304_at	ZNF45	0,89302022	6,85E-21
204023_at	RFC4	0,8925502	1,07E-18
211071_s_at	MLLT11	0,89224164	4,05E-19
216598_s_at	CCL2	0,89127367	5,25E-07
203471_s_at	PLEK	0,89065654	5,39E-24
208876_s_at	PAK2	0,8902952	8,88E-19
212814_at	AHCYL2	0,89025713	3,68E-25
217205_at		0,88878449	2,32E-22
219060_at	WDYHV1	0,88801608	1,99E-25
201450_s_at	TIA1	0,88735064	6,34E-14
214953_s_at	APP	0,8870683	1,75E-14
219014_at	PLAC8	0,88702023	2,21E-14
212945_s_at	MGA	0,88651097	5,29E-23
214086_s_at	PARP2	0,88636623	4,00E-19
221123_x_at	ZNF395	0,88596966	1,37E-15
204019_s_at	SH3YL1	0,88591436	1,14E-09
200692_s_at	HSPA9	0,88583593	1,48E-16
203431_s_at	ARHGAP32	0,88515242	1,07E-22
212917_x_at	RECQL	0,88485615	3,73E-18
218232_at	C1QA	0,88410981	5,48E-08
204091_at	PDE6D	0,88407484	1,09E-26
215136_s_at	EXOSC8	0,88356155	3,64E-19
209840_s_at	LRRN3	0,88351405	7,49E-08
218398_at	MRPS30	0,88320627	2,15E-22
218187_s_at	C8orf33	0,88101875	2,76E-22
201263_at	TARS	0,88056694	9,28E-17

214484_s_at	SIGMAR1	0,87893298	5,38E-23
203775_at	SLC25A13	0,87885927	1,85E-21
221740_x_at	LOC100506162	0,87823382	1,38E-18
200755_s_at	CALU	0,87799931	1,24E-20
207008_at	CXCR2	0,87779043	2,50E-18
214038_at	CCL8	0,8774427	5,17E-06
212277_at	MTMR4	0,87675828	5,98E-23
202813_at	TARBP1	0,87652292	5,68E-25
209711_at	SLC35D1	0,8763165	2,48E-15
204137_at	GPR137B	0,87608229	4,44E-15
211796_s_at	TRBC1 /// TRBC2	0,87570422	2,15E-10
219073_s_at	OSBPL10	0,87552556	2,85E-11
210251_s_at	RUFY3	0,87537168	3,74E-25
222150_s_at	PION	0,87515333	9,83E-11
213293_s_at	TRIM22	0,87457034	5,04E-13
211267_at	HESX1	0,87444087	6,69E-15
218535_s_at	RIOK2	0,87393516	2,88E-19
213000_at	MORC3	0,87390257	7,30E-09
202572_s_at	DLGAP4	0,87376857	6,37E-20
219056_at	RNASEH2B	0,8737192	7,32E-27
203352_at	ORC4	0,87176621	4,98E-16
215096_s_at	ESD	0,87133165	6,45E-24
218766_s_at	WARS2	0,87051627	6,97E-23
41329_at	SCYL3	0,87001097	3,89E-19
208653_s_at	CD164	0,86983052	5,59E-12
204449_at	PDCL	0,86906353	3,33E-22
213879_at		0,8688436	5,93E-25
209315_at	HBS1L	0,86881944	5,07E-21
218946_at	NFU1	0,86877714	3,31E-17
200927_s_at	RAB14	0,86858457	1,55E-11
218239_s_at	GTPBP4	0,86755682	1,84E-22
217257_at	SH3BP2	0,86644802	1,58E-19
207791_s_at	RAB1A	0,86593094	2,39E-10
204531_s_at	BRCA1	0,86586706	6,71E-18
211228_s_at	RAD17	0,86584941	2,40E-18
204715_at	PANX1	0,86503144	1,49E-20
211967_at	TMEM123	0,86497203	2,16E-07
209609_s_at	MRPL9	0,86481878	1,47E-18
206976_s_at	HSPH1	0,86464123	1,64E-10
205500_at	C5	0,8645826	5,27E-15
39318_at	TCL1A	0,86431287	2,36E-06
219607_s_at	MS4A4A	0,86426311	5,59E-05
211323_s_at	ITPR1	0,86404785	1,97E-18
212621_at	TMEM194A	0,86398745	2,14E-18
219529_at	CLIC3	0,86384388	1,30E-08
209871_s_at	APBA2	0,86370509	4,22E-15
209175_at	SEC23IP	0,8633885	2,42E-12
212922_s_at	SMYD2	0,86313565	5,02E-23
220289_s_at	AIM1L	0,86281326	5,65E-20
219149_x_at	DBR1	0,86244099	3,00E-23

214115_at	VAMP5	0,86180165	1,30E-23
213129_s_at	GCSH /// LOC100329108	0,86146928	1,32E-19
209994_s_at	ABCB1 /// ABCB4	0,8612113	7,41E-23
213483_at	PPWD1	0,86095841	2,39E-11
212378_at	GART	0,86076864	4,06E-25
204210_s_at	PCYT1A	0,8606096	3,34E-17
210962_s_at	AKAP9	0,86009971	4,79E-19
218073_s_at	TMEM48	0,86002287	1,32E-18
203856_at	VRK1	0,85946386	6,49E-14
202611_s_at	MED14	0,8594448	7,04E-15
203584_at	TTC35	0,85939264	2,38E-10
218932_at	ZNHIT6	0,85919999	1,42E-15
201634_s_at	CYB5B	0,85900146	1,19E-26
219895_at	FAM70A	0,85737692	7,40E-12
218502_s_at	TRPS1	0,85728127	6,48E-11
203335_at	PHYH	0,85719428	1,13E-20
205089_at	ZNF7	0,85691249	1,06E-20
208195_at	TTN	0,85674459	5,28E-22
211368_s_at	CASP1	0,85670119	4,70E-18
212058_at	SR140	0,85616058	2,80E-12
209599_s_at	PRUNE	0,85587704	2,69E-21
205098_at	CCR1	0,85542924	2,08E-13
218565_at	C9orf114	0,85495743	1,02E-24
213064_at	ZC3H14	0,85461454	2,20E-18
205218_at	POLR3F	0,85384764	4,48E-22
201030_x_at	LDHB	0,85351083	1,65E-11
209092_s_at	GLOD4	0,85332317	8,85E-16
218516_s_at	IMPAD1	0,85313625	7,63E-21
211044_at	TRIM14	0,85310371	1,02E-14
203029_s_at	PTPRN2	0,85299192	1,35E-12
203582_s_at	RAB4A	0,8528111	2,75E-14
221987_s_at	TSR1	0,85273086	7,22E-24
209670_at	TRAC	0,85247447	7,14E-10
209139_s_at	PRKRA	0,8514501	9,69E-18
204675_at	SRD5A1	0,85133197	8,33E-19
218236_s_at	PRKD3	0,85106864	1,15E-11
210944_s_at	CAPN3	0,85065072	9,70E-16
213706_at	GPD1	0,85027577	1,07E-16
51146_at	PIGV	0,84999383	7,70E-27
212038_s_at	VDAC1	0,84975615	6,06E-21
200628_s_at	WARS	0,84948732	1,74E-11
210465_s_at	SNAPC3	0,8493144	1,96E-25
219266_at	ZNF350	0,84921172	6,78E-21
203988_s_at	FUT8	0,84873838	7,94E-19
200965_s_at	ABLIM1	0,84860259	1,11E-09
200956_s_at	SSRP1	0,84735385	1,77E-18
204190_at	USPL1	0,84733372	2,62E-21
218936_s_at	CCDC59	0,84687399	3,05E-17
219553_at	NME7	0,84623709	2,10E-28
220642_x_at	GPR89A /// GPR89B /// GPR89C	0,84620561	1,98E-22

204820_s_at	BTN3A2 /// BTN3A3	0,84584721	1,97E-22
203100_s_at	CDYL	0,84576721	1,34E-19
202414_at	ERCC5	0,84520197	1,26E-19
219817_at	C12orf47	0,84515961	3,75E-25
211464_x_at	CASP6	0,84484206	2,66E-23
218085_at	CHMP5	0,84393818	1,07E-06
218682_s_at	SLC4A1AP	0,84389574	1,09E-17
219405_at	TRIM68	0,84325544	1,87E-25
209498_at	CEACAM1	0,84308763	2,60E-05
215922_at	REPS1	0,84298993	7,06E-27
201321_s_at	SMARCC2	0,8426502	1,28E-18
207338_s_at	ZNF200	0,84227991	6,05E-12
214255_at	ATP10A	0,84191577	2,54E-22
211990_at	HLA-DPA1	0,84189777	1,71E-11
211747_s_at	LSM5	0,84129054	1,87E-12
214683_s_at	CLK1 /// PPIL3	0,84075148	4,79E-06
201225_s_at	SRRM1	0,84037881	4,85E-20
207156_at	HIST1H2AG	0,8390548	2,04E-13
208498_s_at	A /// AMY1B /// AMY1C /// AMY2A /// A	0,83865862	1,01E-15
203717_at	DPP4	0,83743602	1,80E-18
206989_s_at	SRSF2IP	0,83715419	6,30E-27
218259_at	MKL2	0,83678009	3,43E-18
204070_at	RARRES3	0,83673415	4,33E-14
219062_s_at	ZCCHC2	0,83656288	1,56E-07
204839_at	POP5	0,83607606	8,51E-20
214943_s_at	ARID4B /// RBM34	0,83555109	1,21E-22
213188_s_at	MINA	0,83490812	1,00E-22
208673_s_at	SRSF3	0,83413421	8,07E-14
218882_s_at	WDR3	0,83387822	7,20E-20
212057_at	KIAA0182	0,83380552	7,05E-18
219315_s_at	TMEM204	0,83313076	1,29E-09
218851_s_at	WDR33	0,83279948	2,47E-24
217419_x_at	AGRN	0,83255729	1,28E-24
220890_s_at	DDX47	0,83229534	9,25E-21
202232_s_at	EIF3M	0,83181423	1,21E-17
206574_s_at	PTP4A3	0,83089083	2,71E-11
200993_at	IPO7	0,83085255	2,24E-18
203428_s_at	ASF1A	0,83067329	2,66E-13
202161_at	PKN1	0,83054291	6,13E-20
33494_at	ETFDH	0,83013291	5,65E-20
201357_s_at	SF3A1	0,82998813	2,99E-16
221505_at	ANP32E	0,82970205	1,31E-06
209027_s_at	ABI1	0,82899518	2,91E-13
218389_s_at	APH1A	0,82894818	3,26E-24
221816_s_at	PHF11	0,82882655	9,10E-21
216399_s_at	SCAPER	0,82879982	1,31E-27
212099_at	RHOB	0,82847379	1,69E-12
206011_at	CASP1	0,82825281	7,90E-21
217821_s_at	WBP11	0,82687715	5,94E-17
210538_s_at	BIRC3	0,82643726	2,05E-12

205836_s_at	YTHDC2	0,82599673	2,66E-15
212507_at	TMEM131	0,82545985	1,33E-22
203263_s_at	ARHGEF9	0,8248875	9,04E-23
213906_at	MYBL1	0,82411285	2,65E-06
218204_s_at	FYCO1	0,82402954	6,37E-23
202760_s_at	AKAP2 /// PALM2-AKAP2	0,82402189	9,53E-11
206491_s_at	NAPA	0,82382824	2,06E-15
206057_x_at	SPN	0,82290493	1,97E-17
203276_at	LMNB1	0,82255508	1,52E-10
217408_at	MRPS18B	0,82243156	3,11E-26
215164_at		0,82119284	7,46E-23
209770_at	BTN3A1	0,82005804	2,17E-20
212815_at	ASCC3	0,81949464	2,46E-18
209504_s_at	PLEKHB1	0,81943002	1,31E-13
220774_at	DYM	0,81921488	4,24E-22
219291_at	DTWD1	0,81915089	4,32E-24
202968_s_at	DYRK2	0,8190091	1,27E-16
201613_s_at	AP1G2	0,8187707	2,05E-20
216278_at		0,81869147	4,51E-21
203578_s_at	SLC7A6	0,81861194	2,54E-23
212647_at	RRAS	0,81846069	5,51E-14
220926_s_at	EDEM3	0,81805076	2,26E-09
216202_s_at	SPTLC2	0,81777245	4,58E-08
204116_at	IL2RG	0,81748316	4,10E-25
215617_at	SPATS2L	0,81733438	5,23E-13
218316_at	TIMM9	0,81726624	4,34E-23
201344_at	UBE2D2	0,81678006	1,37E-19
216981_x_at	SPN	0,81634453	3,52E-18
201037_at	PFKP	0,81596825	2,55E-20
219099_at	C12orf5	0,81566598	4,74E-16
204907_s_at	BCL3	0,81522749	1,20E-12
215105_at	CG030	0,81475488	1,70E-22
202478_at	TRIB2	0,81472808	2,20E-11
210732_s_at	LGALS8	0,8143548	2,06E-07
215209_at	SEC24D	0,81432761	1,39E-22
AFFX-HUMISGF3A/M97935_MB_at	STAT1	0,81390391	6,12E-11
220925_at	NAA35	0,81311207	1,74E-17
212591_at	ARID4B /// RBM34	0,81310868	1,81E-20
202665_s_at	WIPF1	0,81268039	3,05E-21
202085_at	TJP2	0,81207266	8,77E-12
201633_s_at	CYB5B	0,81123684	8,17E-28
215207_x_at	NUS1 /// NUS1P3	0,81105883	4,50E-20
208180_s_at	HIST1H4H	0,81081218	2,63E-08
211989_at	SMARCE1	0,81048673	6,96E-25
212733_at	KIAA0226	0,81036023	9,49E-18
206357_at	OPA3	0,81010097	8,43E-27
AFFX-HUMISGF3A/M97935_MA_at	STAT1	0,80961756	9,41E-09
219885_at	SLFN12	0,80935105	7,22E-12
201990_s_at	CREBL2	0,80923927	8,17E-10
217725_x_at	SERBP1	0,80907205	1,22E-13

216298_at	TRGV5	0,80901027	4,10E-19
222203_s_at	RDH14	0,80812404	8,90E-19
217337_at		0,80802693	4,55E-21
213375_s_at	N4BP2L1	0,80744452	6,32E-11
212824_at	FUBP3	0,80734205	2,59E-16
212216_at	PREPL	0,80730126	4,75E-19
211929_at	HNRNPA3	0,80709704	1,51E-12
213126_at	MED8	0,80676147	5,68E-25
214765_s_at	NAAA	0,80605181	1,51E-12
220252_x_at	CXorf21	0,80599795	1,96E-20
202359_s_at	SNX19	0,80572782	3,62E-21
214567_s_at	XCL1 /// XCL2	0,80570096	1,92E-09
219477_s_at	THSD1 /// THSD1P1	0,80555492	6,00E-21
201156_s_at	RAB5C	0,80522364	2,35E-21
211012_s_at	GOLGA6L4 /// PML	0,80512619	8,15E-11
218593_at	RBM28	0,80487134	3,66E-27
219534_x_at	CDKN1C	0,80457886	1,44E-05
204698_at	ISG20	0,80419215	1,07E-13
213628_at	CLCC1	0,80379431	9,24E-21
217141_at	BTBD7	0,80357547	8,36E-24
218706_s_at	GRAMD3	0,80305325	8,26E-22
202625_at	LYN	0,80300562	2,28E-16
219243_at	GIMAP4	0,80281479	1,79E-25
209969_s_at	STAT1	0,80280244	1,79E-10
221536_s_at	LSG1	0,8024402	2,70E-19
222217_s_at	SLC27A3	0,80243174	2,16E-14
203935_at	ACVR1	0,80178031	1,48E-17
210948_s_at	LEF1	0,80155047	1,90E-11
218461_at	GPN3	0,80148799	2,47E-13
200953_s_at	CCND2	0,80110095	1,27E-13
220546_at	MLL	0,80102989	1,65E-21
203568_s_at	TRIM38	0,8003171	5,11E-25
203579_s_at	SLC7A6	0,80030692	9,76E-16
208203_x_at	KIR2DS5	0,80009527	3,46E-15
217840_at	DDX41	0,80002958	1,75E-20
212245_at	MCFD2	0,79976285	8,58E-15
201473_at	JUNB	0,79932327	1,01E-16
219724_s_at	KIAA0748	0,79891333	3,99E-25
204061_at	PRKX	0,79880175	4,97E-17
201501_s_at	GRSF1	0,79850695	9,17E-16
219120_at	C2orf44	0,79843101	1,46E-25
212413_at	Sep 06	0,79831647	2,57E-16
201715_s_at	ACIN1	0,79826032	7,37E-23
209654_at	KIAA0947	0,79823241	2,22E-12
205541_s_at	GSPT2	0,79794939	1,29E-16
202687_s_at	TNFSF10	0,79794939	1,54E-11
204770_at	TAP2	0,79738705	3,78E-13
201855_s_at	ATMIN	0,79734774	4,78E-15
205841_at	JAK2	0,79711754	3,72E-09
221586_s_at	E2F5	0,79698441	8,52E-21

204581_at	CD22	0,79676516	2,19E-11
209282_at	PRKD2	0,79667056	3,03E-18
218903_s_at	OBFC2B	0,79665941	5,06E-27
214959_s_at	API5	0,79600192	1,14E-18
216560_x_at	IGL@	0,79587912	1,96E-07
208843_s_at	GORASP2	0,79585256	1,51E-18
206474_at	CDK17	0,79575253	3,35E-23
207500_at	CASP5	0,79509743	2,50E-07
221345_at	FFAR2	0,79485037	6,33E-15
215046_at	C2orf67	0,7947012	1,36E-19
204143_s_at	ENOSF1	0,7946507	3,37E-13
215694_at	SPATA5L1	0,79375868	9,22E-19
218429_s_at	C19orf66	0,79325287	3,14E-17
206214_at	PLA2G7	0,79322603	2,28E-12
211325_x_at	DSTNP2	0,79321985	1,14E-23
34764_at	LARS2	0,79272274	1,23E-23
218090_s_at	WDR11	0,79243288	7,58E-14
220788_s_at	RNF31	0,79231158	2,74E-26
212415_at	Sep 06	0,79230294	3,79E-22
203610_s_at	TRIM38	0,79150898	2,30E-17
214639_s_at	HOXA1	0,79141933	4,79E-24
222316_at		0,79097355	1,06E-16
218202_x_at	MRPL44	0,79072199	1,06E-25
212152_x_at	ARID1A	0,79059608	5,03E-16
208687_x_at	HSPA8	0,79054096	1,93E-20
222010_at	TCP1	0,79034456	1,15E-24
212692_s_at	LRBA	0,79016848	2,36E-17
222014_x_at	MTO1	0,78971822	5,60E-16
217627_at	ZNF573	0,78927101	1,35E-12
209180_at	RABGGTB	0,78918613	6,50E-20
202231_at	EIF3M	0,78910189	2,55E-20
207405_s_at	RAD17	0,78840352	5,61E-20
205002_at	AHDC1	0,78837187	1,72E-18
202431_s_at	MYC	0,78821804	6,23E-11
203603_s_at	ZEB2	0,78814839	1,14E-07
212827_at	IGHM	0,78804656	5,28E-06
220646_s_at	KLRF1	0,7879661	2,82E-08
201821_s_at	TIMM17A	0,78796221	2,72E-21
200750_s_at	RAN	0,78787312	5,05E-19
202664_at	WIPF1	0,78739135	2,44E-10
213021_at	GOSR1	0,78708574	2,52E-21
202412_s_at	USP1	0,78672481	1,55E-14
216565_x_at		0,78668426	1,03E-21
203130_s_at	KIF5C	0,78558741	5,39E-20
203104_at	CSF1R	0,7851116	5,86E-11
201301_s_at	ANXA4	0,78508949	3,34E-19
209788_s_at	ERAP1	0,78497973	7,67E-12
213047_x_at	SET	0,7847694	8,62E-13
74694_s_at	RABEP2	0,7846952	2,32E-12
213882_at	TM2D1	0,78441376	7,07E-13

213069_at	HEG1	0,78423023	1,32E-19
219164_s_at	ATG2B	0,78409746	2,16E-14
220072_at	CSPP1	0,78401924	1,02E-23
214136_at	NUDT13	0,78393492	1,04E-22
218549_s_at	FAM82B	0,78373875	1,95E-15
205115_s_at	RBM19	0,78372138	9,81E-24
208754_s_at	NAP1L1	0,78347066	9,96E-09
201486_at	RCN2	0,78346589	2,09E-11
212847_at	FUBP1	0,78346274	1,41E-08
201970_s_at	NASP	0,78336313	7,29E-28
201175_at	TMX2	0,78319019	2,10E-25
47553_at	DFNB31	0,78270396	1,12E-22
218559_s_at	MAFB	0,7825611	5,24E-10
217957_at	C16orf80	0,78109382	2,60E-23
212333_at	FAM98A	0,78049867	2,85E-25
204744_s_at	IARS	0,78038155	1,20E-15
34689_at	TREX1	0,78021698	1,22E-13
211596_s_at	LRIG1	0,77970699	1,31E-12
219673_at	MCM9	0,77948932	1,04E-17
204451_at	FZD1	0,77933754	4,87E-16
212071_s_at	SPTBN1	0,77883912	1,08E-11
204197_s_at	RUNX3	0,778515	9,02E-15
210466_s_at	SERBP1	0,77848612	2,67E-21
219253_at	TMEM185B	0,7784038	1,32E-10
212819_at	ASB1	0,77795966	6,09E-20
201222_s_at	RAD23B	0,77733639	7,34E-11
205097_at	SLC26A2	0,77725784	3,27E-16
204241_at	ACOX3	0,77720248	3,11E-18
217752_s_at	CNDP2	0,77688976	1,17E-16
205789_at	CD1D	0,77658279	2,16E-11
214032_at	ZAP70	0,77573589	2,41E-11
211434_s_at	CCRL2	0,77570005	3,91E-10
202969_at	DYRK2	0,77548717	4,36E-12
221931_s_at	SEH1L	0,77527686	9,56E-15
213077_at	YTHDC2	0,77511212	1,27E-10
206513_at	AIM2	0,77498361	6,09E-06
217834_s_at	SYNCRIP	0,77483447	2,27E-23
213420_at	DHX57	0,77476658	2,23E-19
200079_s_at	KARS	0,77460523	1,18E-24
211971_s_at	LRPPRC	0,77426334	3,51E-11
211924_s_at	PLAUR	0,77313432	1,03E-13
202963_at	RFX5	0,77288245	5,29E-20
213226_at	CCNA2	0,77227056	1,63E-16
203344_s_at	RBBP8	0,77218052	5,64E-14
201063_at	RCN1	0,77200441	2,19E-28
213754_s_at	PAIP1	0,7719411	3,06E-16
202533_s_at	DHFR	0,77171104	5,41E-20
202541_at	AIMP1	0,7707015	1,15E-11
205053_at	PRIM1	0,77041043	3,58E-24
202591_s_at	SSBP1	0,77036634	1,84E-19

212200_at	ANKLE2	0,76988195	2,96E-16
206337_at	CCR7	0,76975701	1,98E-06
217501_at	CIAO1	0,7695629	1,21E-21
221542_s_at	ERLIN2	0,76953557	1,42E-16
202016_at	MEST	0,76930345	1,14E-17
208392_x_at	SP110	0,76898589	7,16E-20
200034_s_at	RPL6	0,76811036	1,83E-13
201595_s_at	ZC3H15	0,7678535	2,28E-09
212426_s_at	YWHAQ	0,76758468	3,34E-16
202505_at	SNRPB2	0,76757239	1,20E-23
210546_x_at	CTAG1A /// CTAG1B	0,76667534	1,79E-23
218185_s_at	ARMC1	0,76664514	1,95E-11
213189_at	MINA	0,76645686	9,07E-16
218356_at	FTSJ2	0,76635089	9,10E-21
217964_at	TTC19	0,76626375	2,03E-16
203517_at	MTX2	0,76603777	3,41E-17
58994_at	CC2D1A	0,76550065	4,21E-18
205003_at	DOCK4	0,76526322	5,61E-05
213269_at	ZNF248	0,76514911	1,12E-20
219481_at	TTC13	0,76508775	1,82E-17
205395_s_at	MRE11A	0,76501139	5,68E-17
201961_s_at	RNF41	0,76488211	3,71E-20
201091_s_at	CBX3	0,76416966	1,11E-10
221045_s_at	PER3	0,76391871	2,06E-21
201687_s_at	API5	0,76372731	8,79E-12
218098_at	ARFGEF2	0,76319084	4,93E-10
210046_s_at	IDH2	0,7631669	2,11E-21
202346_at	UBE2K	0,76292188	9,58E-12
212149_at	EFR3A	0,76283596	1,57E-07
213581_at	PDCD2	0,76225207	5,39E-18
203275_at	IRF2	0,76224304	1,30E-19
222192_s_at	C2orf43	0,76151963	6,30E-27
200762_at	DPYSL2	0,7615047	3,03E-11
213795_s_at	PTPRA	0,76131306	1,79E-25
219156_at	SYNJ2BP	0,76127643	3,36E-16
207871_s_at	ST7	0,76080276	2,18E-14
203022_at	RNASEH2A	0,76066362	3,24E-21
204790_at	SMAD7	0,76049659	2,90E-17
53071_s_at	C17orf101	0,76035708	2,62E-15
203696_s_at	RFC2	0,76025797	1,02E-21
203560_at	GGH	0,75972706	8,31E-13
218689_at	FANCF	0,75960763	2,32E-22
220485_s_at	SIRPG	0,75928986	5,45E-10
207798_s_at	ATXN2L	0,75903985	4,44E-21
204108_at	NFYA	0,7589456	4,64E-12
219884_at	LHX6	0,75891289	5,82E-21
200052_s_at	ILF2	0,75871646	5,73E-19
63009_at	SHQ1	0,75863655	2,75E-23
208925_at	CLDND1	0,75857228	5,48E-09
201681_s_at	DLG5	0,75811333	3,13E-17

207339_s_at	LTB	0,7573872	2,02E-15
204040_at	RNF144A	0,75736114	3,27E-17
209213_at	CBR1	0,75736088	4,05E-15
212467_at	DNAJC13	0,75701272	1,03E-12
218698_at	APIP	0,75696863	3,65E-16
218701_at	LACTB2	0,75601355	2,15E-19
203525_s_at	APC	0,7559912	3,75E-09
204172_at	CPOX	0,75501093	3,23E-10
200730_s_at	PTP4A1	0,75496494	3,71E-07
201920_at	SLC20A1	0,7541259	4,66E-22
207627_s_at	TFCP2	0,75406502	7,13E-13
201197_at	AMD1	0,75379826	6,29E-09
219677_at	SPSB1	0,75339918	3,80E-20
213573_at		0,75296881	5,48E-25
200995_at	IPO7	0,75276109	4,29E-12
222023_at	AKAP13	0,75267786	1,78E-15
219662_at	C2orf49	0,75212847	8,13E-19
215795_at	MYH7B	0,75167381	3,21E-23
210716_s_at	CLIP1	0,75129766	3,78E-11
212297_at	ATP13A3	0,75036475	3,37E-09
202265_at	BMI1	0,74982742	2,18E-07
203685_at	BCL2	0,74982181	3,77E-10
202759_s_at	AKAP2 /// PALM2-AKAP2	0,74974403	7,67E-14
210114_at	INVS	0,7497158	8,62E-25
219342_at	CASD1	0,74950076	6,84E-11
218076_s_at	ARHGAP17	0,74887322	8,06E-24
220590_at	ITFG2	0,74868544	1,02E-23
211145_x_at	IFNA21	0,74851026	5,59E-20
201833_at	HDAC2	0,74808003	1,66E-12
215777_at	IGLV4-60	0,74749316	1,49E-16
218396_at	VPS13C	0,74715682	9,23E-08
215023_s_at	PEX1	0,74659185	3,10E-21
201589_at	SMC1A	0,74644554	2,94E-18
212653_s_at	EHBP1	0,74600675	9,16E-18
218408_at	TIMM10	0,74531847	2,18E-06
212959_s_at	GNPTAB	0,74514401	2,13E-14
218350_s_at	GMNN	0,74479202	7,97E-13
213145_at	FBXL14	0,74399522	6,12E-20
207824_s_at	MAZ	0,74395457	1,22E-14
201614_s_at	RUVBL1	0,74387559	2,90E-19
205571_at	LIPT1	0,74377599	1,47E-14
206370_at	PIK3CG	0,74335245	2,09E-17
204911_s_at	TRIM3	0,74310067	2,40E-22
45288_at	ABHD6	0,74292671	4,75E-21
219913_s_at	CRNKL1	0,74285966	1,90E-10
201274_at	PSMA5	0,74278763	5,49E-28
209627_s_at	OSBPL3	0,74265686	6,56E-16
207565_s_at	MR1	0,74261076	8,46E-16
65472_at	C2orf68	0,74237008	8,37E-16
208465_at	GRM2	0,74208959	4,21E-16

217848_s_at	PPA1	0,74195318	2,88E-13
212217_at	PREPL	0,74149115	9,02E-15
219664_s_at	DECR2	0,7413732	2,66E-16
32042_at	ENOX2	0,74092872	6,87E-24
213766_x_at	GNA11	0,74067066	1,04E-22
214769_at	CLCN4	0,74054801	4,75E-15
212921_at	SMYD2	0,74031388	6,23E-13
205692_s_at	CD38	0,74013074	3,59E-08
204494_s_at	C15orf39	0,73893155	5,48E-16
206055_s_at	SNRPA1	0,7383326	1,19E-19
210029_at	IDO1	0,73816595	9,47E-05
217759_at	TRIM44	0,73781618	4,18E-19
206486_at	LAG3	0,73763233	1,69E-11
209406_at	BAG2	0,73760641	3,05E-18
205544_s_at	CR2	0,73751057	2,88E-15
218397_at	FANCL	0,73700753	3,62E-13
201669_s_at	MARCKS	0,73662458	1,06E-09
206206_at	CD180	0,73638945	5,64E-18
214396_s_at	MBD2	0,73632893	8,32E-27
201019_s_at	EIF1AX	0,73630875	9,93E-16
215351_at	RTCD1	0,73605802	4,94E-16
218260_at	DDA1	0,73593821	6,81E-23
204529_s_at	TOX	0,73563926	4,87E-13
211386_at	MGC12488	0,73559814	4,81E-21
202943_s_at	NAGA	0,73538014	1,15E-16
221561_at	SOAT1	0,73446332	1,75E-10
217899_at	TMEM214	0,73432709	5,62E-20
211902_x_at	TRD@	0,73428186	4,53E-08
203302_at	DCK	0,73332472	1,22E-05
217605_at	USP27X	0,73310111	1,05E-23
200723_s_at	CAPRIN1	0,73256808	6,87E-17
217946_s_at	SAE1	0,7320655	3,21E-21
219783_at	C2orf18	0,73196476	2,72E-19
217677_at	PLEKHA2	0,73134317	7,40E-19
215495_s_at	SAMD4A	0,73130221	1,73E-08
57539_at	ZGPAT	0,73095218	2,55E-20
205091_x_at	RECQL	0,73088373	5,54E-14
217317_s_at	HERC2P2 /// HERC2P9	0,7306991	2,58E-14
213891_s_at	TCF4	0,73050538	5,75E-14
213134_x_at	BTG3	0,73049813	2,79E-16
205898_at	CX3CR1	0,73038942	5,26E-10
218607_s_at	SDAD1	0,73019141	3,20E-21
212685_s_at	TBL2	0,73008792	4,63E-24
201214_s_at	PPP1R7	0,72988801	2,21E-20
218100_s_at	IFT57	0,72971221	6,64E-12
219374_s_at	ALG9 /// FDXACB1	0,72961121	4,72E-18
221007_s_at	FIP1L1	0,72951569	1,06E-22
211455_at		0,72939322	3,22E-21
203301_s_at	DMTF1	0,72890415	9,42E-12
203356_at	CAPN7	0,72868	2,69E-10

206660_at	IGLL1	0,72858136	1,84E-21
213528_at	C1orf156	0,72833763	1,60E-16
214873_at	LRP5L	0,72785081	3,19E-25
221020_s_at	SLC25A32	0,72782733	8,20E-12
203338_at	PPP2R5E	0,72772712	2,43E-16
203688_at	PKD2	0,7276216	4,03E-14
200764_s_at	CTNNA1	0,7274871	2,66E-11
218420_s_at	C13orf23	0,72712811	1,04E-19
210625_s_at	AKAP1	0,7268392	1,26E-16
204331_s_at	MRPS12	0,72682071	7,10E-16
219667_s_at	BANK1	0,7264975	2,56E-08
214995_s_at	APOBEC3F /// APOBEC3G	0,7264704	8,14E-17
208608_s_at	SNTB1	0,72640203	8,31E-18
209064_x_at	PAIP1	0,72628199	1,44E-16
209580_s_at	MBD4	0,72623775	9,11E-11
207303_at	PDE1C	0,7261503	6,28E-20
211339_s_at	ITK	0,72533723	5,70E-08
206759_at	FCER2	0,72514297	1,61E-11
218712_at	C1orf109	0,72500361	8,68E-17
218909_at	RPS6KC1	0,72477708	1,15E-17
208654_s_at	CD164	0,72475119	1,72E-05
202743_at	PIK3R3	0,72468763	2,49E-23
204905_s_at	EEF1E1	0,72443423	7,19E-14
212060_at	SR140	0,72410026	1,44E-11
206999_at	IL12RB2	0,72402317	9,36E-18
205758_at	CD8A	0,72391784	8,21E-08
213689_x_at	FAM69A	0,72376409	9,25E-13
204084_s_at	CLN5	0,72349358	1,27E-13
202870_s_at	CDC20	0,72348399	2,78E-09
201584_s_at	DDX39	0,72345373	3,92E-17
209567_at	RRS1	0,72330797	4,53E-21
64432_at	C12orf47	0,72320498	1,83E-15
220329_s_at	RMND1	0,72226482	2,89E-20
212568_s_at	DLAT	0,72225206	4,39E-16
200595_s_at	EIF3A	0,72211577	3,75E-10
219581_at	TSEN2	0,72202986	1,96E-17
204805_s_at	H1FX	0,72196917	8,02E-13
218455_at	NFS1	0,72135417	8,48E-24
203062_s_at	MDC1	0,72076536	9,59E-14
213666_at	Sep 06	0,720717	2,89E-11
202012_s_at	EXT2	0,72063013	7,28E-18
212872_s_at	MED20	0,72018692	1,36E-20
221229_s_at	TRMT61B	0,71997616	1,33E-12
203741_s_at	ADCY7	0,71997228	1,00E-14
208975_s_at	KPNB1	0,71989722	9,66E-18
204333_s_at	AGA	0,71985681	9,66E-12
212446_s_at	LASS6	0,71895405	2,08E-15
203607_at	INPP5F	0,71863452	1,24E-14
202579_x_at	HMGN4	0,71858389	5,56E-21
215907_at		0,71837216	1,22E-12

202816_s_at	SS18	0,71836044	6,77E-16
210031_at	CD247	0,71822283	1,67E-08
216829_at	K@ /// IGKC /// LOC652493 /// LOC6526!	0,71807291	3,06E-17
221658_s_at	IL21R	0,71804642	1,74E-16
208051_s_at	PAIP1	0,71755282	3,61E-14
210410_s_at	C6orf26 /// MSH5	0,71739692	1,49E-17
218827_s_at	CEP192	0,71730246	5,92E-14
212638_s_at	WWP1	0,71687304	5,67E-13
200823_x_at	RPL29	0,71618907	9,37E-14
209425_at	AMACR	0,71608531	4,27E-17
205642_at	CEP110	0,71592358	3,43E-20
204145_at	FRG1	0,71588208	7,76E-15
216559_x_at	1 /// HNRNPA1L2 /// HNRNPA1P10 /// LC	0,7148823	2,36E-15
207840_at	CD160	0,71484714	2,94E-10
219169_s_at	TFB1M	0,71407308	2,17E-22
218254_s_at	SAR1B	0,71404033	2,49E-07
211794_at	FYB	0,71392781	1,09E-15
202418_at	YIF1A	0,71364203	1,14E-19
205004_at	NKRF	0,71357253	2,11E-25
204512_at	HIVEP1	0,71350398	1,69E-12
209155_s_at	NT5C2	0,71338381	1,38E-17
202847_at	PCK2	0,71337327	7,12E-17
202039_at	MYO18A /// TIAF1	0,71334623	4,34E-17
215171_s_at	TIMM17A	0,71272398	2,42E-12
219833_s_at	EFHC1	0,7114416	8,72E-13
221492_s_at	ATG3	0,71124929	1,10E-15
203239_s_at	CNOT3	0,71097739	3,70E-13
212592_at	IGJ	0,7108279	0,000854655
214917_at	PRKAA1	0,71062896	1,81E-14
208918_s_at	NADK	0,71060387	3,42E-20
204441_s_at	POLA2	0,71049328	1,30E-22
204799_at	ZBED4	0,71047126	2,60E-15
38766_at	SRCAP	0,71045974	4,43E-19
46167_at	C1orf175 /// TTC4	0,71038804	2,38E-24
213361_at	TDRD7	0,71028072	3,53E-09
215245_x_at	FMR1	0,71026785	3,32E-05
221201_s_at	ZNF155	0,70949702	2,33E-23
216208_s_at	ATF6B	0,70947408	3,73E-20
207513_s_at	ZNF189	0,70910023	2,47E-15
213140_s_at	SS18L1	0,70887155	1,80E-09
219696_at	DENND1B	0,70883237	3,45E-13
219548_at	ZNF16	0,70879607	4,79E-22
210097_s_at	NOL7	0,7087458	4,23E-22
214965_at	SPATA2L	0,70857545	1,67E-16
211645_x_at		0,70810743	8,29E-05
221095_s_at	KCNE2	0,70799245	4,97E-16
204731_at	TGFBR3	0,70741133	7,73E-07
208634_s_at	MACF1	0,70711744	3,30E-13
210389_x_at	TUBD1	0,70709173	7,97E-19
219759_at	ERAP2	0,70690538	0,00034458

202524_s_at	SPOCK2	0,70671184	2,01E-08
214012_at	ERAP1	0,70596163	1,36E-08
216133_at	TRD@	0,70596054	8,40E-11
219505_at	CECR1	0,70578983	7,41E-11
214163_at	HSPB11	0,70497518	2,28E-15
210839_s_at	ENPP2	0,70493153	4,28E-20
212800_at	STX6	0,70489174	6,37E-15
218637_at	IMPACT	0,7047854	1,57E-17
218968_s_at	ZFP64	0,70471617	2,11E-22
219864_s_at	RCAN3	0,70454248	1,49E-19
213626_at	CBR4	0,70443387	6,41E-10
218957_s_at	PAAF1	0,7044093	2,12E-18
42361_g_at	CCHCR1	0,70435005	4,12E-25
202079_s_at	TRAK1	0,70413554	1,36E-15
213261_at	TRANK1	0,70385228	3,35E-13
214130_s_at	PDE4DIP	0,70377343	3,24E-17
203468_at	CDK10	0,7032564	1,94E-18
209084_s_at	RAB28	0,70315898	1,38E-11
212387_at	TCF4	0,70303221	1,47E-17
213340_s_at	KIAA0495	0,70298426	2,56E-18
201632_at	EIF2B1	0,70294326	1,27E-20
214329_x_at	TNFSF10	0,70293523	7,47E-07
203544_s_at	STAM	0,70291091	1,25E-10
202532_s_at	DHFR	0,70271761	4,02E-21
216857_at	IL23A	0,70254533	6,52E-24
203366_at	POLG	0,7022798	4,13E-20
218727_at	SLC38A7	0,7021251	4,13E-20
212381_at	USP24	0,70182535	6,85E-15
207623_at	ABCF2	0,70162725	2,17E-18
221027_s_at	PLA2G12A	0,7015751	1,36E-15
221559_s_at	MIS12	0,70156309	3,36E-13
209138_x_at	IGL@	0,70105479	8,15E-05
209340_at	UAP1	0,70102521	2,55E-09
205270_s_at	LCP2	0,70074829	8,26E-22
210959_s_at	SRD5A1	0,70070112	6,54E-13
213619_at	HNRNPH1	0,70058481	2,47E-13
209023_s_at	STAG2	0,69969661	1,55E-09
205316_at	SLC15A2	0,69915349	5,24E-13
213637_at	DDX52	0,69899919	2,50E-17
202163_s_at	CNOT8	0,69864745	3,28E-12
222371_at		0,69863892	7,65E-16
212308_at	CLASP2	0,69860699	3,94E-16
200686_s_at	SRSF11	0,69834224	1,18E-09
214852_x_at	VPS13A	0,69815384	9,94E-21
219041_s_at	REPIN1	0,69802517	7,14E-20
213183_s_at	CDKN1C	0,69767185	6,15E-06
209307_at	SWAP70	0,697086	1,64E-08
213488_at	SNED1	0,6969776	3,24E-20
200063_s_at	NPM1	0,69697428	2,35E-11
201218_at	CTBP2	0,69694566	1,06E-07

213794_s_at	NGDN	0,69677742	2,77E-21
203855_at	WDR47	0,69599349	1,04E-08
211138_s_at	KMO	0,69567275	2,59E-11
222163_s_at	SPATA5L1	0,69565401	6,51E-15
212928_at	TSPYL4	0,69545078	4,60E-13
213830_at	TRD@	0,69534176	1,30E-08
209045_at	XPNPEP1	0,69526431	9,80E-24
44822_s_at	MIER2	0,69515565	1,97E-19
209480_at	HLA-DQB1	0,69508773	0,02778111
200599_s_at	HSP90B1	0,69483639	1,31E-12
208259_x_at	IFNA7	0,69473216	2,23E-20
204545_at	PEX6	0,69416102	5,57E-11
213761_at	MDM1	0,69404155	8,96E-09
218352_at	RCBTB1	0,69384599	4,78E-08
212428_at	KIAA0368	0,69373584	1,19E-15
215371_at	MED27	0,69371967	2,36E-22
202578_s_at	DDX19A	0,69316219	1,32E-21
217313_at		0,69313987	1,77E-19
205875_s_at	TREX1	0,69311695	6,44E-09
207872_s_at	LILRA1	0,69307022	3,65E-14
217526_at	NFATC2IP	0,69264512	4,72E-15
209572_s_at	EED	0,69208353	1,84E-13
214757_at	PMS2L2	0,69173521	5,00E-20
216060_s_at	DAAM1	0,69143348	1,29E-14
209121_x_at	NR2F2	0,69041882	3,12E-18
210719_s_at	HMG20B	0,68950614	2,81E-16
203721_s_at	UTP18	0,68949887	3,90E-15
213005_s_at	KANK1	0,68944698	3,14E-12
220350_at	ZNF235	0,68895387	2,59E-20
220178_at	C19orf28	0,68894281	2,50E-22
216951_at	FCGR1A	0,68883105	2,11E-20
211563_s_at	C19orf2	0,68876017	3,91E-16
221079_s_at	METTL2A /// METTL2B	0,68843842	4,70E-18
204994_at	MX2	0,68834641	3,47E-13
220349_s_at	ENGASE	0,68829665	8,05E-17
201462_at	SCRN1	0,68818686	1,00E-14
221679_s_at	ABHD6	0,68811108	2,97E-17
212405_s_at	METTL13	0,68758043	9,12E-24
218532_s_at	FAM134B	0,68743142	8,85E-08
205583_s_at	ALG13	0,68738168	6,86E-08
212898_at	TTI1	0,68726706	5,26E-21
218738_s_at	RNF138	0,68683477	8,73E-05
212745_s_at	BBS4	0,68662617	3,60E-24
211890_x_at	CAPN3	0,68641237	9,37E-14
208819_at	RAB8A	0,68597705	5,11E-28
222216_s_at	MRPL17	0,68595721	9,55E-22
201685_s_at	TOX4	0,68548141	4,70E-14
213982_s_at	RABGAP1L	0,68547461	2,83E-13
222266_at	C19orf2	0,68533477	5,19E-10
214649_s_at	MTMR2	0,68527603	2,00E-20

221601_s_at	FAIM3	0,68522503	4,19E-07
215231_at	PRKAG2	0,68507736	3,90E-17
221311_x_at	LYRM2	0,68485408	7,34E-14
216305_s_at	C2orf3	0,68467786	8,20E-18
216863_s_at	MORC2	0,68406918	1,41E-15
205170_at	STAT2	0,68390393	1,60E-10
213364_s_at	SNX1	0,68307687	1,86E-10
200848_at	AHCYL1	0,68280722	1,69E-15
203387_s_at	TBC1D4	0,68272559	5,36E-09
213271_s_at	DOPEY1	0,68242052	9,60E-20
208839_s_at	CAND1	0,6823726	6,48E-10
204725_s_at	NCK1	0,68228347	3,50E-12
214314_s_at	EIF5B	0,68193322	2,08E-17
221653_x_at	APOL2	0,68145212	3,58E-16
221081_s_at	DENND2D	0,68140253	2,01E-13
212930_at	ATP2B1	0,68125132	1,65E-06
204701_s_at	STOML1	0,68115171	8,90E-24
208496_x_at	HIST1H3G	0,68114677	2,40E-15
212388_at	USP24	0,68090183	2,86E-18
36553_at	ASMTL	0,68084284	1,72E-13
212400_at	FAM102A	0,68056254	4,99E-08
219233_s_at	GSDMB	0,68035236	1,94E-16
212533_at	WEE1	0,68025746	1,26E-16
218515_at	GCFC1	0,6800939	3,53E-08
221768_at	LOC100506168	0,6800398	1,14E-15
212599_at	AUTS2	0,67964616	9,42E-08
206965_at	KLF12	0,67954881	1,03E-06
219200_at	FASTKD3	0,67947836	2,29E-10
205761_s_at	DUS4L	0,67913998	6,46E-20
215099_s_at	RXRB	0,67895869	4,74E-15
201522_x_at	SNRPN /// SNURF	0,67884733	1,09E-12
214794_at	PA2G4	0,67881463	2,85E-06
205267_at	POU2AF1	0,67871962	0,000140047
212324_s_at	VPS13D	0,67865089	8,70E-19
203432_at	TMPO	0,67845844	3,81E-10
204248_at	GNA11	0,67770999	1,02E-19
204593_s_at	SMCR7L	0,67696142	1,53E-17
220985_s_at	RNF170	0,67687874	6,88E-16
208304_at	CCR3	0,67681145	3,57E-05
214792_x_at	VAMP2	0,67675272	5,12E-15
203830_at	C17orf75	0,67666312	7,13E-20
208840_s_at	G3BP2	0,6757105	7,88E-12
204566_at	PPM1D	0,67570523	2,03E-10
211376_s_at	NSMCE4A	0,67545423	1,16E-17
213674_x_at	IGHD	0,67505026	0,00052969
210571_s_at	CMAH	0,6749818	1,05E-12
214475_x_at	CAPN3	0,67472418	1,70E-11
202761_s_at	SYNE2	0,67467232	4,55E-10
202714_s_at	KIAA0391	0,67438942	5,07E-20
206550_s_at	NUP155	0,67431433	4,73E-19

218885_s_at	GALNT12	0,67411897	1,66E-14
217729_s_at	AES	0,67411179	2,93E-12
218776_s_at	TMEM62	0,67409618	2,92E-21
216243_s_at	IL1RN	0,67397071	1,08E-07
221595_at	C7orf64	0,67395604	2,74E-21
210743_s_at	CDC14A	0,67369791	2,39E-17
212177_at	SFRS18	0,67346571	1,49E-12
202688_at	TNFSF10	0,67341021	3,19E-11
211031_s_at	CLIP2	0,67316018	1,16E-13
222077_s_at	RACGAP1	0,67308089	2,92E-15
204123_at	LIG3	0,67297202	9,41E-24
202624_s_at	CABIN1	0,67251212	5,47E-09
203846_at	TRIM32	0,67251006	3,21E-18
213710_s_at	CALM1	0,67226636	1,74E-19
204372_s_at	KHSRP	0,6720981	3,33E-14
203599_s_at	WBP4	0,67193454	2,39E-14
219303_at	RNF219	0,67190731	2,26E-13
202087_s_at	CTSL1	0,67186276	4,63E-11
211004_s_at	ALDH3B1	0,67135483	1,20E-13
209421_at	MSH2	0,67132861	2,61E-11
208623_s_at	EZR	0,67124915	2,63E-17
202259_s_at	N4BP2L2	0,67124164	4,79E-09
205878_at	POU6F1	0,67109117	6,65E-14
209754_s_at	TMPO	0,6710567	5,22E-09
213289_at	APOOL	0,67104725	6,17E-17
203767_s_at	STS	0,67098361	4,81E-10
217170_at		0,6709131	2,10E-19
211302_s_at	PDE4B	0,67059756	9,08E-08
218170_at	ISOC1	0,67050211	1,77E-10
218184_at	TULP4	0,67041449	2,46E-16
203992_s_at	KDM6A	0,67006195	2,69E-09
218561_s_at	LYRM4	0,67004956	1,83E-13
218166_s_at	RSF1	0,66993974	6,12E-17
215925_s_at	CD72	0,66988028	8,09E-10
209895_at	PTPN11	0,66983844	1,56E-17
203791_at	DMXL1	0,66953209	2,67E-07
206036_s_at	REL	0,66947285	1,43E-18
200039_s_at	PSMB2	0,66918877	1,67E-21
212719_at	PHLPP1	0,66913485	1,34E-09
211333_s_at	FASLG	0,66891314	9,16E-20
209471_s_at	FNTA	0,66844369	1,38E-12
208820_at	PTK2	0,66825222	9,76E-16
204804_at	TRIM21	0,66805701	2,93E-15
37549_g_at	BBS9	0,66792177	3,97E-24
203758_at	CTSO	0,6679011	9,53E-18
202978_s_at	CREBZF	0,6678261	2,81E-12
213320_at	PRMT3	0,66756121	7,84E-14
210145_at	PLA2G4A	0,66739746	1,56E-09
200056_s_at	C1D	0,66719209	2,20E-05
216421_at		0,66717977	2,77E-18

218602_s_at	HAUS6	0,66717417	2,16E-17
209813_x_at	TARP	0,66675481	5,03E-06
203664_s_at	POLR2D	0,66638151	5,99E-22
218174_s_at	C10orf57	0,66572751	2,14E-20
206655_s_at	GP1BB /// SEPT5	0,6656283	3,11E-06
203840_at	BLZF1	0,66560166	2,03E-14
210645_s_at	TTC3	0,6655216	2,21E-09
216043_x_at		0,66544068	1,81E-19
222047_s_at	SRRT	0,66517946	6,86E-16
210811_s_at	DDX49	0,66485325	2,86E-19
221642_at		0,66479759	8,28E-21
214658_at	TMED7	0,66460489	6,06E-07
204477_at	RABIF	0,66458558	3,03E-17
217956_s_at	ENOPH1	0,6641835	1,97E-14
203102_s_at	MGAT2	0,66401054	2,93E-10
200894_s_at	FKBP4	0,6637982	8,48E-15
206016_at	CCDC22	0,66369648	5,67E-13
205282_at	LRP8	0,66359564	1,17E-21
216550_x_at	ANKRD12	0,66353093	3,47E-11
203245_s_at	NCRNA00094	0,66332579	4,92E-17
215212_at		0,66298519	1,49E-12
220099_s_at	LUC7L2	0,66293771	8,53E-18
201656_at	ITGA6	0,66257333	5,05E-11
219901_at	FGD6	0,66239729	2,45E-21
214975_s_at	MTMR1	0,66207002	3,82E-07
205071_x_at	XRCC4	0,66187366	4,47E-15
207830_s_at	PPP1R8	0,66183685	1,89E-18
202786_at	STK39	0,66181061	1,43E-12
215708_s_at	PRIM2	0,66140891	7,12E-19
203203_s_at	KRR1	0,66132612	3,37E-14
214643_x_at	BIN1	0,66129624	6,20E-18
208988_at	KDM2A	0,66121997	2,94E-18
202314_at	CYP51A1	0,66079752	2,70E-14
210299_s_at	FHL1	0,66062919	4,10E-10
215743_at	NMT2	0,66010331	3,49E-13
201626_at	INSIG1	0,65978611	2,50E-11
207556_s_at	DGKZ	0,65975388	1,66E-12
216652_s_at	DR1	0,65969605	1,57E-10
209974_s_at	BUB3	0,65966034	5,33E-17
204699_s_at	C1orf107	0,65962296	1,41E-22
206150_at	CD27	0,65951836	9,65E-07
203086_at	KIF2A	0,65897599	2,22E-14
206715_at	TFEC	0,65893897	0,000484345
201892_s_at	IMPDH2	0,65889117	1,12E-09
205590_at	RASGRP1	0,65879017	7,49E-07
206662_at	GLRX	0,6587567	1,30E-10
202623_at	EAPP	0,65853387	1,41E-14
218477_at	TMEM14A	0,6583963	2,76E-14
208078_s_at	SIK1	0,6581228	3,33E-14
212633_at	KIAA0776	0,65795622	2,12E-07

215121_x_at	IGLC7 /// IGLV1-44 /// LOC100290481	0,65786749	3,51E-05
217976_s_at	DYNC1LI1	0,6577909	8,96E-10
209723_at	SERPINB9	0,65772599	3,93E-12
208657_s_at	Sep 09	0,65747824	5,04E-12
204125_at	NDUFAF1	0,65729619	6,28E-14
218282_at	EDEM2	0,65724003	1,19E-18
204234_s_at	ZNF195	0,65708859	3,06E-16
207877_s_at	NVL	0,65688003	5,84E-20
209671_x_at	TRAC	0,65667485	2,38E-07
219818_s_at	GPATCH1	0,65648777	8,53E-19
207181_s_at	CASP7	0,65644628	8,01E-12
41858_at	PGAP2	0,65640785	2,09E-20
220419_s_at	USP25	0,65632379	7,45E-13
218467_at	PSMG2	0,6553899	2,01E-22
215646_s_at	VCAN	0,65519472	4,84E-05
215848_at	SCAPER	0,65505582	1,60E-18
212300_at	TXLNA	0,65497689	3,35E-19
201437_s_at	EIF4E	0,65490499	8,99E-10
214359_s_at	HSP90AB1	0,65482423	9,02E-10
206090_s_at	DISC1 /// TSNAX-DISC1	0,65478668	3,14E-11
216713_at	KRIT1	0,65468601	4,17E-12
207688_s_at		0,65467791	4,85E-15
212367_at	FEM1B	0,65427662	1,76E-10
201014_s_at	PAICS	0,65417306	3,18E-16
212841_s_at	PPFIBP2	0,65403817	3,95E-14
211801_x_at	MFN1	0,65401789	6,34E-15
219648_at	MREG	0,65397846	5,32E-13
220969_s_at		0,65392325	5,18E-15
210985_s_at	SP100	0,65378562	5,78E-08
211761_s_at	CACYBP	0,65375744	1,35E-12
201760_s_at	WSB2	0,65325931	7,24E-13
213024_at	TMF1	0,65315374	7,32E-08
209935_at	ATP2C1	0,65314332	5,89E-16
203417_at	MFAP2	0,65301377	3,44E-18
213144_at	GOSR2	0,6527716	6,19E-25
201310_s_at	C5orf13	0,65241356	1,60E-16
221827_at	RBCK1	0,65233366	3,53E-14
207314_x_at	KIR3DL1 /// KIR3DL2 /// LOC727787	0,65227562	2,85E-08
204274_at	EBAG9	0,65218011	1,64E-12
213600_at	SIPA1L3	0,65190175	5,09E-19
218288_s_at	CCDC90B	0,65161422	7,20E-11
203373_at	SOCS2	0,65106774	1,93E-10
213022_s_at	UTRN	0,65085602	3,81E-18
217803_at	GOLPH3	0,65076408	1,65E-10
219465_at	APOA2	0,65072624	1,75E-21
218616_at	INTS12	0,65071013	6,51E-21
217916_s_at	FAM49B	0,650695	5,37E-13
212927_at	SMC5	0,6505659	1,41E-14
207826_s_at	ID3	0,64993333	2,13E-11
208109_s_at	C15orf5	0,64954351	6,90E-15

217364_x_at		0,64951491	1,70E-20
201461_s_at	MAPKAPK2	0,64942157	1,51E-08
204493_at	BID	0,64923296	8,46E-14
218348_s_at	ZC3H7A	0,64907889	3,11E-09
218764_at	PRKCH	0,64888219	7,12E-11
203803_at	PCYOX1	0,64886535	2,37E-11
206316_s_at	KNTC1	0,64859889	1,37E-20
202602_s_at	HTATSF1	0,64819165	2,87E-11
205763_s_at	DDX18	0,64791651	4,02E-15
200676_s_at	UBE2L3	0,64786847	2,67E-15
201157_s_at	NMT1	0,64777655	1,58E-22
220235_s_at	C1orf103	0,6476055	8,37E-07
218948_at	QRSL1	0,64753253	7,26E-15
215483_at	AKAP9	0,64729339	1,08E-08
213620_s_at	ICAM2	0,64709362	5,34E-13
203110_at	PTK2B	0,64662984	1,89E-17
215772_x_at	SUCLG2	0,64656393	2,89E-11
206038_s_at	NR2C2	0,64637387	1,72E-19
218438_s_at	MED28	0,64594067	1,49E-14
201031_s_at	HNRNPH1	0,64587251	1,80E-12
202367_at	CUX1	0,6458474	1,11E-12
210514_x_at	HLA-G	0,64582671	1,69E-25
49485_at	PRDM4	0,64577562	3,68E-25
214221_at	ALMS1	0,64512941	1,16E-20
214140_at	SLC25A16	0,64480474	4,56E-23
204433_s_at	SPATA2	0,64464368	1,31E-17
219528_s_at	BCL11B	0,64453854	4,62E-06
203044_at	CHSY1	0,64449317	9,05E-06
217690_at	LOC100505523	0,64447645	5,42E-20
203343_at	UGDH	0,64382807	1,23E-15
36711_at	MAFF	0,64366152	1,99E-06
213704_at	RABGGTB	0,64365714	2,08E-12
202633_at	TOPBP1	0,64362917	5,18E-12
200893_at	TRA2B	0,64359033	3,27E-14
218149_s_at	ZNF395	0,64349443	4,47E-08
203970_s_at	PEX3	0,64322724	1,26E-11
200683_s_at	UBE2L3	0,64316183	8,69E-24
201297_s_at	MOBKL1B	0,64279127	7,81E-07
204853_at	ORC2	0,64271916	6,88E-15
202850_at	ABCD3	0,64206245	1,57E-07
200798_x_at	MCL1	0,64167228	6,63E-09
212305_s_at	MIA3	0,6416241	3,22E-17
211746_x_at	PSMA1	0,64139793	1,03E-21
218442_at	TTC4	0,64106689	2,06E-22
218478_s_at	ZCCHC8	0,64106425	1,42E-12
216929_x_at	ABO	0,64102646	6,39E-13
213330_s_at	STIP1	0,64090082	1,08E-13
200787_s_at	PEA15	0,64069665	1,89E-16
211144_x_at	TARP /// TRGC2	0,640376	1,14E-05
209330_s_at	HNRNPD	0,64037411	5,30E-13

213218_at	ZNF187	0,64033815	5,64E-19
203730_s_at	ZKSCAN5	0,64033655	3,77E-20
207828_s_at	CENPF	0,64032072	4,49E-15
210717_at		0,64026947	3,09E-15
205306_x_at	KMO	0,64024542	7,17E-09
219322_s_at	WDR8	0,63994942	1,44E-18
211641_x_at	GHG1 /// IGHG3 /// IGHM /// IGHV3-48 ///	0,63979911	7,17E-10
219083_at	SHQ1	0,63968975	2,50E-16
215470_at	GTF2H2B	0,639538	8,42E-08
201853_s_at	CDC25B	0,63936804	9,95E-08
210164_at	GZMB	0,63927354	8,85E-07
209881_s_at	LAT /// SPNS1	0,63924796	5,20E-12
214830_at	SLC38A6	0,6391715	2,62E-15
203964_at	NMI	0,63894275	3,83E-12
215160_x_at	100509419 /// LOC100510581 /// LOC642	0,6386264	1,17E-17
208886_at	H1F0	0,63837347	1,30E-06
203801_at	MRPS14	0,63834673	1,02E-15
215548_s_at	SCFD1	0,63827682	2,44E-11
217052_x_at		0,63813262	1,89E-15
211790_s_at	MLL2	0,63802204	5,48E-18
222120_at	ZNF764	0,63784968	3,69E-24
212706_at	RASA4	0,63753277	2,82E-06
204828_at	RAD9A	0,63741555	1,42E-20
209595_at	GTF2F2	0,63736964	8,99E-19
205407_at	RECK	0,63734321	1,94E-12
212693_at	MDN1	0,63722031	1,29E-14
212644_s_at	MAPK1IP1L	0,6371014	5,36E-21
201512_s_at	TOMM70A	0,63709961	6,13E-18
204912_at	IL10RA	0,63690627	3,80E-14
218519_at	SLC35A5	0,63688468	2,95E-08
204062_s_at	ULK2	0,63678516	1,11E-15
204957_at	ORC5	0,63673378	9,53E-14
200873_s_at	CCT8	0,63669515	1,15E-17
215151_at	DOCK10	0,63659726	3,18E-15
206308_at	TRDMT1	0,63652616	1,39E-15
206613_s_at	TAF1A	0,63613725	6,08E-18
203816_at	DGUOK	0,63613066	4,47E-22
201601_x_at	IFITM1	0,63544341	8,91E-22
210224_at	MR1	0,63510761	4,84E-18
202506_at	SSFA2	0,6350076	3,28E-06
204894_s_at	AOC3	0,63498523	2,37E-10
210009_s_at	GOSR2	0,63490304	3,27E-21
209814_at	ZNF330	0,63475126	5,95E-12
218167_at	AMZ2	0,63462111	7,61E-17
202784_s_at	NNT	0,6345858	2,19E-11
212262_at	QKI	0,63455521	3,37E-11
218156_s_at	TSR1	0,63454471	7,15E-20
211038_s_at	CROCCP2	0,63428713	2,90E-09
212590_at	RRAS2	0,63415709	1,00E-13
205400_at	WAS	0,63413616	7,70E-11

221510_s_at	GLS	0,63393998	8,20E-05
215044_s_at	STAM2	0,63389482	1,35E-11
203077_s_at	SMAD2	0,63336423	8,90E-12
205718_at	ITGB7	0,63326146	7,35E-13
204718_at	EPHB6	0,63325612	2,44E-11
207920_x_at	ZFX	0,63291124	1,73E-13
209354_at	TNFRSF14	0,63280309	7,91E-22
210847_x_at	TNFRSF25	0,63279578	7,59E-09
204392_at	CAMK1	0,63273121	4,14E-11
218496_at	RNASEH1	0,63253044	1,16E-15
209509_s_at	DPAGT1	0,63249042	5,78E-15
212202_s_at	TMEM87A	0,63243945	6,86E-26
217599_s_at	MDFIC	0,63221242	1,51E-17
39248_at	AQP3	0,63221174	1,62E-05
203576_at	BCAT2	0,63164483	2,07E-16
217124_at	IQCE	0,63140929	3,61E-15
219799_s_at	DHRS9	0,63127193	4,07E-06
211447_s_at	PDE4A	0,63083934	1,41E-17
209036_s_at	MDH2	0,63080362	1,07E-17
218505_at	WDR59	0,63069473	6,80E-15
222000_at	C1orf174	0,63064121	3,03E-17
219571_s_at	ZNF12	0,63041234	5,56E-07
207943_x_at	PLAGL1	0,63039841	8,89E-10
215211_at	RRN3P1	0,63003538	3,30E-13
212486_s_at	FYN	0,6299675	1,10E-09
218645_at	ZNF277	0,62992254	2,68E-11
41660_at	CELSR1	0,62969827	1,29E-21
208907_s_at	MRPS18B	0,62963707	1,89E-18
201770_at	SNRPA	0,62946337	1,19E-13
212762_s_at	TCF7L2	0,62941376	1,00E-08
202068_s_at	LDLR	0,62932239	1,37E-10
218668_s_at	RAP2C	0,62903233	3,05E-05
221978_at	HLA-F	0,6286383	6,18E-22
213803_at	KPNB1	0,62855252	6,26E-19
203882_at	IRF9	0,62840333	3,62E-23
212272_at	LPIN1	0,62828338	2,76E-16
209123_at	QDPR	0,62826194	1,58E-15
218640_s_at	PLEKHF2	0,62819752	0,000245438
205901_at	PNOC	0,62774267	1,27E-13
216396_s_at	EI24	0,62767962	1,05E-20
203418_at	CCNA2	0,62750423	2,71E-12
211960_s_at	RAB7A	0,62741598	7,84E-15
216807_at	KIAA1751	0,62739641	4,28E-17
209181_s_at	RABGGTB	0,62730011	3,39E-10
212350_at	TBC1D1	0,62716373	2,80E-15
220710_at	C15orf28	0,62702451	1,92E-08
216600_x_at	ALDOB	0,62667914	1,77E-20
208453_s_at	XPNPEP1	0,62646804	4,72E-20
202060_at	CTR9	0,62627168	1,67E-10
214173_x_at	C19orf2	0,62592998	1,76E-14

206082_at	HCP5	0,62590564	1,68E-17
211704_s_at	SPIN2A /// SPIN2B	0,62588003	5,32E-20
201816_s_at	GBAS	0,62567822	3,70E-08
220911_s_at	NYNRIN	0,62537673	3,44E-18
221869_at	ZNF512B	0,62525731	1,00E-19
203357_s_at	CAPN7	0,62525229	7,42E-10
204244_s_at	DBF4	0,62518164	5,48E-12
211063_s_at	NCK1	0,62515162	1,12E-09
213038_at	RNF19B	0,62497063	6,20E-13
204873_at	PEX1	0,62476059	2,60E-15
202642_s_at	TRRAP	0,62449549	5,83E-12
201849_at	BNIP3	0,62438788	1,99E-10
216306_x_at	PTBP1	0,62437365	2,44E-16
212553_at	RPRD2	0,62418623	3,72E-15
218545_at	CCDC91	0,62389864	4,03E-08
206288_at	PGGT1B	0,62358762	8,24E-15
208894_at	HLA-DRA	0,62350269	6,63E-11
209884_s_at	SLC4A7	0,62350102	6,76E-08
221570_s_at	METTL5	0,62342275	1,67E-18
215269_at	TRAPPC10	0,62334367	3,22E-15
213193_x_at	TRBC1	0,62331667	6,40E-07
205861_at	SPIB	0,62301312	2,23E-08
216629_at	SRRM2	0,62296368	1,17E-12
209761_s_at	SP110	0,6229632	4,21E-18
209903_s_at	ATR	0,62283688	1,55E-10
204156_at	SIK3	0,62277952	1,38E-10
210062_s_at	ZNF589	0,62249545	5,24E-13
207164_s_at	ZNF238	0,62233182	1,01E-09
212779_at	KIAA1109	0,62206939	6,94E-05
209995_s_at	TCL1A	0,62206273	0,000286525
210283_x_at	PAIP1	0,62199362	8,12E-14
202558_s_at	HSPA13	0,62198447	1,87E-09
213445_at	ZC3H3	0,62102666	5,76E-13
218349_s_at	ZWILCH	0,62091806	7,22E-16
209250_at	DEGS1	0,62058487	4,31E-09
213564_x_at	LDHB	0,6205255	4,45E-07
212610_at	PTPN11	0,61998468	1,73E-07
220370_s_at	USP36	0,61990675	6,14E-19
201676_x_at	PSMA1	0,61915629	2,10E-21
209404_s_at	TMED7	0,61909708	3,58E-09
209902_at	ATR	0,61901182	3,72E-16
220684_at	TBX21	0,61891435	2,65E-08
203209_at	RFC5	0,61888796	1,23E-14
201930_at	MCM6	0,618666	2,61E-11
212424_at	PDCD11	0,61858081	6,76E-16
212345_s_at	CREB3L2	0,61827694	3,78E-16
206583_at	ZNF673	0,61824606	1,92E-17
217988_at	CCNB1IP1	0,61816785	1,22E-11
202491_s_at	IKBKAP	0,61795428	1,58E-12
219008_at	C2orf43	0,61789683	3,58E-13

203818_s_at	SF3A3	0,6178618	1,02E-14
204891_s_at	LCK	0,61783869	2,94E-06
214252_s_at	CLN5	0,61772853	1,21E-16
202710_at	BET1	0,61733707	2,03E-12
209407_s_at	DEAF1	0,6173012	2,34E-18
218534_s_at	AGGF1	0,61717328	2,73E-10
206129_s_at	ARSB	0,61705502	8,24E-15
202113_s_at	SNX2	0,61702864	4,97E-09
201848_s_at	BNIP3	0,61603035	4,41E-17
205585_at	ETV6	0,6160222	4,19E-14
212871_at	MAPKAPK5	0,61599353	4,01E-13
205425_at	HIP1	0,61597129	4,38E-05
204083_s_at	TPM2	0,61588268	2,17E-13
202251_at	PRPF3	0,61527199	1,45E-20
214315_x_at	CALR	0,61522681	8,45E-16
219530_at	PALB2	0,61522564	1,93E-18
201035_s_at	HADH	0,61516991	8,20E-23
204166_at	SBNO2	0,61509158	1,83E-09
209392_at	ENPP2	0,61490598	2,49E-10
221925_s_at	CSPP1	0,61483533	3,18E-21
202207_at	ARL4C	0,61473011	1,93E-08
204923_at	SASH3	0,61449649	6,40E-24
213534_s_at	PASK	0,61442625	1,69E-06
206542_s_at	SMARCA2	0,61412491	1,67E-08
203581_at	RAB4A	0,61395948	5,00E-11
202979_s_at	CREBZF	0,61393202	1,77E-06
212998_x_at	HLA-DQB1 /// LOC100133583	0,61384912	1,00E-05
202944_at	NAGA	0,61359956	3,38E-12
204149_s_at	GSTM4	0,61344553	4,03E-07
204769_s_at	TAP2	0,61319289	2,51E-09
201216_at	ERP29	0,61278752	1,89E-15
210865_at	FASLG	0,61269972	7,88E-16
201896_s_at	PSRC1	0,61232319	2,25E-16
220408_x_at	FAM48A	0,6122464	1,04E-15
210102_at	VWA5A	0,61181579	2,99E-09
217927_at	SPCS1	0,61152885	2,54E-17
207460_at	GZMM	0,61146591	9,05E-07
217851_s_at	SLMO2	0,61096083	3,91E-11
200602_at	APP	0,61084443	1,68E-08
209604_s_at	GATA3	0,6100598	7,46E-08
221260_s_at	CSRNP2	0,60977763	8,90E-17
207091_at	P2RX7	0,60972093	1,10E-10
217540_at	FAM55C	0,60930415	2,18E-10
216064_s_at	AGA	0,60919753	1,31E-10
204759_at	RCBTB2	0,6090724	7,94E-08
215075_s_at	GRB2	0,60872037	5,44E-17
220500_s_at	RABL2A /// RABL2B	0,60871713	8,49E-17
217942_at	MRPS35	0,60849535	6,72E-14
205732_s_at	NCOA2	0,6084244	1,49E-07
210813_s_at	XRCC4	0,60836932	1,39E-12

209357_at	CITED2	0,60816179	1,27E-14
204929_s_at	VAMP5	0,60799982	7,74E-07
205449_at	SAC3D1	0,60790394	2,04E-15
212604_at	MRPS31	0,60761927	1,71E-11
203046_s_at	TIMELESS	0,60761767	2,14E-23
216667_at	ECRP /// RNASE2	0,60755244	4,25E-07
209459_s_at	ABAT	0,60749448	3,68E-05
213135_at	TIAM1	0,60744784	1,80E-06
205169_at	RBBP5	0,60727373	1,09E-14
201378_s_at	UBAP2L	0,60724598	1,09E-16
203032_s_at	FH	0,60713109	2,43E-18
213588_x_at	RPL14	0,60695521	1,29E-11
219383_at	PRR5L	0,60679437	2,79E-10
206761_at	CD96	0,60672536	8,20E-09
205324_s_at	FTSJ1	0,60670371	6,87E-14
219555_s_at	CENPN	0,6065755	4,40E-17
202585_s_at	NFX1	0,60654414	1,69E-20
32062_at	LRRC14	0,60591059	7,55E-18
78495_at	ZNF783	0,60536176	1,51E-20
217667_at	LOC729799	0,6052039	1,78E-19
202717_s_at	CDC16	0,6051769	6,02E-13
219881_s_at	LOC100507619	0,60504024	5,68E-25
218784_s_at	C6orf64	0,60490456	1,21E-22
202221_s_at	EP300	0,60485337	1,92E-10
214499_s_at	BCLAF1	0,60474808	2,68E-11
212279_at	TMEM97	0,60458943	4,78E-16
207819_s_at	ABCB4	0,60447924	9,35E-14
216232_s_at	GCN1L1	0,60425236	6,37E-16
202594_at	LEPROTL1	0,6040721	3,07E-06
213049_at	RALGAPA1	0,60404094	1,30E-07
202662_s_at	ITPR2	0,60388921	3,42E-13
57082_at	LDLRAP1	0,60387263	2,76E-08
204003_s_at	NUPL2	0,6037643	1,24E-21
220761_s_at	TAOK3	0,60369995	3,46E-17
212310_at	MIA3	0,60356002	1,71E-10
218913_s_at	GMIP	0,60327708	3,47E-15
201646_at	SCARB2	0,60311812	5,12E-09
218362_s_at	DIS3	0,60294747	3,24E-15
211483_x_at	CAMK2B	0,60276552	9,74E-17
217808_s_at	MAPKAP1	0,60257218	9,50E-18
204948_s_at	FST	0,60240678	6,43E-16
219110_at	GAR1	0,60227856	1,51E-16
206295_at	IL18	0,602129	3,58E-15
203406_at	MFAP1	0,60140074	3,82E-28
209606_at	CYTIP	0,60136985	1,27E-20
211687_x_at	KIR3DL1	0,60133051	1,51E-09
211947_s_at	BAT2L2	0,60117916	1,14E-16
213919_at	DNAJC4	0,60071119	2,12E-16
210502_s_at	PPIE	0,6007008	8,39E-17
213939_s_at	RUFY3	0,60053343	5,19E-09

220078_at	USP48	0,60048639	1,47E-14
216207_x_at	GKV1-5 /// IGKV1D-8 /// LOC652493 /// L	0,60046308	2,22E-05
65585_at	FAM86B1	0,60031106	1,56E-14
218082_s_at	UBP1	0,6002414	5,01E-13
217911_s_at	BAG3	0,60020645	1,07E-10
219812_at	PVRIG	0,59999333	7,05E-07
204332_s_at	AGA	0,59998895	9,60E-14
212864_at	CDS2	0,59961173	1,07E-11
220052_s_at	TINF2	0,59906815	1,74E-25
222103_at	ATF1	0,5987335	1,10E-05
221808_at	RAB9A	0,59854692	3,15E-14
208966_x_at	IFI16	0,59851354	3,55E-12
211889_x_at	CEACAM1	0,5982368	0,000400314
206891_at	ACTN3	0,59822527	3,65E-16
221637_s_at	C11orf48	0,59818044	5,90E-20
221575_at	SCLY	0,59800351	3,11E-20
219207_at	EDC3	0,59792427	8,30E-18
209388_at	PAPOLA	0,59774598	6,55E-08
201518_at	CBX1	0,59759963	4,06E-10
212673_at	METAP1	0,59731825	1,33E-19
218093_s_at	ANKRD10	0,59731669	6,96E-09
217366_at	CTNNA1	0,59718768	2,03E-21
219366_at	AVEN	0,59705091	3,09E-20
219343_at	CDC37L1	0,59680013	5,37E-13
204346_s_at	RASSF1	0,59611371	1,63E-15
203828_s_at	IL32	0,59598187	1,05E-05
201252_at	PSMC4	0,59591509	1,66E-21
222310_at	SFRS15	0,59581409	2,11E-10
202722_s_at	GFPT1	0,59569667	4,69E-14
212519_at	UBE2E1	0,59569435	3,32E-10
212902_at	SEC24A	0,59564976	3,14E-11
208013_s_at	ACRV1	0,59563873	5,89E-23
205124_at	LOC729991-MEF2B /// MEF2B	0,59555654	2,25E-12
222122_s_at	THOC2	0,59525028	1,65E-22
217813_s_at	SPIN1	0,59524313	4,11E-17
216950_s_at	FCGR1A /// FCGR1C	0,59523721	0,000597074
206634_at	SIX3	0,59523079	1,85E-17
217422_s_at	CD22	0,59507068	7,82E-07
202951_at	STK38	0,5950153	6,33E-21
204286_s_at	PMAIP1	0,5947124	7,86E-07
222037_at	MCM4	0,59448592	2,27E-18
219380_x_at	POLH	0,59446608	3,01E-23
217914_at	TPCN1	0,59431839	1,67E-09
64474_g_at	DGCR8	0,59420355	5,50E-15
201459_at	RUVBL2	0,59404016	3,76E-17
202840_at	TAF15	0,5937752	1,24E-17
219806_s_at	C11orf75	0,59375319	3,52E-09
209748_at	SPAST	0,59338734	5,36E-06
213199_at	C2CD3	0,5933297	3,44E-24
218209_s_at	RPRD1A	0,5933136	1,49E-11

219922_s_at	LTBP3	0,59308953	1,49E-06
212139_at	GCN1L1	0,59276047	1,54E-13
222209_s_at	TMEM135	0,59258438	2,84E-08
215888_at	PDS5B	0,59250479	2,50E-13
214402_s_at	SFI1	0,59243724	1,52E-18
209570_s_at	D4S234E	0,59224615	7,25E-15
205404_at	HSD11B1	0,59209215	2,44E-20
205233_s_at	PAFAH2	0,59203408	6,82E-23
212696_s_at	RNF4	0,59203191	2,07E-20
204127_at	RFC3	0,59203094	5,69E-13
213073_at	ZFYVE26	0,59201896	1,66E-11
203339_at	SLC25A12	0,59193809	1,56E-18
211881_x_at	IGLJ3	0,59156106	9,12E-08
213374_x_at	HIBCH	0,59156001	9,72E-14
203647_s_at	FDX1	0,59140572	1,17E-13
201163_s_at	IGFBP7	0,59119888	1,24E-07
205684_s_at	DENND4C	0,5910938	1,71E-20
204415_at	IFI6	0,59101519	0,007741947
202583_s_at	RANBP9	0,59090006	8,44E-07
204991_s_at	NF2	0,59089413	3,24E-21
215873_x_at	ABCC10	0,5908573	1,41E-20
218928_s_at	SLC37A1	0,59076695	1,34E-23
201586_s_at	SFPQ	0,59072628	1,01E-12
206028_s_at	MERTK	0,59062028	9,27E-08
220674_at	CD22	0,59057215	2,49E-17
206641_at	TNFRSF17	0,59053245	0,000132303
213266_at	TUBGCP4	0,59037677	1,74E-18
208858_s_at	ESYT1	0,5902791	5,89E-08
203552_at	MAP4K5	0,59023205	3,75E-06
204012_s_at	LCMT2	0,59017596	4,30E-21
219822_at	MTRF1	0,59006696	6,15E-18
207350_s_at	VAMP4	0,58999779	7,88E-12
201245_s_at	OTUB1	0,58972858	5,77E-23
203250_at	RBM16	0,58953047	3,16E-09
212090_at	GRINA	0,58858651	2,23E-07
218757_s_at	UPF3B	0,58856091	1,52E-12
221156_x_at	CCPG1	0,58831528	4,18E-05
218868_at	ACTR3B	0,58816232	4,96E-23
204192_at	CD37	0,58808724	4,67E-13
204807_at	TMEM5	0,58795984	1,84E-19
212490_at	DNAJC8	0,58791455	7,44E-21
200608_s_at	RAD21	0,58760538	5,34E-06
212660_at	PHF15	0,58752016	1,43E-13
214967_at		0,58733779	7,97E-11
215716_s_at	ATP2B1	0,58685094	9,58E-06
202797_at	SACM1L	0,58653559	4,76E-05
207153_s_at	GLMN	0,58653501	5,28E-19
217147_s_at	TRAT1	0,58645775	7,86E-07
214274_s_at	ACAA1	0,58642586	5,28E-15
209424_s_at	AMACR /// C1QTNF3	0,58622889	2,02E-16

212563_at	BOP1	0,58621396	2,74E-14
221571_at	TRAF3	0,58550632	8,77E-12
213142_x_at	PION	0,58546365	7,41E-07
213463_s_at	FAM149B1	0,58539079	5,67E-14
222189_at		0,58535164	3,29E-22
207150_at	SLC18A3	-0,58513388	3,95E-20
221084_at	HTR3B	-0,58521929	2,40E-17
215029_at		-0,58568814	2,93E-09
222213_x_at		-0,58581207	4,18E-20
202581_at	HSPA1A /// HSPA1B	-0,58600107	6,76E-05
220693_at	CCDC82	-0,58601643	3,96E-23
207384_at	PGLYRP1	-0,586184	0,000159024
210161_at		-0,5862147	1,29E-18
214301_s_at	DPYSL4	-0,58629313	1,64E-18
216340_s_at	CYP2A7P1	-0,58645842	4,93E-20
212283_at	AGRN	-0,58653416	1,30E-16
215579_at	APOBEC3G	-0,58682529	5,04E-13
218162_at	OLFML3	-0,58707173	4,53E-19
213044_at	ROCK1	-0,5871765	5,98E-05
215737_x_at	USF2	-0,58745808	1,70E-17
214372_x_at	ERN2	-0,58751574	5,84E-18
202467_s_at	COPS2	-0,58802812	0,00016574
215780_s_at	LOC642869 /// SET	-0,5883397	9,31E-07
203123_s_at	SLC11A2	-0,588415	1,01E-10
211603_s_at	ETV4	-0,58843321	1,51E-17
219449_s_at	TMEM70	-0,5884448	1,29E-09
203601_s_at	ZBTB17	-0,5885374	2,73E-19
201182_s_at	CHD4	-0,5885638	1,20E-21
201710_at	MYBL2	-0,58868333	6,59E-12
38671_at	PLXND1	-0,58884058	1,15E-15
205759_s_at	SULT2B1	-0,58888788	1,88E-21
217053_x_at	ETV1	-0,58892421	2,43E-17
207376_at	VENTX	-0,58901898	1,45E-17
219247_s_at	ZDHHC14	-0,58914759	5,88E-16
209987_s_at	ASCL1	-0,58927018	7,98E-23
211872_s_at	RGS11	-0,58956118	3,35E-20
210454_s_at	KCNJ6	-0,59005349	8,88E-19
216187_x_at		-0,59013339	2,16E-10
203369_x_at	PDLIM7	-0,59029102	1,50E-14
212136_at	ATP2B4	-0,59064233	8,77E-13
217520_x_at	LOC283683	-0,59079505	1,46E-21
220256_s_at	OXCT2	-0,59131674	1,43E-19
209614_at	ADH1B	-0,59134932	4,15E-22
202782_s_at	INPP5K	-0,59137108	5,13E-15
219227_at	CCNJL	-0,59147755	7,88E-08
218357_s_at	TIMM8B	-0,5915127	4,62E-09
211873_s_at	PCDHGA9	-0,59189982	1,54E-19
221350_at	HOXC8	-0,59194562	4,78E-16
210515_at	HNF1A	-0,59209375	5,15E-19
221555_x_at	CDC14B	-0,59237743	1,03E-13

212477_at	ACAP2	-0,59240368	9,54E-13
211560_s_at	ALAS2	-0,59262693	0,000117731
214224_s_at	PIN4	-0,59265611	1,02E-15
206530_at	RAB30	-0,59266482	2,08E-19
220962_s_at	PADI1	-0,59276946	3,38E-16
215681_at	KIAA1654	-0,59285809	5,61E-20
213290_at	COL6A2	-0,59297843	5,39E-19
207025_at	GJC2	-0,59309914	1,49E-14
210739_x_at	SLC4A4	-0,59346168	6,88E-19
216548_x_at	HMGB3P1	-0,59359343	4,37E-19
219275_at	PDCD5	-0,59359893	4,28E-17
217370_x_at	FUS	-0,59376752	2,35E-14
209514_s_at	RAB27A	-0,59378978	2,98E-11
211157_at		-0,59404801	3,87E-19
200837_at	BCAP31	-0,59407567	4,74E-16
206955_at	AQP7	-0,59425999	1,19E-15
216128_at	TBCD	-0,59428985	8,17E-24
209391_at	DPM2	-0,59440791	5,10E-06
204975_at	EMP2	-0,59461603	3,91E-21
AFFX-PheX-3_at		-0,59470743	1,00E-24
219942_at	MYL7	-0,59475808	3,38E-18
217669_s_at	AKAP6	-0,59479654	4,12E-23
216953_s_at	WT1	-0,5949654	2,63E-22
218335_x_at	TNIP2	-0,59516287	4,01E-19
203470_s_at	PLEK	-0,59565346	2,78E-06
215064_at	SC5DL	-0,59580968	1,58E-23
202102_s_at	BRD4	-0,59646857	7,54E-12
215137_at		-0,59655703	1,08E-18
209415_at	FZR1	-0,59656265	7,56E-18
207993_s_at	CHP	-0,59660599	8,93E-15
221355_at	CHRNG	-0,59681778	8,32E-20
214379_at	HNRPDL	-0,59687127	8,26E-22
211328_x_at	HFE	-0,59692451	6,05E-20
222070_at	DND1	-0,59719079	1,25E-18
203541_s_at	KLF9	-0,59757983	2,00E-21
215439_x_at		-0,59765447	2,46E-19
202855_s_at	SLC16A3	-0,59781849	2,38E-06
220808_at	THEG	-0,59803669	4,22E-18
219111_s_at	DDX54	-0,59848044	1,01E-17
216716_at	ABO	-0,59849928	9,33E-22
222086_s_at	WNT6	-0,59857882	9,64E-21
209810_at	SFTPB	-0,59862672	4,63E-22
212496_s_at	KDM4B	-0,5987578	4,84E-18
213443_at	TRADD	-0,59877102	8,96E-21
209807_s_at	NFIX	-0,59887325	6,50E-07
215202_at	LOC91316	-0,59891542	2,60E-17
221855_at	SDHAF1	-0,59905882	3,61E-16
210769_at	CNGB1	-0,59906488	7,35E-20
204751_x_at	DSC2	-0,59907768	1,75E-05
216107_at	LOC100129503	-0,5992291	2,14E-20

215203_at	GOLGA4	-0,59932153	2,83E-10
202000_at	NDUFA6	-0,59936498	1,48E-19
220151_at	C19orf73	-0,59959533	1,90E-21
214798_at	ATP2C2	-0,5996676	2,59E-21
203604_at	ZNF516	-0,59969486	4,43E-11
201408_at	PPP1CB	-0,59976587	1,73E-06
208274_at	OCLM	-0,60003361	1,22E-21
209136_s_at	USP10	-0,60042608	1,15E-13
206128_at	ADRA2C	-0,60059803	1,58E-20
204041_at	MAOB	-0,60071645	4,33E-23
214386_at		-0,6008856	2,47E-24
207001_x_at	TSC22D3	-0,60090062	2,86E-07
213406_at	WSB1	-0,60090928	2,84E-08
201441_at	COX6B1	-0,60095153	1,86E-13
217521_at		-0,60095402	1,05E-10
219928_s_at	CABYR	-0,60140702	2,75E-19
208300_at	PTPRH	-0,6016152	3,59E-23
219081_at	ANKHD1	-0,60166278	1,26E-23
221347_at	CHRM5	-0,60178389	8,28E-21
221377_s_at	RBPJL	-0,60178535	1,92E-21
201070_x_at	SF3B1	-0,60202927	3,08E-22
205605_at	HOXD9	-0,60280777	2,83E-21
210158_at	ERCC4	-0,60283847	1,06E-16
218011_at	UBL5	-0,60285279	5,74E-08
219796_s_at	CDHR5	-0,60308647	3,24E-19
212360_at	AMPD2	-0,60319408	1,96E-11
202896_s_at	SIRPA	-0,60323816	4,25E-13
202972_s_at	FAM13A	-0,60331056	5,10E-15
201237_at	CAPZA2	-0,60355612	0,000130277
78330_at	ZNF335	-0,60362911	5,98E-23
205784_x_at	ARVCF	-0,60371479	1,06E-15
209845_at	MKRN1	-0,60375024	3,32E-07
205362_s_at	PFDN4	-0,60375749	4,09E-24
221242_at		-0,60379443	1,46E-16
221155_x_at		-0,60393362	1,88E-15
51228_at	RBM12B	-0,60406251	5,51E-10
204786_s_at	IFNAR2	-0,60409925	9,80E-09
206325_at	SERPINA6	-0,60420647	7,50E-21
201693_s_at	EGR1	-0,60431485	6,63E-16
203193_at	ESRRA	-0,60478337	1,33E-19
203084_at	TGFB1	-0,6048669	5,85E-17
214283_at	TMEM97	-0,60490517	9,05E-21
208212_s_at	ALK	-0,60498105	2,55E-19
207674_at	FCAR	-0,60517598	0,000154239
221544_s_at	MED16	-0,60529155	1,50E-20
215397_x_at		-0,60551102	5,57E-19
208022_s_at	CDC14B	-0,60560778	1,16E-10
217025_s_at	DBN1	-0,60565182	2,00E-17
216572_at	FOXL1	-0,60570067	2,75E-22
222093_s_at	INO80B	-0,60604162	1,11E-14

203756_at	ARHGEF17	-0,60612985	3,12E-16
222252_x_at	UBQLN4	-0,60629784	1,69E-14
210036_s_at	KCNH2	-0,60646921	2,98E-07
207752_x_at	PRB1	-0,60680218	1,64E-13
205050_s_at	MAPK8IP2	-0,60681997	2,73E-19
214040_s_at	GSN	-0,60741157	7,62E-11
214145_s_at	SPTB	-0,60749516	3,91E-19
214310_s_at	ZFPL1	-0,60777244	2,54E-15
208451_s_at	C4A /// C4B /// LOC100509001	-0,60782433	0,000661269
222265_at	TNS4	-0,60803507	1,20E-18
215180_at		-0,60828197	1,05E-20
216245_at	IL1RN	-0,60835591	1,52E-15
203670_at	TTLL3	-0,60835919	4,75E-19
41856_at	UNC5B	-0,6085705	3,50E-18
213265_at	PGA3 /// PGA4 /// PGA5	-0,60895016	1,46E-13
222267_at	TMEM209	-0,60911865	9,60E-20
202032_s_at	MAN2A2	-0,60919699	3,34E-16
200770_s_at	LAMC1	-0,60923466	2,02E-22
219161_s_at	CKLF	-0,60976729	1,26E-07
209931_s_at	FKBP1B /// MFSD2B	-0,61031733	3,90E-11
213286_at	ZFR	-0,61039972	1,97E-19
219609_at	WDR25	-0,61053403	5,11E-21
214075_at	NENF	-0,61084485	2,67E-19
216646_at	DSCC1	-0,61090737	8,99E-19
212575_at	C19orf6	-0,61093797	4,71E-15
209410_s_at	GRB10	-0,61114641	4,64E-07
208376_at	CCR4	-0,61118237	6,29E-21
213681_at	CYHR1	-0,61133858	2,00E-20
210723_x_at		-0,61143809	3,26E-17
212478_at	RMND5A	-0,61158092	1,41E-09
206257_at	CCDC9	-0,61181195	1,13E-17
210210_at	MPZL1	-0,61184025	9,96E-09
210243_s_at	B4GALT3	-0,61200053	1,96E-10
218445_at	H2AFY2	-0,61200687	7,06E-17
217689_at	PTPN1	-0,61203513	1,84E-20
179_at	PMS2P11	-0,61235115	1,38E-16
213350_at	RPS11	-0,61262892	7,17E-12
210999_s_at	GRB10	-0,61273247	3,52E-05
201629_s_at	ACP1	-0,61295501	1,73E-07
211312_s_at	WISP1	-0,61296101	1,43E-18
211180_x_at	RUNX1	-0,6129819	1,56E-13
220279_at	TRIM17	-0,61333567	1,02E-17
204257_at	FADS3	-0,61334122	3,79E-18
219824_at	SLC13A4	-0,61338758	5,67E-22
210123_s_at	CHRFAM7A /// CHRNA7	-0,61352325	3,84E-16
221829_s_at	TNPO1	-0,61353169	2,50E-13
216928_at	TAL1	-0,61353545	1,21E-21
209961_s_at	HGF	-0,61359258	4,51E-18
203524_s_at	MPST	-0,61404345	7,91E-21
207462_at	GLRA2	-0,61429707	8,91E-21

211512_s_at	OGFR	-0,61439237	1,22E-12
204416_x_at	APOC1	-0,61464149	2,24E-20
212155_at	RNF187	-0,61468589	6,07E-11
209070_s_at	RGS5	-0,61476444	1,60E-19
213195_at	C17orf108	-0,61488153	4,74E-19
212537_x_at	RPL17	-0,61491582	2,45E-06
201864_at	GDI1	-0,61491729	1,14E-20
222113_s_at	EPS15L1	-0,61502648	9,03E-14
221136_at	GDF2	-0,61513172	1,33E-18
205919_at	HBE1	-0,61517978	2,38E-13
216181_at	SYNJ2	-0,61553367	1,12E-17
220111_s_at	ANO2	-0,61563084	2,28E-24
215712_s_at	IGFALS	-0,61581131	1,26E-18
217182_at	MUC5AC	-0,6160982	1,92E-18
207069_s_at	SMAD6	-0,61629441	9,97E-23
204316_at	RGS10	-0,61648057	6,07E-16
217681_at	LOC100289775 /// WNT7B	-0,61652152	2,92E-21
220263_at	SMAD5OS	-0,6165745	2,08E-16
202209_at	LSM3	-0,61674174	8,01E-15
212859_x_at	MT1E	-0,61691726	1,31E-06
212073_at	CSNK2A1	-0,61707671	5,15E-20
217855_x_at	SDF4	-0,61711283	2,90E-18
205561_at	KCTD17	-0,61733251	1,18E-16
203422_at	POLD1	-0,6173375	3,76E-15
203814_s_at	NQO2	-0,61744859	1,84E-08
205756_s_at	F8	-0,61764382	3,10E-10
208351_s_at	MAPK1	-0,61775108	4,41E-19
221112_at	IL1RAPL2	-0,61801608	3,95E-21
206108_s_at	SRSF6	-0,6184704	9,87E-06
210228_at	CSF2	-0,61898882	9,25E-21
205370_x_at	DBT	-0,61925166	8,58E-12
210938_at	PDX1	-0,61937988	3,05E-22
208353_x_at	ANK1	-0,61950992	6,60E-05
209473_at	ENTPD1	-0,6198069	5,25E-12
211500_at	MAPK11	-0,61985088	1,31E-18
202588_at	AK1	-0,61986943	1,19E-17
210850_s_at	ELK1	-0,62021082	3,95E-15
216647_at	TCF3	-0,62075587	1,74E-20
55093_at	CHPF2	-0,62079263	1,40E-24
216689_x_at	ARHGAP1	-0,62080629	8,81E-14
220766_at	BTG4	-0,62085996	9,92E-23
203161_s_at	RNF8	-0,62090296	2,73E-21
208904_s_at	RPS28	-0,62109072	1,92E-09
206130_s_at	ASGR2	-0,62120642	9,25E-09
209413_at	B4GALT2	-0,62121275	1,40E-19
206539_s_at	CYP4F12	-0,62126733	4,02E-21
210726_at	CYP3A4	-0,62130245	1,20E-22
215110_at	MBL1P	-0,62132461	2,80E-17
203842_s_at	MAPRE3	-0,62170426	3,20E-18
208286_x_at	5F1 /// POU5F1B /// POU5F1P3 /// POU5	-0,62170775	3,43E-19

218634_at	PHLDA3	-0,62170894	8,67E-20
205602_x_at	PSG7	-0,62187142	1,87E-17
214740_at	POLR2J /// POLR2J2 /// POLR2J3	-0,62191621	3,66E-14
220923_s_at	PNMA3	-0,62204797	2,81E-17
209518_at	SMARCD1	-0,62237943	8,11E-15
219127_at	PRR15L	-0,62263304	2,85E-17
209543_s_at	CD34	-0,62273628	1,42E-18
37005_at	NBL1	-0,62297415	5,42E-17
212141_at	MCM4	-0,62299753	2,84E-15
210519_s_at	NQO1	-0,62319983	2,98E-16
211831_s_at	THPO	-0,62322429	6,99E-19
216975_x_at	NPAS1	-0,62322836	4,05E-19
219022_at	C12orf43	-0,62325876	2,84E-11
207302_at	SGCG	-0,62341006	1,88E-22
215741_x_at	AKAP8L	-0,62349089	7,45E-18
209747_at	TGFB3	-0,62407347	8,66E-18
218117_at	RBX1	-0,62430887	2,20E-05
204903_x_at	ATG4B	-0,62451124	2,37E-21
207805_s_at	PSMD9	-0,62457286	1,27E-21
215881_x_at	SSX2 /// SSX2B /// SSX3	-0,62462999	1,40E-17
205379_at	CBR3	-0,62468062	3,57E-14
208484_at	HIST1H1A	-0,62484546	1,08E-18
217007_s_at	ADAM15	-0,62500627	4,07E-15
202700_s_at	TMEM63A	-0,62501089	1,70E-15
208430_s_at	DTNA	-0,62503665	3,36E-23
215130_s_at	IQCK	-0,62507513	1,65E-17
218213_s_at	C11orf10	-0,62538985	9,31E-08
201206_s_at	RRBP1	-0,62559837	6,68E-15
221032_s_at	TMPRSS5	-0,62561496	1,14E-17
203101_s_at	MGAT2	-0,62573685	1,23E-19
201108_s_at	THBS1	-0,62575442	7,32E-08
205184_at	GNG4	-0,62610844	9,07E-21
211994_at	WNK1	-0,62614774	3,20E-09
221710_x_at	FAM176B	-0,62624352	1,31E-16
214618_at	CFLAR	-0,62632697	1,11E-09
210597_x_at	PRB1	-0,62648698	3,23E-14
202236_s_at	SLC16A1	-0,62658415	1,07E-15
208730_x_at	RAB2A	-0,62661888	1,13E-16
203777_s_at	RPS6KB2	-0,62673747	1,22E-17
213152_s_at	SRSF8	-0,62677753	3,16E-09
214076_at	GFOD2	-0,62685832	1,71E-23
207021_at	ZPBP	-0,62720473	2,09E-19
212271_at	MAPK1	-0,62742512	8,44E-13
208532_x_at	KRTAP5-8	-0,62746338	5,74E-16
216890_at		-0,62748296	5,31E-21
202507_s_at	SNAP25	-0,62752749	1,32E-17
216768_x_at	TTC38	-0,62783431	8,17E-21
212524_x_at	H2AFX	-0,62791586	2,07E-22
211658_at	PRDX2	-0,62792667	7,42E-09
213812_s_at	CAMKK2	-0,6279906	1,30E-09

205265_s_at	SPEG	-0,62801358	6,10E-19
209561_at	THBS3	-0,62805264	2,76E-23
215418_at	PARVA	-0,62862216	8,26E-20
211587_x_at	CHRNA3	-0,62909713	4,92E-19
216183_at	TGM2	-0,62911694	8,51E-18
207235_s_at	GRM5	-0,62923341	1,46E-24
214444_s_at	PVR	-0,62988026	1,49E-21
222346_at	LAMA1	-0,63016779	4,37E-23
208176_at	DUX1	-0,63018082	1,44E-16
218665_at	FZD4	-0,63066447	1,74E-18
215682_at	LOC440792	-0,63071198	1,87E-20
212829_at	PIP4K2A	-0,6309198	1,57E-10
221537_at	PLXNA1	-0,63121868	5,91E-17
210341_at	MYT1	-0,63133747	2,58E-24
210529_s_at	FAM115A	-0,63170343	4,16E-17
208864_s_at	TXN	-0,63174652	2,96E-06
214074_s_at	CTTN	-0,6320052	3,04E-16
217414_x_at	HBA1 /// HBA2	-0,63212483	1,83E-09
206280_at	CDH18	-0,63214994	1,28E-21
215264_at	EMX1	-0,63223985	4,13E-24
208492_at	RFXAP	-0,63238431	7,35E-20
202267_at	LAMC2	-0,63245398	2,11E-22
208189_s_at	MYO7A	-0,63256052	6,86E-18
217710_x_at	ITPK1	-0,63260345	5,96E-19
203174_s_at	ARFRP1	-0,63263644	7,24E-16
206179_s_at	TPPP	-0,63283776	9,36E-20
220665_at	LUZP4	-0,63299655	4,72E-21
217486_s_at	ZDHHC17	-0,633032	4,61E-18
210858_x_at	ATM	-0,63321146	4,93E-12
217570_x_at		-0,63332612	3,75E-20
219221_at	ZBTB38	-0,63343231	3,23E-10
214004_s_at	VGLL4	-0,63360993	1,60E-12
202828_s_at	MMP14	-0,63361245	5,28E-16
209365_s_at	ECM1	-0,63385395	2,62E-18
202009_at	TWF2	-0,63391378	4,66E-20
201195_s_at	SLC7A5	-0,63403564	1,28E-05
212793_at	DAAM2	-0,63406988	0,000160737
213363_at	CA5BP	-0,63411135	4,15E-15
207721_x_at	HINT1	-0,63422053	2,91E-09
204904_at	GJA4	-0,63447742	3,24E-20
221338_at	LY6G6E	-0,63459672	4,54E-21
217160_at	TSPY1	-0,63466197	3,03E-20
205391_x_at	ANK1	-0,63486923	1,65E-05
202178_at	PRKCZ	-0,63499228	2,27E-15
207492_at	NGLY1	-0,63505521	3,42E-13
207778_at	REG1P	-0,6350643	6,01E-23
208881_x_at	IDI1	-0,63529855	4,16E-05
220956_s_at	EGLN2	-0,63536556	6,87E-18
207576_x_at	OXT	-0,63541637	1,53E-18
204229_at	SLC17A7	-0,6356463	1,77E-15

216200_at	PLEKHM1	-0,63570566	5,95E-20
217137_x_at		-0,6359009	9,52E-11
205632_s_at	PIP5K1B	-0,63590122	5,11E-05
202574_s_at	CSNK1G2	-0,6359228	2,37E-14
212620_at	ZNF609	-0,63593028	4,77E-17
218778_x_at	EPS8L1	-0,63609486	4,20E-23
219051_x_at	METRN	-0,63645531	5,62E-15
207933_at	ZP2	-0,63682504	1,29E-20
218745_x_at	TMEM161A	-0,63688632	2,66E-16
205177_at	TNNI1	-0,63689672	1,68E-20
219102_at	RCN3	-0,63724883	9,29E-21
207601_at	SULT1B1	-0,63761673	5,21E-10
217756_x_at	SERF2	-0,63781872	1,81E-15
215484_at		-0,63794057	2,64E-19
213442_x_at	SPDEF	-0,6379989	1,10E-18
219438_at	NKAIN1	-0,63818443	1,29E-21
203800_s_at	MRPS14	-0,63818607	2,54E-16
201730_s_at	TPR	-0,63820531	1,37E-16
200032_s_at	RPL9	-0,63845496	0,000152739
220491_at	HAMP	-0,63852775	2,26E-18
214197_s_at	SETDB1	-0,63860038	9,61E-22
217535_at	FAM49B	-0,63861211	2,38E-24
211915_s_at	TUBB4Q	-0,63866195	1,24E-20
212332_at	RBL2	-0,63932227	2,89E-14
216081_at	LAMA4	-0,63937218	1,60E-23
204738_s_at	KRIT1	-0,63954951	3,90E-18
209234_at	KIF1B	-0,63959989	1,13E-07
206821_x_at	AGFG2	-0,63960083	3,00E-18
217271_at	GNA11	-0,63961425	2,95E-21
221370_at	LOC100287163 /// ZNF717 /// ZNF73	-0,63975123	1,30E-21
209772_s_at	CD24	-0,64025307	3,88E-13
202175_at	CHPF	-0,64040777	5,67E-18
214941_s_at	PRPF40A	-0,64042132	8,65E-17
206521_s_at	GTF2A1	-0,64050261	1,24E-21
207573_x_at	ATP5L	-0,64051392	1,29E-12
203059_s_at	PAPSS2	-0,64076492	3,22E-21
219434_at	TREM1	-0,64082283	3,05E-08
221663_x_at	HRH3	-0,64084573	3,47E-18
210977_s_at	HSF4	-0,64088996	7,49E-16
208609_s_at	TNXB	-0,64164955	8,59E-16
201910_at	FARP1	-0,64171571	5,66E-21
222039_at	KIF18B	-0,6419273	1,10E-15
208050_s_at	CASP2	-0,64193473	2,62E-12
208145_at		-0,64200592	5,37E-21
208090_s_at	AIRE	-0,6420327	4,55E-19
221180_at	YSK4	-0,64205193	3,60E-18
205199_at	CA9	-0,64219716	4,80E-20
201152_s_at	MBNL1	-0,64233656	5,61E-08
217010_s_at	CDC25C	-0,64246201	1,23E-20
221659_s_at	MYL10	-0,64256665	1,99E-21

220951_s_at	A1CF	-0,64281832	4,83E-21
213091_at	CRTC1	-0,64283985	7,90E-17
214869_x_at	GAPVD1	-0,64288449	3,49E-21
218952_at	PCSK1N	-0,6433676	6,36E-07
64942_at	GPR153	-0,64353811	2,23E-18
206586_at	CNR2	-0,6435774	3,40E-18
204188_s_at	RARG	-0,64363984	1,95E-15
217930_s_at	TOLLIP	-0,64375499	2,07E-13
217046_s_at	AGER	-0,64381732	1,53E-23
202718_at	IGFBP2	-0,64387369	2,47E-12
212479_s_at	RMND5A	-0,64440512	2,74E-08
210008_s_at	MRPS12	-0,64488303	3,51E-19
207907_at	TNFSF14	-0,64502817	1,99E-12
210025_s_at	CARD10	-0,64509219	8,49E-18
211468_s_at	RECQL5	-0,64525822	2,76E-22
219811_at	DGCR8	-0,64535099	4,65E-17
206820_at	AGFG2	-0,64540246	5,64E-21
213201_s_at	TNNT1	-0,64561294	1,67E-09
51774_s_at	UBE2D4	-0,64583871	9,62E-15
201149_s_at	TIMP3	-0,6458727	1,23E-23
208584_at	SNCG	-0,6458751	7,47E-16
201392_s_at	IGF2R	-0,64587711	5,36E-11
212736_at	C16orf45	-0,64590261	1,61E-22
216367_at	COL4A3	-0,64591938	2,05E-21
206892_at	AMHR2	-0,6459801	1,18E-15
204996_s_at	CDK5R1	-0,64631832	4,27E-20
211125_x_at	GRIN1	-0,64671147	1,56E-17
202652_at	APBB1	-0,64692084	1,79E-12
221857_s_at	TJAP1	-0,64692599	2,36E-17
201208_s_at	TNFAIP1	-0,64694122	1,53E-18
220827_at		-0,64724909	6,04E-21
213651_at	INPP5J	-0,6472976	4,96E-20
214924_s_at	TRAK1	-0,64741143	3,00E-15
211973_at	LOC100507620	-0,64748408	6,28E-11
207353_s_at	HMX1	-0,64789297	8,42E-18
211647_x_at	IGHG1 /// IGHM /// LOC100133862	-0,64802551	1,20E-16
208583_x_at	HIST1H2AJ	-0,64829419	1,03E-15
208000_at	GML	-0,64844609	3,47E-23
212213_x_at	OPA1	-0,64844857	5,54E-10
207488_at		-0,64859	7,27E-23
210955_at	CASP10	-0,64868873	1,44E-16
210638_s_at	FBXO9	-0,64868976	7,00E-09
215338_s_at	NKTR	-0,64888985	6,08E-09
208238_x_at		-0,6489857	9,51E-14
207669_at	KRT83	-0,64911688	1,43E-20
214206_at	PPIL6	-0,64950221	5,54E-20
211469_s_at	CXCR6	-0,64955679	3,17E-12
207679_at	PAX3	-0,64960798	4,72E-21
60528_at	JMJD7-PLA2G4B /// PLA2G4B	-0,64988993	4,93E-14
205847_at	PRSS22	-0,64991886	1,10E-18

214990_at	PIGO	-0,6503805	4,36E-16
217704_x_at	SUZ12P	-0,65040815	3,48E-17
205318_at	KIF5A	-0,65060436	3,41E-18
217944_at	POMGNT1	-0,65074333	1,64E-12
201644_at	TSTA3	-0,65075981	5,84E-07
212813_at	JAM3	-0,65090548	2,97E-09
212012_at	PXDN	-0,65129605	1,36E-21
200999_s_at	CKAP4	-0,65160577	3,62E-10
213768_s_at	ASCL1	-0,65163054	1,50E-18
214903_at	SYT2	-0,6520799	9,28E-17
210402_at	KCNJ1	-0,6520901	2,90E-21
219064_at	ITIH5	-0,6521659	2,42E-24
211531_x_at	PRB1	-0,65216611	1,71E-13
216772_at		-0,65241957	3,66E-23
216186_at		-0,65251429	1,01E-18
217415_at	POLR2A	-0,65260751	4,80E-24
221968_s_at	ZNF771	-0,65285181	1,50E-22
215490_at	C1orf69	-0,65286553	3,70E-20
212270_x_at	RPL17	-0,65324404	5,66E-07
221390_s_at	MTMR8	-0,65329508	7,50E-21
205608_s_at	ANGPT1	-0,65363663	1,53E-21
204345_at	COL16A1	-0,65385241	6,16E-24
217783_s_at	YPEL5	-0,65385709	1,11E-09
206477_s_at	NOVA2	-0,65407049	3,50E-21
202974_at	MPP1	-0,65432196	3,09E-09
206907_at	TNFSF9	-0,65450427	4,43E-25
200977_s_at	TAX1BP1	-0,65453627	3,29E-06
218258_at	POLR1D	-0,65456372	3,70E-09
207233_s_at	MITF	-0,65467315	3,09E-21
210182_at	CORT	-0,65474194	3,65E-21
210827_s_at	ELF3	-0,65474499	1,00E-20
216293_at	CLTA	-0,65481191	2,44E-20
213693_s_at	MUC1	-0,65485605	1,40E-16
205012_s_at	HAGH	-0,65493546	6,45E-07
204008_at	DNAL4	-0,65510711	4,64E-17
201423_s_at	CUL4A	-0,65515333	1,43E-11
202294_at	STAG1	-0,65546382	1,87E-16
213575_at	TRA2A	-0,65553952	3,04E-09
221074_at	NCR2	-0,65586026	1,90E-19
202607_at	NDST1	-0,65608493	2,65E-15
214305_s_at	SF3B1	-0,65629279	6,91E-19
206196_s_at	RUNDC3A	-0,65672881	0,00056884
218200_s_at	NDUFB2	-0,65684653	2,96E-09
207105_s_at	PIK3R2	-0,65697571	5,53E-19
220583_at		-0,65698102	1,19E-20
221484_at	B4GALT5	-0,65701454	2,79E-07
206904_at	MATN1	-0,6571168	7,82E-21
214815_at	TRIM33	-0,65728503	1,05E-12
211259_s_at	BMP7	-0,65740697	6,93E-23
204971_at	CSTA	-0,65779215	7,48E-05

214399_s_at	KRT4	-0,65786652	8,55E-22
214832_at	HNF4A	-0,65792452	5,59E-20
202770_s_at	CCNG2	-0,65799205	8,36E-06
204419_x_at	HBG1 /// HBG2	-0,65814204	0,001126852
201580_s_at	TMX4	-0,65820166	7,21E-16
212134_at	PHLDB1	-0,65845753	1,89E-20
206424_at	CYP26A1	-0,65874894	4,01E-22
214024_s_at	DGCR6L	-0,65884847	1,85E-22
201533_at	CTNNB1	-0,65891557	4,85E-11
206178_at	PLA2G5	-0,65910709	3,90E-21
215407_s_at	ASTN2	-0,65911042	3,42E-17
210200_at	WWP2	-0,65936088	1,37E-23
214085_x_at	GLIPR1	-0,65954029	1,41E-12
204815_s_at	DHX34	-0,6596086	1,28E-16
206778_at	CRYBB2	-0,65980186	6,44E-18
219341_at	CLN8	-0,65981855	1,35E-18
208328_s_at	MEF2A	-0,65989715	6,89E-08
222338_x_at		-0,66032814	3,74E-12
217489_s_at	IL6R	-0,66034691	3,04E-12
208785_s_at	MAP1LC3B	-0,66041023	2,83E-15
206857_s_at	FKBP1B	-0,66057564	1,07E-05
201941_at	CPD	-0,66078631	8,09E-05
220650_s_at	SLC9A5	-0,6609942	1,58E-20
217377_x_at	NTRK3	-0,66111959	2,41E-19
218985_at	SLC2A8	-0,66136119	1,03E-17
202048_s_at	CBX6	-0,66175203	4,75E-18
221281_at	SRC	-0,66175737	1,39E-18
214607_at	PAK3	-0,662294	2,18E-19
201125_s_at	ITGB5	-0,66277836	3,52E-11
209513_s_at	HSDL2	-0,66300626	1,03E-09
214701_s_at	FN1	-0,66363115	7,64E-24
218000_s_at	PHLDA1	-0,66397844	5,71E-22
205095_s_at	ATP6V0A1	-0,66415297	7,07E-17
221141_x_at	EPN1	-0,66473385	1,12E-17
202192_s_at	GAS7	-0,66475344	2,21E-12
207468_s_at	SFRP5	-0,66492491	1,23E-18
208034_s_at	PROZ	-0,6651346	1,53E-18
200696_s_at	GSN	-0,66528228	2,15E-13
220632_s_at	POMT2	-0,66539848	1,34E-19
217329_x_at		-0,66560206	1,74E-14
220740_s_at	SLC12A6	-0,66566814	1,19E-14
207743_at	LOC100505593	-0,66594754	6,22E-21
204983_s_at	GPC4	-0,66607763	3,10E-19
204848_x_at	HBG1 /// HBG2	-0,66619031	0,000668536
217700_at	CNPY4	-0,66645775	4,35E-19
204090_at	STK19	-0,66654329	7,83E-20
213422_s_at	MXRA8	-0,66668358	2,44E-17
211037_s_at	MBOAT7	-0,6672879	1,09E-15
209274_s_at	ISCA1	-0,66763926	1,65E-05
210808_s_at	NOX1	-0,66819917	2,95E-24

211263_s_at	PCSK6	-0,66837046	9,75E-19
203421_at	TP53I11	-0,66838299	3,14E-14
201083_s_at	BCLAF1	-0,6685757	1,80E-09
215556_at		-0,66884958	1,07E-22
211486_s_at	KCNQ2	-0,66907622	3,75E-21
212899_at	CDK19	-0,6690886	2,83E-20
202072_at	HNRNPL	-0,66927647	1,40E-13
208417_at	FGF6	-0,66930369	1,52E-18
202949_s_at	FHL2	-0,67005181	2,13E-06
218406_x_at	NENF	-0,67017847	7,96E-21
58900_at	UBE2D4	-0,67034119	1,76E-16
214213_x_at	LMNA	-0,67057522	5,04E-16
207482_at	TP53TG5	-0,67116711	7,90E-21
216011_at	SLC39A9	-0,67122952	2,70E-21
211839_s_at	CSF1	-0,67152282	1,25E-17
214311_at	ZFPL1	-0,67162102	2,90E-20
216241_s_at	TCEA1	-0,67177251	2,79E-11
208950_s_at	ALDH7A1	-0,67178746	1,71E-23
209886_s_at	SMAD6	-0,67189894	2,88E-23
218033_s_at	SNN	-0,67230983	2,82E-17
205494_at	ZNF821	-0,67237573	1,77E-19
216583_x_at		-0,67244932	7,08E-17
220823_at	LOC729164	-0,67259255	1,15E-22
209836_x_at	BOLA2 /// BOLA2B	-0,67262225	1,40E-10
208667_s_at	ST13	-0,67275998	7,62E-07
217269_s_at	TMPRSS15	-0,67296856	2,45E-21
219114_at	C3orf18	-0,67374674	4,84E-17
208043_at		-0,67386815	9,28E-25
221166_at	FGF23	-0,67409012	3,27E-20
214795_at	ZMYND8	-0,67458991	1,07E-19
206149_at	CHP2	-0,67466063	4,33E-24
216842_x_at	BMY1B /// RBMY1D /// RBMY1E /// RBM	-0,674764	1,31E-18
35626_at	SGSH	-0,67486209	8,02E-16
221317_x_at	PCDHB6	-0,67495179	2,39E-21
209409_at	GRB10	-0,67501703	1,88E-05
204306_s_at	CD151	-0,67505613	3,08E-12
214517_at	KRTAP5-9	-0,67520496	3,30E-22
211731_x_at	SSX3	-0,67524577	3,09E-21
220714_at	PRDM14	-0,67534708	3,80E-21
221367_at	MOS	-0,67550827	8,06E-24
217033_x_at	NTRK3	-0,67553322	4,85E-20
214019_at		-0,6755896	2,72E-21
216418_at	ABCD1	-0,67588292	3,51E-21
208632_at	RNF10	-0,6758959	1,37E-07
222015_at	CSNK1E	-0,67593755	5,05E-18
215278_at		-0,67602736	5,11E-25
211498_s_at	NKX3-1	-0,67618635	4,19E-22
204945_at	PTPRN	-0,67646287	7,43E-21
207977_s_at	DPT	-0,67648681	6,38E-20
39854_r_at	PNPLA2	-0,67662157	7,74E-17

219893_at	CCDC71	-0,67668894	1,27E-16
208390_s_at	GLP1R	-0,67676832	3,12E-16
215826_x_at	ZNF835	-0,67688107	2,77E-15
205156_s_at	ACCN2	-0,67699232	3,24E-19
219382_at	SERTAD3	-0,67732706	1,92E-13
221429_x_at	TEX13A	-0,67738076	7,03E-21
202940_at	WNK1	-0,67760512	4,28E-15
203794_at	CDC42BPA	-0,6777578	7,98E-23
203628_at	IGF1R	-0,67785902	2,48E-08
221939_at	YIPF2	-0,67786116	1,07E-15
206419_at	RORC	-0,67818577	1,84E-19
216284_at		-0,67849574	5,25E-24
205559_s_at	PCSK5	-0,67883753	4,87E-10
205914_s_at	GRIN1	-0,678838	3,98E-13
209848_s_at	SILV	-0,6788763	2,95E-22
213871_s_at	C6orf108	-0,67928871	3,50E-18
211040_x_at	GTSE1	-0,67951806	3,20E-18
204603_at	EXO1	-0,67962146	4,21E-18
215145_s_at	CNTNAP2	-0,67967775	4,74E-20
216051_x_at		-0,67987255	4,62E-18
222090_at	LOC100134713	-0,67994956	7,35E-19
220508_at	CCT8L2	-0,67997545	5,89E-21
206470_at	PLXNC1	-0,68000363	1,03E-09
208041_at	GRK1	-0,68008001	2,30E-22
218744_s_at	PACSIN3	-0,68014358	3,70E-20
218034_at	FIS1	-0,68027091	5,69E-10
202276_at	SHFM1	-0,68030031	3,38E-14
38710_at	OTUB1	-0,68036009	5,80E-15
210469_at	DLG5	-0,68037203	7,19E-19
216434_at	TTC38	-0,68041989	1,10E-20
221812_at	FBXO42	-0,68051209	6,80E-20
213608_s_at	SRRD	-0,68051404	7,72E-06
206209_s_at	CA4	-0,68072943	3,82E-05
205272_s_at	PRH1 /// PRH2	-0,68098799	8,81E-19
210419_at	BARX2	-0,68101259	5,60E-21
210300_at	REM1	-0,68105094	7,98E-18
203918_at	PCDH1	-0,68110564	2,46E-18
212585_at	OSBPL8	-0,68116254	0,002288392
207190_at	ZZEF1	-0,68181849	4,51E-23
209981_at	CSDC2	-0,68213604	2,33E-17
213274_s_at	CTSB	-0,68254928	7,37E-12
209301_at	CA2	-0,68268691	3,93E-05
206705_at	TULP1	-0,68321276	2,96E-23
217703_x_at		-0,68328216	1,22E-16
215834_x_at	SCARB1	-0,68333639	4,03E-21
218368_s_at	TNFRSF12A	-0,68334935	1,32E-21
219704_at	YBX2	-0,68342619	1,57E-16
202327_s_at	PKD1	-0,68365485	7,11E-22
210795_s_at	MEG3	-0,68421489	1,96E-16
210762_s_at	DLC1	-0,68432892	5,80E-10

216505_x_at		-0,6843717	2,23E-14
219914_at	ECEL1	-0,68441369	8,83E-20
212430_at	RBM38	-0,6851736	5,27E-06
221737_at	GNA12	-0,6852718	1,08E-16
210553_x_at	PCSK6	-0,68527392	1,50E-19
204763_s_at	GNAO1	-0,68557558	4,49E-18
211098_x_at	TMCO1	-0,68606349	2,47E-19
212007_at	UBXN4	-0,68696102	3,87E-19
38707_r_at	E2F4	-0,68697479	7,83E-18
220233_at	FBXO17 /// SARS2	-0,68705441	1,93E-19
200026_at	RPL34	-0,68708205	4,01E-05
208347_at		-0,68742633	2,10E-19
210215_at	TFR2	-0,68815761	3,76E-07
214151_s_at	CCPG1	-0,68828326	4,84E-09
203904_x_at	CD82	-0,68915638	6,15E-14
202389_s_at	HTT	-0,68930311	1,42E-20
210586_x_at	RHD	-0,68956444	6,76E-07
201712_s_at	RANBP2	-0,68965961	6,68E-19
215588_x_at	RIOK3	-0,68971845	4,99E-13
213181_s_at	MOCS1	-0,69014463	3,34E-17
203679_at	TMED1	-0,69111927	3,25E-19
219057_at	RABEP2	-0,69112154	2,35E-17
200095_x_at	RPS10	-0,69118085	1,09E-13
211926_s_at	MYH9	-0,69120083	8,93E-17
210798_x_at	MASP2	-0,69122209	4,20E-23
214498_at	ASIP	-0,69128237	3,77E-20
217283_at		-0,69212794	2,15E-23
209957_s_at	NPPA	-0,6927478	9,37E-19
210392_x_at	NR6A1	-0,69281934	1,11E-23
211140_s_at	CASP2	-0,69283324	4,52E-23
211360_s_at	ITPR2	-0,69295542	8,05E-24
219610_at	RGNEF	-0,6931159	5,09E-20
217058_at	GNAS	-0,69364359	5,91E-21
205058_at	SLC26A1	-0,69381126	1,76E-21
221048_x_at	C17orf80	-0,69394996	2,10E-23
215886_x_at	USP12	-0,69408623	7,28E-20
214174_s_at	PDLIM4	-0,69479789	1,26E-20
207312_at	PHKG1	-0,69495733	9,23E-17
210524_x_at		-0,69578896	1,70E-09
201123_s_at	EIF5A	-0,69582514	6,17E-05
209046_s_at	GABARAPL2	-0,69604342	2,74E-17
207491_at	MOGAT2	-0,69651907	1,48E-22
208482_at	SSTR1	-0,69652559	1,71E-20
220427_at	OBSCN	-0,69653002	3,21E-18
213687_s_at	RPL35A	-0,69657827	1,77E-11
219249_s_at	FKBP10	-0,69674133	1,08E-22
213085_s_at	WWC1	-0,69682475	3,50E-21
214133_at	MUC6	-0,69684698	1,36E-22
202879_s_at	CYTH1	-0,69735589	2,25E-11
203966_s_at	PPM1A	-0,69784342	1,75E-09

221947_at		-0,69835459	9,66E-23
207745_at	CABP2	-0,69839874	1,73E-23
59437_at	C9orf116	-0,69857532	3,29E-25
209661_at	KIFC3	-0,69858016	6,35E-18
211659_at	GPR135	-0,69898257	3,46E-22
204104_at	SNAPC2	-0,69908501	1,79E-16
208250_s_at	DMBT1	-0,69911029	1,39E-16
216943_at		-0,6991412	3,59E-20
209183_s_at	C10orf10	-0,69932428	1,32E-19
121_at	PAX8	-0,70056454	2,42E-17
208130_s_at	TBXAS1	-0,70076185	1,13E-17
217117_x_at	MUC3A	-0,70087379	2,49E-18
221789_x_at	RHOT2	-0,70101252	2,60E-20
215730_at		-0,70131981	1,19E-24
209660_at	TTR	-0,70167224	1,81E-20
221271_at	IL21	-0,70186859	1,74E-19
210422_x_at	SLC11A1	-0,70201846	1,05E-14
209405_s_at	FAM3A	-0,70222867	1,25E-18
210132_at	EFNA3	-0,70237139	2,99E-18
207612_at	WNT8B	-0,70284215	3,42E-20
207418_s_at	DDO	-0,70295249	3,26E-21
206208_at	CA4	-0,7032368	2,25E-07
213515_x_at	HBG1 /// HBG2	-0,70341205	0,000149522
207208_at	RBMXL2	-0,70372596	9,83E-24
206922_at	VCY /// VCY1B	-0,70379995	3,14E-25
217446_x_at		-0,70391421	1,87E-16
210724_at	EMR3	-0,70419361	2,42E-10
64440_at	IL17RC	-0,70447619	7,22E-19
212089_at	LMNA	-0,7046513	8,48E-18
201733_at	CLCN3	-0,70481791	9,18E-16
220105_at	RTDR1	-0,70500425	7,30E-21
206560_s_at	MIA	-0,70508135	2,65E-18
211554_s_at	APAF1	-0,70523836	1,77E-23
209372_x_at	TUBB2A /// TUBB2B	-0,70533237	1,03E-05
217544_at		-0,70549725	2,85E-15
217713_x_at		-0,70555156	9,69E-16
220339_s_at	TPSG1	-0,70562738	1,80E-19
201526_at	ARF5	-0,70565175	2,52E-19
219144_at	DUSP26	-0,70578859	1,57E-25
220288_at	MYO15A	-0,70597049	1,62E-20
215607_x_at		-0,70654428	1,10E-19
205102_at	TMPRSS2	-0,70661049	2,74E-18
221675_s_at	CHPT1	-0,70693101	1,78E-08
209050_s_at	RALGDS	-0,70714855	1,63E-08
201796_s_at	VARS	-0,70725761	3,58E-19
221946_at	C9orf116	-0,70727887	1,26E-23
215206_at		-0,70772196	2,59E-09
205321_at	EIF2S3	-0,7078099	6,17E-07
221966_at	GPR137	-0,70807425	4,70E-17
211673_s_at	MOCS1	-0,70809018	4,73E-21

213731_s_at	TCF3	-0,70819125	2,16E-21
209506_s_at	NR2F1	-0,70819821	2,61E-22
206691_s_at	PDIA2	-0,70820721	3,06E-19
220548_at	PKDREJ	-0,70833582	1,84E-22
215266_at	DNAH3	-0,70834105	1,86E-18
207005_s_at	BCL2	-0,70840533	1,09E-21
205734_s_at	AFF3	-0,70841085	1,73E-23
206612_at	CACNG1	-0,70852992	1,80E-20
202434_s_at	CYP1B1	-0,70877367	3,48E-12
205189_s_at	FANCC	-0,7088685	1,60E-17
49329_at	KLHL22	-0,70942217	2,88E-11
204259_at	MMP7	-0,70946111	7,64E-24
219554_at	RHCG	-0,70951648	8,79E-22
214080_x_at	PRKCSH	-0,70958403	6,47E-16
213863_s_at	OAZ3	-0,70981625	7,91E-22
215449_at	TSPO2	-0,71028483	2,84E-08
220544_at	TSKS	-0,71038285	6,80E-22
207684_at	TBX6	-0,71126329	4,46E-20
222323_at		-0,71143982	6,95E-19
204920_at	CPS1	-0,71170412	6,20E-22
212302_at	RTF1	-0,7117361	1,04E-20
204609_at	CCDC85B	-0,71181122	2,91E-22
203437_at	TMEM11	-0,71191997	8,62E-25
208141_s_at	DOHH	-0,71223917	1,17E-18
207289_at	MMP25	-0,71230321	3,14E-17
219558_at	ATP13A3	-0,7125429	4,67E-20
209698_at	CCHCR1	-0,71256378	5,83E-19
207028_at	MYCNOS	-0,71262188	7,52E-23
209018_s_at	PINK1	-0,71269077	3,16E-12
211667_x_at		-0,71273067	1,38E-16
213250_at	CCDC85B	-0,71285133	2,63E-24
215505_s_at	STRN3	-0,71334394	4,52E-15
205163_at	MYLPF	-0,71341523	2,49E-23
205738_s_at	FABP3	-0,71412648	3,99E-22
207530_s_at	CDKN2B	-0,71428652	8,19E-26
214158_s_at	PRDM10	-0,71444924	5,38E-23
219668_at	GDAP1L1	-0,7145992	3,70E-21
35150_at	CD40	-0,7146171	1,61E-14
218677_at	S100A14	-0,71476987	2,80E-22
206137_at	RIMS2	-0,71481164	3,65E-22
205601_s_at	HOXB5	-0,71494348	8,42E-21
221359_at	GDNF	-0,71516934	6,89E-25
213455_at	FAM114A1	-0,7154472	6,67E-25
204619_s_at	VCAN	-0,71559381	3,26E-07
222082_at	ZBTB7A	-0,71565548	9,08E-16
214380_at	PRPF31	-0,71578466	4,26E-22
211332_x_at	HFE	-0,715876	1,38E-20
218629_at	SMO	-0,71615213	1,56E-22
205465_x_at	HS3ST1	-0,71661368	1,49E-22
31846_at	RHOD	-0,71662279	1,54E-17

207442_at	CSF3	-0,71673544	1,26E-21
220633_s_at	HP1BP3	-0,71684876	4,59E-23
216019_x_at	PHLDB1	-0,71733425	2,49E-18
219852_s_at	MORN1	-0,71768143	6,89E-20
208260_at	AVPR1B	-0,71781979	3,02E-18
216694_at		-0,71784382	9,31E-17
207097_s_at	SLC17A2	-0,71824669	1,15E-25
217588_at	CATSPER2 /// CATSPER2P1	-0,71844669	2,72E-17
202849_x_at	GRK6	-0,71845701	1,36E-20
220747_at	HSPC072	-0,7184698	2,88E-20
207770_x_at	CSH2	-0,71858643	9,23E-22
202922_at	GCLC	-0,71930923	1,17E-06
216589_at		-0,71948757	2,07E-15
202468_s_at	CTNNAL1	-0,7195093	2,32E-05
203793_x_at	PCGF2	-0,71966268	8,21E-20
218847_at	IGF2BP2	-0,71966941	1,09E-05
210634_at	KLHL20	-0,71980962	4,62E-22
206044_s_at	BRAF	-0,71993831	1,19E-19
201187_s_at	ITPR3	-0,72015012	2,26E-20
208470_s_at	HP /// HPR	-0,72025052	0,001328077
209972_s_at	AIMP2	-0,72037639	5,39E-19
207176_s_at	CD80	-0,72047766	1,73E-19
220244_at	LOH3CR2A	-0,72070022	2,73E-21
208487_at	LMX1B	-0,72113541	2,78E-18
207198_s_at	LIMS1	-0,72119468	1,26E-17
221250_s_at	MXD3	-0,72120563	2,24E-23
215353_at		-0,7214992	3,20E-21
209855_s_at	KLK2	-0,72153419	2,37E-21
210987_x_at	TPM1	-0,72169936	1,94E-09
216961_s_at	RPAIN	-0,72232854	6,90E-20
210504_at	KLF1	-0,72284206	0,000196399
216846_at	IGLV2-18 /// IGLV3-19 /// LOC100290481	-0,72311746	2,63E-20
215909_x_at	MINK1	-0,72315172	2,54E-15
208267_at	TRPV5	-0,72319521	5,58E-26
216968_at	MASP2	-0,72346831	2,26E-21
214126_at		-0,72403324	7,85E-15
222081_at	SIRT5	-0,72432111	1,25E-22
206152_at	AGAP2	-0,72441809	4,75E-18
208364_at	INPP4A	-0,72461435	2,09E-15
220691_at		-0,72465995	1,11E-21
201373_at	PLEC	-0,72472157	1,77E-17
215233_at	JMJD6	-0,72479652	1,38E-17
218044_x_at	PTMS	-0,72528327	1,73E-20
214146_s_at	PPBP	-0,725518	6,42E-07
207670_at	KRT85	-0,72561028	1,78E-21
214223_at		-0,72580872	2,48E-19
211793_s_at	ABI2	-0,72584016	6,99E-19
207132_x_at	PFDN5	-0,72594636	6,29E-09
216427_at		-0,72624162	1,52E-23
219432_at	EVC	-0,72631372	5,77E-22

217545_at	MYH14	-0,72632234	1,56E-22
71933_at	WNT6	-0,72634643	7,06E-18
209241_x_at	MINK1	-0,72638464	5,87E-15
201402_at	ADRBK1	-0,72657702	1,08E-20
219331_s_at	KLHDC8A	-0,72671885	3,22E-15
207019_s_at	AKAP4	-0,72677766	9,34E-23
207562_at	DGKQ	-0,7268172	1,45E-20
208102_s_at	PSD	-0,72726752	1,01E-20
207329_at	MMP8	-0,72804085	0,005545106
201694_s_at	EGR1	-0,7280539	3,89E-14
208110_x_at	MED25	-0,72851722	2,79E-15
214175_x_at	PDLIM4	-0,72914105	1,54E-21
217869_at	HSD17B12	-0,72958144	1,85E-18
202961_s_at	ATP5J2	-0,72964394	3,59E-13
211693_at	IGHA1	-0,72982086	7,77E-20
218246_at	MUL1	-0,73031812	9,71E-21
203308_x_at	HPS1	-0,73112909	1,84E-12
212383_at	ATP6V0A1	-0,73141032	4,10E-23
217312_s_at	COL7A1	-0,73141368	7,94E-23
218965_s_at	TUT1	-0,73158293	2,43E-19
208118_x_at	40354 /// LOC595101 /// LOC641298 ///	-0,73196457	6,30E-27
205009_at	TFF1	-0,73196617	8,48E-18
206146_s_at	RHAG	-0,73284778	7,85E-23
220008_at	SGK269	-0,73325727	7,25E-20
216741_at		-0,73360748	1,72E-24
215276_at	WFDC8	-0,73422358	9,99E-24
218332_at	BEX1	-0,73423617	7,41E-18
206116_s_at	TPM1	-0,73431126	6,76E-09
220134_x_at	FAM176B	-0,73433704	3,10E-19
210072_at	CCL19	-0,73437825	1,73E-21
207882_at		-0,73509119	4,22E-22
203056_s_at	PRDM2	-0,73551377	7,10E-19
215461_at	ZNRF4	-0,7358054	2,69E-19
208302_at	HMHB1	-0,73607339	3,52E-23
207604_s_at	SLC4A7	-0,73607445	6,85E-14
215668_s_at	PLXNB1	-0,7363535	1,70E-18
213595_s_at	CDC42BPA	-0,73677619	1,55E-23
206929_s_at	NFIC	-0,73687254	8,96E-21
200038_s_at	RPL17	-0,73738676	5,32E-08
214421_x_at	CYP2C9	-0,73784574	2,15E-21
216789_at		-0,73792844	6,23E-19
201912_s_at	GSPT1	-0,73793761	2,83E-06
219749_at	SH2D4A	-0,73812487	2,21E-22
202409_at	IGF2 /// INS-IGF2	-0,73893946	1,65E-11
213345_at	NFATC4	-0,73928749	2,22E-19
217029_at		-0,73962611	7,14E-18
201909_at	RPS4Y1	-0,74014932	0,049975666
213722_at	SOX2	-0,74049644	5,31E-21
203692_s_at	E2F3	-0,7412334	6,01E-17
208196_x_at	NFATC1	-0,74147461	1,68E-21

1598_g_at	GAS6	-0,74167696	5,58E-18
216381_x_at	AKR7A3	-0,74234959	5,41E-20
206498_at	OCA2	-0,74237565	4,25E-20
211735_x_at	SFTPC	-0,74289044	5,59E-20
216993_s_at	COL11A2	-0,74305742	4,66E-18
206737_at	WNT11	-0,74335903	1,11E-23
220671_at	CCRN4L	-0,74348633	8,75E-22
202252_at	RAB13	-0,74434235	1,29E-14
215871_at		-0,74477671	4,64E-18
215381_at	MTOR	-0,74487249	5,35E-24
219753_at	STAG3	-0,74506443	4,00E-19
210294_at	TAPBP	-0,74520577	9,24E-14
208916_at	SLC1A5	-0,74580796	1,36E-09
203915_at	CXCL9	-0,74602086	1,45E-20
36829_at	PER1	-0,74648447	7,73E-16
208058_s_at	MGAT3	-0,74655864	1,56E-20
216643_at		-0,7466624	1,96E-24
205377_s_at	ACHE	-0,74751378	5,28E-19
221655_x_at	EPS8L1	-0,74845532	2,31E-17
213692_s_at	VDR	-0,74878317	1,39E-20
216246_at		-0,74894611	1,77E-20
208943_s_at	SEC62	-0,74907536	2,96E-07
215496_at	SAMD4A	-0,74994973	4,05E-19
200688_at	SF3B3	-0,75017052	1,20E-20
203687_at	CX3CL1	-0,75046036	2,56E-21
211448_s_at	RGS6	-0,7507283	2,86E-22
221013_s_at	APOL2	-0,75087223	4,64E-14
217373_x_at	MDM2	-0,75129819	9,34E-20
219721_at		-0,75149495	2,76E-23
218509_at	LPPR2	-0,75225379	1,12E-20
215701_at		-0,75277408	1,17E-19
207680_x_at	PAX3	-0,75325558	2,70E-21
207027_at	HGFAC	-0,75332351	1,32E-20
218266_s_at	NCS1	-0,75344949	2,84E-21
218821_at	NPEPL1	-0,75354965	1,91E-18
221393_at	TAAR3	-0,75430507	3,28E-25
214625_s_at	MINK1	-0,75440527	8,90E-17
208209_s_at	C4BPB	-0,75462282	5,93E-23
206327_s_at	CDH15	-0,75485201	3,99E-20
206342_x_at	IDS	-0,75546928	2,50E-22
206014_at	ACTL6B	-0,75552525	1,52E-21
222033_s_at	FLT1	-0,75624857	2,27E-23
213273_at	ODZ4	-0,75645162	1,12E-23
221113_s_at	WNT16	-0,75664134	2,65E-20
222280_at	LOC100506469	-0,75688079	3,31E-15
212753_at	PCGF3	-0,7570091	1,42E-13
206694_at	PNLIPRP1	-0,75755468	2,22E-20
206763_at	FKBP6	-0,75829667	9,47E-22
220442_at	GALNT4 /// POC1B	-0,75850035	1,07E-17
210499_s_at	PQBP1	-0,75880931	4,95E-23

216980_s_at	SPN	-0,75968518	4,88E-22
210684_s_at	DLG4	-0,75972539	4,76E-21
220028_at	ACVR2B	-0,75973046	2,19E-18
219975_x_at	OLAH	-0,76026627	1,30E-06
203928_x_at	MAPT	-0,76041539	3,89E-22
217611_at	ERICH1	-0,76054806	2,11E-16
211172_x_at	AKAP7	-0,76095426	6,10E-24
215027_at	RAPGEF3	-0,76124164	8,26E-22
205331_s_at	REEP2	-0,76134585	3,35E-20
221344_at	OR12D2	-0,76146361	4,09E-24
211277_x_at	APP	-0,76199887	1,58E-21
210623_at	UBXN1	-0,76201018	1,34E-20
204541_at	SEC14L2	-0,76253777	4,59E-22
220457_at	SAMD4B	-0,76283344	2,04E-21
210642_at	CCIN	-0,76308647	3,94E-24
200717_x_at	RPL7	-0,7631195	1,43E-08
203040_s_at	HMBS	-0,7632392	8,61E-09
208232_x_at		-0,76384479	5,12E-22
213494_s_at	YY1	-0,76384836	3,08E-23
217514_at		-0,7639639	5,16E-23
203655_at	XRCC1	-0,76423366	2,22E-18
220034_at	IRAK3	-0,76436994	1,24E-08
206493_at	ITGA2B	-0,76443361	2,61E-08
221162_at	HHLA1	-0,7644874	6,13E-20
211062_s_at	CPZ /// GPR78	-0,76520219	8,51E-25
221854_at	PKP1	-0,76539354	6,39E-22
206242_at	TM4SF5	-0,76570249	2,61E-22
205717_x_at	)HGA7 /// PCDHGA8 /// PCDHGA9 /// PCI	-0,76587453	9,69E-18
221960_s_at	RAB2A	-0,76649968	1,85E-15
217040_x_at	SOX15	-0,76669439	1,86E-12
AFFX-M27830_3_at		-0,76675238	7,53E-19
204334_at	KLF7	-0,76705224	2,08E-22
216925_s_at	TAL1	-0,76727969	6,18E-07
206964_at	NAT8B	-0,76753666	4,15E-17
201996_s_at	SPEN	-0,76759942	7,83E-17
214676_x_at	MUC3A	-0,7676881	1,82E-20
213441_x_at	SPDEF	-0,76820534	1,16E-20
217444_at		-0,76837837	3,47E-22
220423_at	PLA2G2D	-0,76883271	7,82E-20
220702_at		-0,76883871	1,45E-12
221232_s_at	ANKRD2	-0,76897153	9,80E-20
219775_s_at	CPLX3 /// LMAN1L	-0,76903649	8,76E-25
208104_s_at	TSC22D4	-0,76974478	4,25E-20
222319_at		-0,76975942	8,23E-22
222333_at	ALS2CL	-0,76981412	1,59E-21
215438_x_at	GSPT1	-0,76989105	8,13E-08
202859_x_at	IL8	-0,76999242	9,55E-10
202339_at	SYMPK	-0,7701376	1,61E-16
208735_s_at	CTDSP2	-0,77074023	1,33E-18
220676_at	ADAMTS8	-0,77079906	7,41E-26

208234_x_at	FGFR2	-0,7708792	2,14E-20
37796_at	LRCH4 /// SAP25	-0,77120616	5,56E-22
205384_at	FXYD1	-0,77162216	9,05E-21
209261_s_at	NR2F6	-0,77168545	2,38E-24
212086_x_at	LMNA	-0,77201533	1,23E-17
210701_at	CFDP1	-0,77251998	5,28E-09
218825_at	EGFL7	-0,77277785	3,59E-20
217206_at		-0,77286273	1,40E-20
207413_s_at	SCN5A	-0,77296031	2,69E-18
218030_at	GIT1	-0,77299932	3,46E-18
204921_at	GAS8	-0,77322452	2,63E-18
210880_s_at	EFS	-0,77325699	5,82E-23
204732_s_at	TRIM23	-0,77333294	2,08E-11
218834_s_at	TMEM132A	-0,77349977	4,24E-23
202082_s_at	SEC14L1	-0,77470375	2,82E-18
208488_s_at	CR1	-0,7753247	4,54E-09
216138_at		-0,77575551	3,69E-19
220877_at		-0,77597051	4,74E-23
214428_x_at	C4A /// C4B /// LOC100509001	-0,7760998	2,40E-06
214955_at	TMPRSS6	-0,77628395	1,18E-20
206185_at	CRYBB1	-0,77628889	4,10E-22
207914_x_at	EVX1	-0,77636502	1,39E-19
206971_at	GPR161	-0,77699277	1,09E-24
216063_at	HBBP1	-0,77729801	8,34E-15
211433_x_at	KIAA1539	-0,77757707	8,37E-19
220989_s_at	AMN	-0,77762016	6,10E-21
204943_at	ADAM12	-0,77769908	2,36E-17
202734_at	TRIP10	-0,77778144	1,06E-20
219281_at	MSRA	-0,77781946	1,81E-16
200070_at	C2orf24	-0,7790711	3,90E-14
210601_at	CDH6	-0,77924387	3,23E-23
210990_s_at	LAMA4	-0,77962152	1,69E-19
219982_s_at	SERF1A /// SERF1B	-0,77973404	2,30E-14
203342_at	TIMM17B	-0,78006747	1,45E-20
204952_at	LYPD3	-0,78016354	3,28E-20
220404_at	GPR97	-0,78029818	8,10E-10
201285_at	MKRN1	-0,78031451	8,89E-13
221835_at	DTX3	-0,78046082	5,06E-20
207742_s_at	NR6A1	-0,78072993	1,31E-26
204713_s_at	F5	-0,78085513	1,79E-09
209768_s_at	GP1BB /// SEPT5	-0,78118864	4,47E-22
220337_at	NGB	-0,78119142	1,16E-21
203878_s_at	MMP11	-0,78122522	1,08E-22
208105_at	GIPR	-0,78149556	2,67E-24
216918_s_at	DST	-0,78181531	1,48E-23
208770_s_at	EIF4EBP2	-0,78206952	1,33E-23
207004_at	BCL2	-0,78210066	4,18E-22
222004_s_at	DOCK6	-0,78235554	7,51E-20
202238_s_at	NNMT	-0,78250162	6,26E-22
212772_s_at	ABCA2	-0,78282106	1,52E-19

221128_at	ADAM19	-0,78299023	6,74E-19
207408_at	SLC22A14	-0,78325277	5,00E-20
203608_at	ALDH5A1	-0,7834765	2,94E-06
201026_at	EIF5B	-0,78348871	6,50E-14
219458_s_at	NSUN3	-0,78363431	3,81E-09
217202_s_at	GLUL	-0,78378412	7,58E-13
215434_x_at	LOC100288142 /// NBPF1 /// NBPF10	-0,7838095	3,52E-13
210776_x_at	TCF3	-0,7839941	1,31E-14
208227_x_at	ADAM22	-0,78458882	2,10E-23
219536_s_at	ZFP64	-0,78476419	6,16E-23
204696_s_at	CDC25A	-0,78477781	1,43E-22
33814_at	PAK4	-0,78478042	3,60E-19
220149_at	C2orf54	-0,78486957	7,18E-21
206725_x_at	BMP1	-0,78496959	2,58E-19
210244_at	CAMP	-0,78509553	2,34E-07
222197_s_at		-0,78524723	5,14E-24
207685_at	CRYBB3	-0,78533887	3,01E-22
206790_s_at	NDUFB1	-0,78553603	2,09E-12
201335_s_at	ARHGEF12	-0,78589885	9,85E-12
204141_at	TUBB2A	-0,78619235	0,014402778
208494_at	SLC6A7	-0,78625044	4,22E-23
213553_x_at	APOC1	-0,78662004	1,11E-19
218934_s_at	HSPB7	-0,78674241	2,72E-22
209398_at	HIST1H1C	-0,78687397	1,34E-09
31861_at	IGHMBP2	-0,78735081	1,54E-19
208100_x_at	SEMA6C	-0,78748496	2,53E-22
205262_at	KCNH2	-0,78782364	1,39E-23
201942_s_at	CPD	-0,78848102	2,53E-08
38447_at	ADRBK1	-0,78870886	2,37E-18
203973_s_at	CEBPD	-0,78879688	1,56E-17
205743_at	STAC	-0,78954023	1,88E-24
208721_s_at	ANAPC5	-0,78960696	8,68E-20
34846_at	CAMK2B	-0,79001965	8,34E-21
216137_s_at	MAPK8IP3	-0,79019717	1,63E-20
216956_s_at	ITGA2B	-0,79020764	1,08E-08
216611_s_at	SLC6A2	-0,79070771	6,65E-20
201039_s_at	RAD23A	-0,79138211	9,49E-11
207032_s_at	CRISP1	-0,79159593	9,61E-24
207658_s_at	FOXG1	-0,79219144	2,53E-23
216397_s_at	BOP1	-0,79271038	3,67E-20
211377_x_at	MYCN	-0,79328512	3,26E-24
207072_at	IL18RAP	-0,79346358	8,79E-07
218544_s_at	RCL1	-0,79374633	1,66E-21
220628_s_at	SDK2	-0,79434711	4,35E-23
217338_at	KRT19P2	-0,79438237	2,35E-21
217066_s_at	DMPK	-0,79481825	1,37E-21
214465_at	ORM1 /// ORM2	-0,79501646	7,23E-18
210411_s_at	GRIN2B	-0,79566128	6,46E-18
207057_at	SLC16A7	-0,79568978	8,66E-12
202861_at	PER1	-0,79598539	7,54E-12

207802_at	CRISP3	-0,7963982	0,000147737
202364_at	MXI1	-0,79651319	5,06E-10
216485_s_at	TPSAB1	-0,79651512	7,30E-21
220152_at	C10orf95	-0,79750813	6,92E-23
214267_s_at	CADM4	-0,79788746	1,58E-25
205081_at	CRIP1	-0,79813724	8,34E-08
204582_s_at	KLK3	-0,79832669	2,43E-24
217153_at	ARHGAP1	-0,79904889	6,08E-24
211413_s_at	PADI4	-0,79918033	3,03E-08
216747_at	APBB2	-0,79918914	5,33E-25
208003_s_at	NFAT5	-0,79919252	1,24E-13
213198_at	ACVR1B	-0,79940941	4,40E-17
216715_at		-0,79973913	3,70E-21
204901_at	BTRC	-0,80030526	1,71E-18
208327_at	CYP2A13	-0,8006789	1,99E-18
207388_s_at	PTGES	-0,8010712	3,12E-21
217006_x_at	FASN	-0,80128139	7,01E-20
209193_at	PIM1	-0,80131501	2,29E-15
222059_at	ZNF335	-0,80144567	1,05E-24
206454_s_at	RHO	-0,80159432	2,10E-21
205454_at	HPCA	-0,80164564	1,01E-20
200019_s_at	FAU	-0,8019379	5,77E-19
219613_s_at	SIRT6	-0,80206789	3,63E-22
220226_at	TRPM8	-0,80212218	2,84E-25
202729_s_at	LTBP1	-0,80216296	6,92E-21
203636_at	MID1	-0,8024814	4,60E-22
209697_at		-0,80317047	2,14E-23
215413_at	EXOC7	-0,80344429	4,30E-21
217264_s_at	SCNN1A	-0,80381649	1,99E-19
217509_x_at	GRIK5	-0,803832	2,23E-19
200796_s_at	MCL1	-0,80397841	1,12E-07
222334_at	LOC100505650	-0,80434827	1,22E-20
221068_at	KANK2	-0,80458894	3,54E-22
204774_at	EVI2A	-0,8054184	0,000288957
211993_at	WNK1	-0,80556052	9,45E-13
221830_at	RAP2A	-0,80563302	2,17E-19
206463_s_at	DHRS2	-0,80570095	8,94E-21
216490_x_at		-0,80574036	1,26E-17
206054_at	KNG1	-0,80581174	6,19E-25
217291_at	CEACAM5	-0,80616198	2,76E-24
217517_x_at	SRPK2	-0,80624649	2,15E-24
210925_at	CIITA	-0,80738125	5,70E-21
214604_at	HOXD11	-0,80812614	3,73E-24
204693_at	CDC42EP1	-0,80814826	4,37E-18
221176_x_at		-0,80846381	4,63E-22
205567_at	CHST1	-0,80903548	2,50E-24
211691_x_at		-0,80914544	9,37E-22
201551_s_at	LAMP1	-0,80925992	8,69E-09
222367_at	WHAMM /// WHAMML1 /// WHAMML2	-0,80972657	1,17E-21
206264_at	GPLD1	-0,81027243	1,33E-23

216446_at		-0,81040274	1,20E-18
206625_at	PRPH2	-0,81104278	3,34E-23
205258_at	INHBB	-0,81119241	7,14E-18
217174_s_at	APC2	-0,81249256	1,14E-21
214135_at	CLDN18	-0,81256525	1,66E-22
211590_x_at	TBXA2R	-0,8134778	5,90E-20
211792_s_at	CDKN2C	-0,81362048	2,71E-21
210783_x_at	CLEC11A	-0,81399932	5,78E-24
209035_at	MDK	-0,8143957	2,85E-15
200917_s_at	SRPR	-0,81447941	1,59E-17
222007_s_at	FKBP8	-0,81453757	7,75E-24
202500_at	DNAJB2	-0,81542217	9,26E-12
202917_s_at	S100A8	-0,81563151	2,72E-11
220705_s_at	ADAMTS7	-0,81678499	2,45E-24
221791_s_at	CCDC72	-0,81788772	1,67E-16
210774_s_at	NCOA4	-0,81789648	4,93E-17
220524_at	EPB41L4B	-0,81800088	7,84E-25
221715_at		-0,81867037	9,47E-23
219075_at	YIPF2	-0,81896226	9,99E-19
220395_at	DNAJA4	-0,81984377	9,70E-20
210810_s_at	SLC6A5	-0,81990765	1,61E-22
205582_s_at	GGT5	-0,820021	8,09E-21
217389_s_at	ATF5	-0,82019573	9,79E-23
206080_at	PLCH2	-0,82044226	9,68E-23
219320_at	MYO19	-0,82044506	1,64E-21
220573_at	KLK14	-0,82068776	8,92E-21
210086_at	HR	-0,82070526	3,88E-20
220988_s_at	C1QTNF3	-0,82108688	1,76E-22
44702_at	SYDE1	-0,82115051	4,12E-20
218644_at	PLEK2	-0,82137933	1,25E-07
207789_s_at	DPP6	-0,82148499	2,34E-24
216940_x_at	YBX1 /// YBX1P2	-0,82164928	1,96E-24
204288_s_at	SORBS2	-0,82168444	6,10E-22
204778_x_at	HOXB7	-0,82228636	8,45E-22
220971_at	IL25	-0,82231957	2,32E-22
201665_x_at	RPS17	-0,82232098	9,24E-09
216995_x_at	MKRN2	-0,82240606	1,82E-20
214536_at	SLURP1	-0,82241665	2,97E-22
203542_s_at	KLF9	-0,82249098	4,15E-13
217430_x_at	COL1A1	-0,82307316	2,14E-18
217622_at		-0,82392456	4,16E-23
211705_s_at	SORBS1	-0,82400973	3,39E-22
209812_x_at	CASP2	-0,82421903	1,82E-22
202002_at	ACAA2	-0,82436371	2,31E-23
212558_at	SPRY1	-0,82450504	9,08E-22
214840_at	TOM1L2	-0,82474488	5,56E-24
216759_at	HRASLS2	-0,82541155	1,84E-25
211677_x_at	CADM3	-0,8254714	2,84E-19
221906_at	TXNRD3	-0,82551498	8,62E-21
218624_s_at	MGC2752	-0,82587664	1,08E-22

210869_s_at	MCAM	-0,82589665	3,03E-20
206753_at	RDH16	-0,82602063	6,43E-20
203691_at	PI3	-0,82679254	0,000403858
209802_at	PHLDA2	-0,82682682	4,85E-21
215620_at	RREB1	-0,82751689	1,55E-17
220308_at	CCDC19	-0,82802875	3,12E-20
215045_at	CELF3	-0,82824032	1,18E-20
206517_at	CDH16	-0,82892757	1,26E-21
218302_at	PSENEN	-0,82905945	1,92E-15
211096_at	PBX2	-0,82922255	7,58E-23
203478_at	NDUFC1	-0,8293257	5,53E-14
214819_at	IQSEC2	-0,82961643	3,51E-21
205774_at	F12	-0,83006154	1,93E-23
217135_x_at		-0,83053844	3,98E-19
221707_s_at	VPS53	-0,83065945	3,08E-17
222022_at	DTX3	-0,83080082	9,78E-20
201868_s_at	TBL1X	-0,83151662	3,38E-13
205537_s_at	VAV2	-0,83152767	5,41E-23
202752_x_at	SLC7A8	-0,8324093	1,08E-17
219039_at	SEMA4C	-0,83257285	2,80E-17
206531_at	DPF1	-0,83276695	7,99E-22
206741_at	C3orf32	-0,83300497	8,02E-20
218157_x_at	CDC42SE1	-0,83301095	9,90E-22
208565_at	MC5R	-0,83348832	5,44E-21
214893_x_at	HCN2	-0,83405367	2,35E-24
216419_at	CROCC	-0,83410989	3,28E-21
200831_s_at	SCD	-0,83421593	1,14E-19
217462_at	C11orf9	-0,83437426	1,53E-21
221061_at	PKD2L1	-0,8352051	7,25E-20
216361_s_at	MYST3	-0,83653281	1,47E-17
217464_at		-0,83667532	6,02E-20
216707_at		-0,83692744	7,58E-19
211992_at	WNK1	-0,83733378	1,69E-14
214125_s_at	NENF	-0,83744679	1,83E-19
221309_at	RBM17	-0,83772253	1,35E-20
206331_at	CALCRL	-0,83843749	3,26E-23
217773_s_at	NDUFA4	-0,83883998	3,20E-07
205236_x_at	SOD3	-0,83887484	5,87E-20
220510_at	RHBG	-0,83893319	8,75E-19
210056_at	RND1	-0,83934976	1,06E-22
206217_at	EDA	-0,83945711	1,17E-23
208924_at	RNF11	-0,83957357	2,18E-05
210689_at	CLDN14	-0,83981268	8,01E-25
219630_at	PDZK1IP1	-0,84005154	2,91E-06
210727_at	CALCA	-0,84035112	3,11E-22
213147_at	HOXA10	-0,84098481	4,34E-22
211820_x_at	GYPA	-0,8410648	6,76E-08
212498_at		-0,84212527	8,22E-14
211589_at	PML	-0,84214813	4,41E-22
205276_s_at	GTPBP1	-0,84221006	1,75E-20

221419_s_at		-0,84235875	1,39E-16
203945_at	ARG2	-0,84293452	3,90E-20
204947_at	E2F1	-0,84317151	2,95E-21
212713_at	MFAP4	-0,84369581	2,11E-23
220130_x_at	LTB4R2	-0,84393794	2,00E-20
204870_s_at	PCSK2	-0,84420835	2,27E-26
210136_at	MBP	-0,84423689	1,99E-15
210908_s_at	PFDN5	-0,844733	3,27E-11
213777_s_at		-0,84532258	1,49E-21
220635_at	PSORS1C2	-0,8456264	2,96E-23
221967_at	NXPH4	-0,84574839	2,53E-23
212968_at	RFNG	-0,84594828	5,87E-21
201122_x_at	EIF5A	-0,84598482	1,38E-12
209477_at	EMD	-0,8462841	2,14E-16
212763_at	CAMSAP1L1	-0,84659683	2,75E-23
222099_s_at	LSM14A	-0,84665593	2,27E-26
212219_at	PSME4	-0,84684983	1,68E-11
203149_at	PVRL2	-0,84692616	5,02E-15
203899_s_at	CRCP	-0,84745308	2,67E-19
201042_at	TGM2	-0,84804658	4,38E-10
216641_s_at	LAD1	-0,8481514	1,23E-22
216481_at	GRIP2	-0,8482255	3,09E-21
215078_at	SOD2	-0,84897207	4,53E-11
214619_at	CRHR1	-0,84922001	1,97E-24
220203_at	BMP8A	-0,8492237	2,25E-22
216630_at		-0,84924692	1,16E-24
206371_at	FOLR3	-0,8497773	7,71E-05
218843_at	FNDC4	-0,85009127	1,95E-18
216680_s_at	EPHB4	-0,85094052	1,00E-22
220694_at	ASAP1-IT	-0,85124271	1,25E-12
216887_s_at	LDB3	-0,85135499	1,75E-22
215037_s_at	BCL2L1	-0,85179643	1,91E-05
205856_at	SLC14A1	-0,85180246	5,04E-06
201684_s_at	TOX4	-0,85189851	4,76E-16
212151_at	PBX1	-0,85224513	7,99E-10
220379_at	FSCN3	-0,85268443	5,85E-23
217447_at	MAG	-0,85320637	1,77E-23
217998_at	PHLDA1	-0,85334392	3,07E-21
202130_at	RIOK3	-0,85373758	1,67E-08
211487_x_at	LOC100505503 /// RPS17	-0,85426772	2,39E-09
214463_x_at	HIST1H4J	-0,8543488	7,10E-13
215489_x_at	HOMER3	-0,85469484	5,39E-16
219241_x_at	SSH3	-0,8548177	1,88E-22
208710_s_at	AP3D1	-0,85582065	1,54E-20
216215_s_at	RBFOX2	-0,85582868	5,29E-19
203725_at	GADD45A	-0,85588646	5,08E-08
204551_s_at	AHSG	-0,85601469	4,96E-23
206647_at	HBZ	-0,85636889	0,0001839
219943_s_at	USP46	-0,85647638	3,37E-23
216473_x_at	/// DUX4L3 /// DUX4L4 /// DUX4L5 /// DU	-0,8564946	1,41E-22

216317_x_at	RHCE	-0,85668168	4,07E-13
209225_x_at	TNPO1	-0,85721022	4,42E-21
219365_s_at	CAMKV	-0,85737037	4,11E-21
212578_x_at	LOC100505503 /// RPS17	-0,85756945	1,06E-09
210907_s_at	PDCD10	-0,85773356	8,46E-07
215819_s_at	RHCE /// RHD	-0,8586059	4,34E-08
211260_at	BMP7	-0,86017305	2,01E-21
222002_at	C7orf26	-0,86020146	3,03E-22
221023_s_at	KCNH6	-0,86045431	6,17E-25
31637_s_at	NR1D1 /// THRA	-0,86104668	1,31E-17
218507_at	C7orf68	-0,86123666	2,98E-20
210030_at		-0,86133778	5,10E-23
221792_at	RAB6B	-0,86137193	8,79E-22
213551_x_at	PCGF2	-0,86293918	4,13E-20
218136_s_at	SLC25A37	-0,86342915	8,87E-16
206213_at	WNT10B	-0,86353545	5,37E-20
222297_x_at		-0,86383706	3,78E-15
220626_at	SERPINA10	-0,86526907	5,75E-25
210180_s_at	TRA2B	-0,86578125	1,18E-14
211410_x_at	KIR2DL5A	-0,86609337	3,01E-14
40850_at	FKBP8	-0,8661009	2,49E-10
205845_at	CACNA1H	-0,86723448	1,17E-22
204302_s_at	KIAA0427	-0,86777051	4,02E-22
207861_at	CCL22	-0,86787448	1,15E-25
221237_s_at	OSBP2	-0,86787515	1,65E-09
213288_at	MBOAT2	-0,86797076	1,24E-20
205001_s_at	DDX3Y	-0,86916229	2,00E-09
210746_s_at	EPB42	-0,8698824	6,88E-06
213033_s_at	NFIB	-0,87059597	5,69E-22
206050_s_at	RNH1	-0,87078494	1,03E-12
211053_at	KCNG1	-0,87107691	6,42E-22
61874_at	C9orf7	-0,8711937	1,46E-21
217018_at		-0,8715513	6,88E-23
209244_s_at	KIF1C	-0,87163413	9,74E-20
205332_at	RCE1	-0,87204654	2,25E-21
205721_at	GFRA2	-0,87235691	1,76E-23
203757_s_at	CEACAM6	-0,87242441	1,25E-06
207634_at	PDCD1	-0,87243564	1,35E-22
201736_s_at	MARCH6	-0,87255376	1,20E-23
216911_s_at	HIC2	-0,87266073	4,41E-22
213344_s_at	H2AFX	-0,87321736	1,61E-21
219373_at	DPM3	-0,87348207	9,13E-16
217011_at	GBX1	-0,87356124	3,57E-22
215083_at		-0,87435637	2,55E-12
222109_at	GNL3L	-0,87473064	2,15E-23
214957_at	ACTL8	-0,87522233	2,06E-22
215649_s_at	MVK	-0,87551904	3,26E-23
219225_at	PGBD5	-0,8773527	5,71E-22
201262_s_at	BGN	-0,87830943	1,25E-25
220514_at	LOC100508936	-0,87844258	8,98E-21

206986_at	FGF18	-0,8791261	1,76E-23
215181_at	CDH22	-0,87930308	2,51E-24
217055_x_at		-0,8794906	9,71E-21
219070_s_at	MOSPD3	-0,87972853	3,69E-24
217658_at	THAP3	-0,88044756	4,96E-21
65517_at	AP1M2	-0,88118542	1,20E-16
206161_s_at	SYT5	-0,88176246	1,70E-23
220662_s_at	HEYL	-0,882396	3,27E-22
208111_at	AVPR2	-0,88271393	4,30E-19
221824_s_at	MARCH8	-0,88300064	1,10E-10
214630_at	CYP11B2	-0,88404226	5,97E-20
215372_x_at		-0,88411572	2,25E-22
205879_x_at	RET	-0,88433702	3,83E-21
203247_s_at	ZNF24	-0,88478885	3,24E-19
207532_at	CRYGD	-0,8850708	1,50E-24
221227_x_at	COQ3	-0,88516863	1,00E-20
207367_at	ATP12A	-0,88518957	4,30E-22
204147_s_at	TFDP1	-0,88528682	3,79E-14
212444_at		-0,88533877	6,77E-23
210794_s_at	MEG3	-0,8855891	1,01E-22
203662_s_at	TMOD1	-0,88612588	4,94E-07
221163_s_at	MLXIPL	-0,88616652	9,75E-20
204574_s_at	MMP19	-0,88664405	2,36E-21
208257_x_at	PSG1	-0,8867816	8,05E-24
216888_at	LDB3	-0,88847987	4,77E-23
221063_x_at	RNF123	-0,88896888	1,34E-11
217198_x_at	GHD /// IGHG1 /// LOC100126583 /// LOC	-0,88911065	5,29E-19
207087_x_at	ANK1	-0,88931743	4,41E-10
208544_at	ADRA2B	-0,88969577	3,47E-21
200085_s_at	TCEB2	-0,88978292	3,21E-16
45297_at	EHD2	-0,89067632	7,55E-23
201635_s_at	FXR1	-0,89084299	1,21E-20
215999_at	CDRT1	-0,89095676	1,97E-22
212570_at	ENDOD1	-0,89146067	1,85E-14
215232_at	ARHGAP44	-0,8924519	5,78E-23
219847_at	HDAC11	-0,89262242	3,74E-23
221956_at	LRCH4	-0,8927671	2,20E-19
200896_x_at	HDGF	-0,893666	1,20E-18
38487_at	STAB1	-0,89401236	9,07E-14
206814_at	NGF	-0,89410114	4,28E-24
220968_s_at	TSPAN9	-0,89440308	2,42E-21
205852_at	CDK5R2	-0,89455148	5,43E-21
214294_at	KIAA0485	-0,89460757	5,82E-23
213866_at	SAMD14	-0,89510904	2,88E-21
206399_x_at	CACNA1A	-0,89515756	1,77E-21
208781_x_at	SNX3	-0,89586054	3,49E-19
211049_at	TLX2	-0,8958866	1,86E-23
207586_at	SHH	-0,89612051	1,57E-23
211008_s_at	UBE2I	-0,89620706	1,31E-21
220096_at		-0,89725774	1,04E-22

202125_s_at	TRAK2	-0,89785978	6,80E-08
216318_at	IGHA1	-0,89789782	1,97E-23
219298_at	ECHDC3	-0,89827181	1,52E-10
219579_at	RAB3IL1	-0,89873902	4,75E-19
208942_s_at	SEC62	-0,89910578	1,00E-11
221142_s_at	PECR	-0,89966035	2,55E-22
214993_at	ASPHD1	-0,89983294	1,38E-23
AFFX-HUMRGE/M10098_5_at		-0,90034617	5,46E-17
219921_s_at	DOCK5	-0,90145752	1,50E-17
209490_s_at	EGFL8 /// PPT2	-0,90293444	3,80E-20
207554_x_at	TBXA2R	-0,90309806	5,53E-19
214187_x_at	CTDSPL	-0,90482804	2,49E-23
213809_x_at	TCF3	-0,90494847	7,07E-25
217354_s_at	HPS1	-0,90538331	3,85E-11
221560_at	MARK4	-0,90570306	2,54E-22
205915_x_at	GRIN1	-0,9058289	6,10E-25
213611_at	AQP5	-0,90638176	1,40E-21
219707_at	CPNE7	-0,90642868	3,91E-22
35201_at	HNRNPL	-0,9064352	1,70E-17
209979_at	ADARB1	-0,90771172	3,52E-23
206913_at	BAAT	-0,90788099	1,87E-23
632_at	GSK3A	-0,90811193	6,68E-21
220778_x_at	SEMA6B	-0,90832988	1,37E-21
201409_s_at	PPP1CB	-0,90845547	4,53E-08
211287_x_at	CSF2RA	-0,90870594	1,60E-23
210254_at	MS4A3	-0,90915038	2,51E-06
215328_at	EFR3B	-0,90928581	1,51E-20
202199_s_at	SRPK1	-0,91056128	8,70E-18
206469_x_at	AKR7A3	-0,91093701	8,96E-21
215049_x_at	CD163	-0,91102385	7,64E-11
201416_at	SOX4	-0,91173987	2,35E-15
211816_x_at	FCAR	-0,91204953	1,37E-11
210480_s_at	MYO6	-0,91211456	1,23E-23
218819_at	INTS6	-0,91233407	1,36E-16
215205_x_at	NCOR2	-0,91362903	6,42E-24
210412_at	GRIN2B	-0,91429292	1,19E-21
209191_at	TUBB6	-0,9146575	1,39E-17
217672_x_at	EIF1	-0,91481955	2,32E-20
222107_x_at	LZTS1	-0,91541733	6,59E-22
211253_x_at	PYY	-0,9157548	4,13E-20
214072_x_at	NENF	-0,91606253	3,51E-19
207154_at	DIO3	-0,91614532	3,91E-23
211198_s_at	ICOSLG	-0,91628118	4,87E-24
213753_x_at	EIF5A	-0,91655203	1,09E-15
213108_at	CAMK2A	-0,91660844	1,05E-24
221034_s_at	TEX13B	-0,91719947	4,71E-24
217348_x_at	ARHGEF15	-0,91723859	4,10E-23
208379_x_at	NPY2R	-0,91808262	1,41E-21
204648_at	NPR1	-0,91844175	4,79E-24
220246_at	CAMK1D	-0,91894551	8,50E-16

216881_x_at	PRB4	-0,91961122	3,75E-20
220849_at	LOC79999	-0,91998719	1,17E-20
202844_s_at	RALBP1	-0,92069552	2,31E-23
208404_x_at	KCNJ5	-0,92097134	4,12E-25
219437_s_at	ANKRD11	-0,92134562	2,98E-20
216955_at	TAF1	-0,92224447	2,94E-23
200697_at	HK1	-0,92288398	7,99E-22
215323_at	LUZP2	-0,92328548	3,02E-25
220207_at	YIF1B	-0,92387566	5,04E-24
203527_s_at	APC	-0,92400405	1,73E-23
220173_at	C14orf45	-0,92419105	2,38E-06
200888_s_at	RPL23	-0,92461504	4,22E-11
210862_s_at	SARDH	-0,92487258	8,44E-24
221871_s_at	TFG	-0,92494277	1,44E-24
222271_at		-0,92495521	5,52E-23
201257_x_at	RPS3A	-0,9259373	2,06E-07
209936_at	RBM5	-0,92620611	3,88E-14
201452_at	RHEB	-0,92640656	1,40E-24
204997_at	GPD1	-0,92766178	2,32E-22
215265_at	EMX1	-0,92835023	5,74E-22
211396_at	FCGR2C	-0,92900959	1,32E-19
208670_s_at	EID1	-0,92929693	2,40E-22
200099_s_at	RPS3A	-0,92968531	2,62E-06
201482_at	QSOX1	-0,92977995	2,25E-22
202028_s_at	RPL38	-0,92995006	1,64E-18
212312_at	BCL2L1	-0,93102718	1,47E-11
201418_s_at	SOX4	-0,93237899	6,19E-22
207244_x_at	CYP2A6	-0,93251167	1,33E-23
202435_s_at	CYP1B1	-0,93292976	6,07E-09
217503_at	STK17B	-0,93341266	2,03E-10
202110_at	COX7B	-0,93362028	8,10E-09
205604_at	HOXD9	-0,93391009	1,28E-21
209811_at	CASP2	-0,93396693	3,58E-13
202045_s_at	GRLF1	-0,93404534	3,14E-25
207321_s_at	ABCB9	-0,9340947	6,80E-21
202827_s_at	MMP14	-0,93415339	2,43E-24
201431_s_at	DPYSL3	-0,93446829	8,64E-21
216779_at	CYLC1	-0,9345923	3,38E-22
214843_s_at	USP33	-0,93567225	6,76E-21
217249_x_at		-0,93592649	4,11E-16
204615_x_at	IDI1	-0,93614421	1,18E-09
210782_x_at	GRIN1	-0,93647093	7,73E-23
216484_x_at	HDGF	-0,93699199	3,37E-19
209475_at	USP15	-0,93733238	1,28E-10
204187_at	GMPR	-0,93797512	5,58E-08
209843_s_at	SOX10	-0,93939358	5,67E-22
214694_at	MPRIP	-0,93996371	4,15E-19
204466_s_at	SNCA	-0,94017748	1,56E-08
221100_at	C6orf15	-0,94065114	2,71E-21
214390_s_at	BCAT1	-0,94086803	4,19E-29

396_f_at	EPOR	-0,94236687	4,40E-17
217491_x_at	COX7C	-0,9425008	5,96E-11
220371_s_at	SLC12A9	-0,94283101	1,49E-22
215557_at		-0,94354904	4,99E-23
209913_x_at	KIAA0415	-0,94384678	4,92E-20
212973_at	RPIA	-0,94389365	1,28E-12
206747_at	GPRIN2	-0,94427249	2,55E-21
40560_at	TBX2	-0,94766446	5,70E-24
213310_at	EIF2C2	-0,94798443	8,19E-13
206916_x_at	TAT	-0,94859681	5,03E-23
210483_at	LOC254896	-0,94941519	8,02E-17
207024_at	CHRND	-0,94948016	2,25E-22
213260_at	FOXC1	-0,95038929	1,45E-22
206827_s_at	TRPV6	-0,95048839	2,13E-24
208414_s_at	HOXB3	-0,95055584	2,42E-23
211111_at	HGC6.3	-0,95117906	3,03E-24
208474_at	CLDN6	-0,95157984	1,28E-22
214883_at	THRA	-0,95214743	4,59E-24
206813_at	CTF1	-0,95545648	4,21E-23
216631_s_at		-0,95657206	5,18E-23
221362_at	HTR5A	-0,95850971	3,12E-27
203979_at	CYP27A1	-0,95935979	9,97E-21
214807_at		-0,96015672	1,46E-16
210332_at	ACHE	-0,96067325	1,14E-21
211313_s_at	BAZ1B	-0,96129997	7,91E-22
210193_at	MOBP	-0,96183173	7,21E-24
221660_at	MYL10	-0,96213381	4,92E-22
216584_at		-0,96248488	1,97E-22
215771_x_at	RET	-0,96256368	3,20E-22
200976_s_at	TAX1BP1	-0,96313894	6,17E-18
201061_s_at	STOM	-0,96322989	4,43E-12
201597_at	COX7A2	-0,96348193	3,43E-10
205967_at	HIST1H4C	-0,96390328	3,29E-12
210943_s_at	LYST	-0,96407846	3,88E-20
212391_x_at	RPS3A	-0,96445505	1,13E-07
204131_s_at	FOXO3	-0,96475754	3,65E-17
41657_at	STK11	-0,96492743	7,91E-21
203464_s_at	EPN2	-0,96502813	2,73E-24
204121_at	GADD45G	-0,96514601	6,10E-22
78383_at	LOC100129250	-0,9652503	3,29E-25
35776_at	ITSN1	-0,96565266	5,19E-21
221962_s_at	UBE2H	-0,96599001	2,42E-13
208541_x_at	TFAM	-0,9662167	2,30E-24
214634_at		-0,96738472	1,21E-22
221819_at	RAB35	-0,96808015	7,02E-18
215863_at	TFR2	-0,96879445	2,48E-25
206709_x_at	GPT	-0,96891173	9,81E-24
220790_s_at	MS4A5	-0,96912804	8,01E-25
207203_s_at	NR1I2	-0,96932964	2,92E-21
206650_at	IQCC	-0,96946102	2,24E-20

220529_at	FLJ11710	-0,96981505	5,30E-23
213171_s_at	MMP24	-0,97062964	2,57E-26
210618_at	RAP1GAP	-0,97070642	2,26E-23
207184_at	SLC6A13	-0,97080778	8,65E-22
207767_s_at	EGR4	-0,97238412	5,73E-21
207931_s_at	PFKFB2	-0,97301804	3,16E-23
204189_at	RARG	-0,97352686	1,60E-18
215875_at		-0,97461146	2,46E-24
40093_at	BCAM	-0,97489758	1,37E-22
212290_at	SLC7A1	-0,9750038	8,17E-24
202456_s_at	ZER1	-0,97517566	2,11E-13
216924_s_at	DRD2	-0,97529473	2,79E-23
206882_at	SLC1A6	-0,97532839	7,78E-23
208537_at	S1PR2	-0,97694386	8,08E-25
208344_x_at	IFNA1 /// IFNA13	-0,97741791	1,11E-23
219323_s_at	IL18BP	-0,97748079	4,86E-23
204132_s_at	FOXO3 /// FOXO3B	-0,97789456	5,47E-14
203618_at	FAIM2	-0,97835631	2,03E-23
212473_s_at	MICAL2	-0,97869231	2,24E-15
205570_at	PIP4K2A	-0,97869878	5,76E-10
206437_at	S1PR4	-0,97972001	2,78E-19
34449_at	CASP2	-0,98047009	9,70E-19
220751_s_at	C5orf4	-0,9806708	4,14E-08
213792_s_at	INSR	-0,98071211	7,91E-22
203096_s_at	RAPGEF2	-0,9807222	1,29E-11
205620_at	F10	-0,98091182	2,31E-23
202448_s_at	ZER1	-0,98111088	3,67E-17
219699_at	LGI2	-0,98179187	8,98E-23
210761_s_at	GRB7	-0,98364176	8,27E-22
214064_at	TF	-0,98443265	3,18E-25
216889_s_at	HNF4A	-0,98501079	1,61E-23
220420_at	LMAN1L	-0,98501738	4,26E-24
205900_at	KRT1	-0,98631565	5,72E-06
213978_at	MTSS1L	-0,986406	3,52E-25
220627_at	CST8	-0,98769438	1,15E-25
209718_at	NCAPH2	-0,98951453	4,52E-23
201638_s_at	CPSF1	-0,98979099	2,55E-23
209086_x_at	MCAM	-0,98989377	6,02E-23
206903_at	EXOG	-0,99048755	3,83E-23
209266_s_at	SLC39A8	-0,99102832	9,62E-25
213592_at	APLNR	-0,99125594	1,44E-23
213779_at	EMID1	-0,99152358	3,31E-21
209079_x_at	)HGA7 /// PCDHGA8 /// PCDHGA9 /// PCI	-0,99172368	1,09E-21
205388_at	TNNC2	-0,99181588	2,88E-24
215003_at	DGCR9	-0,9921496	1,23E-23
209339_at	SIAH2	-0,99279215	3,69E-11
204899_s_at	SAP30	-0,99289573	7,77E-20
204371_s_at	KHSRP	-0,99321192	8,92E-21
221192_x_at	MFSD11	-0,99366028	7,36E-22
38691_s_at	SFTPC	-0,9939421	5,70E-23

206494_s_at	ITGA2B	-0,99548748	8,01E-10
213941_x_at	RPS7	-0,99576177	1,27E-08
218707_at	ZNF444	-0,9959065	3,30E-19
214027_x_at	DES /// FAM48A	-0,99720518	4,57E-21
218418_s_at	KANK2	-0,99771649	6,23E-10
208285_at	OR7A5	-1,00155181	1,24E-21
205982_x_at	SFTPC	-1,0048427	4,23E-23
206970_at	CNTN2	-1,00643324	1,17E-21
212330_at	TFDP1	-1,00672492	1,32E-12
214368_at	RASGRP2	-1,00923442	5,38E-23
221109_at	LOC100506571	-1,00929129	1,70E-22
219500_at	CLCF1	-1,00959875	3,52E-24
213327_s_at	USP12	-1,00967565	5,24E-11
201940_at	CPD	-1,01034923	4,32E-11
211066_x_at	)HGA7 /// PCDHGA8 /// PCDHGA9 /// PCI	-1,01392675	1,98E-20
201952_at	ALCAM	-1,01496323	5,34E-14
222375_at		-1,0179164	1,34E-20
220002_at	KIF26B	-1,02025225	1,50E-24
221058_s_at	CKLF	-1,02061751	3,64E-17
205403_at	IL1R2	-1,02107248	1,14E-08
207476_at	LOC100507630	-1,02109677	4,04E-21
220017_x_at	CYP2C9	-1,02269633	6,53E-22
202187_s_at	PPP2R5A	-1,02373256	1,11E-23
215085_x_at	DLEC1	-1,02421135	4,60E-24
211439_at	SRSF7	-1,02441022	1,42E-21
203835_at	LRRC32	-1,02475249	1,91E-23
207876_s_at	FLNC	-1,02713491	4,47E-25
210695_s_at	WWOX	-1,02739253	6,58E-21
213240_s_at	KRT4	-1,02742121	1,67E-24
210367_s_at	PTGES	-1,0282225	6,78E-25
206656_s_at	C20orf3	-1,02825627	6,57E-22
218563_at	NDUFA3	-1,02831084	7,03E-13
220563_s_at	SHANK1	-1,03013919	4,02E-25
204647_at	HOMER3	-1,03051306	4,51E-19
221306_at	GPR27	-1,03112279	1,09E-23
213998_s_at	DDX17	-1,03225047	6,12E-18
206697_s_at	HP	-1,03331636	2,24E-06
201148_s_at	TIMP3	-1,03502386	4,83E-22
212468_at	SPAG9	-1,03505399	5,04E-19
205844_at	VNN1	-1,03539069	9,29E-06
201134_x_at	COX7C	-1,03592587	3,18E-14
204856_at	B3GNT3	-1,03639594	8,27E-24
206227_at	CILP	-1,03710213	9,05E-22
217889_s_at	CYBRD1	-1,03734185	6,22E-13
208057_s_at	GLI2	-1,04044175	6,12E-22
212445_s_at	NEDD4L	-1,04140029	8,81E-14
216272_x_at	SYDE1	-1,04151791	7,45E-25
209643_s_at	PLD2	-1,04338782	5,11E-25
220944_at	PGLYRP4	-1,0434406	3,51E-21
216998_s_at	ADAM5P	-1,04460822	1,30E-24

206635_at	CHRNB2	-1,04551528	1,15E-23
209184_s_at	IRS2	-1,04728278	5,15E-16
206338_at	ELAVL3	-1,04853658	6,88E-22
202881_x_at		-1,04862671	1,19E-24
220848_x_at	OBP2A	-1,04910131	5,38E-23
212831_at	MEGF9	-1,05078594	3,06E-18
202204_s_at	AMFR	-1,05122697	4,23E-22
219781_s_at	ZNF771	-1,05248201	5,38E-23
221650_s_at	MED18	-1,05445604	1,45E-21
220570_at	RETN	-1,05476781	4,64E-07
217507_at	SLC11A1	-1,05496542	1,57E-11
217256_x_at		-1,05507978	2,53E-19
221932_s_at	GLRX5	-1,0557526	9,44E-12
209877_at	SNCG	-1,05597046	1,92E-23
217683_at	HBE1	-1,05786349	3,41E-25
205575_at	C1QL1	-1,0582762	8,00E-26
215685_s_at	DLX2	-1,06100347	1,41E-22
217399_s_at	FOXO3 /// FOXO3B	-1,06143189	1,02E-21
201045_s_at	RAB6A	-1,06276267	1,18E-23
221051_s_at	ITGB1BP3	-1,06340235	3,75E-24
220323_at	CNTD2	-1,06485223	3,04E-23
203425_s_at	IGFBP5	-1,06510939	1,27E-23
216882_s_at	NEBL	-1,06635666	1,32E-25
213096_at	TMCC2	-1,06651669	2,63E-10
220554_at	SLC22A7	-1,06901198	3,35E-24
220562_at	CYP2W1	-1,06943334	6,16E-23
220798_x_at	LPPR3	-1,07219546	8,30E-25
212654_at	TPM2	-1,07369689	6,89E-21
211322_s_at	SARDH	-1,07376289	3,11E-26
200963_x_at	RPL31	-1,07399131	8,94E-10
211248_s_at	CHRD	-1,07495673	1,81E-23
208727_s_at	CDC42	-1,07526749	5,83E-11
204140_at	TPST1	-1,07547577	5,04E-14
217701_x_at		-1,07581011	1,33E-15
221009_s_at	ANGPTL4	-1,07610435	3,28E-25
204292_x_at	STK11	-1,07625061	1,42E-23
216046_at		-1,07719066	1,17E-23
215021_s_at	NRXN3	-1,07806398	2,18E-23
221425_s_at	ISCA1	-1,07913897	6,35E-09
211074_at	FOLR1	-1,07918264	9,77E-18
213783_at	MFNG	-1,08250274	7,17E-24
221717_at		-1,08283022	1,09E-20
209359_x_at	RUNX1	-1,08449441	1,61E-19
210226_at	NR4A1	-1,08770836	4,99E-22
201492_s_at	RPL41	-1,08891091	2,14E-15
218583_s_at	DCUN1D1	-1,08912409	4,57E-13
217534_at	FAM49B	-1,09269703	2,22E-19
210430_x_at	RHD	-1,09299386	1,41E-14
202191_s_at	GAS7	-1,09315267	2,17E-19
209890_at	TSPAN5	-1,09321475	1,16E-10

160020_at	MMP14	-1,09453644	1,66E-22
215026_x_at	SCNN1A	-1,09455954	3,32E-23
221446_at	ADAM30	-1,09457779	1,31E-25
215061_at	METTL10	-1,09643741	3,74E-25
221376_at	FGF17	-1,09932519	1,58E-25
210781_x_at	GRIN1	-1,10100651	6,20E-23
201882_x_at	B4GALT1	-1,10280327	7,70E-27
205314_x_at	SNTB2	-1,10529243	2,40E-27
221101_at		-1,10560321	3,99E-21
220325_at	TAF7L	-1,10702202	1,07E-23
215740_at	LOC100130741	-1,10764678	4,36E-25
205973_at	FEZ1	-1,10772719	1,81E-23
202452_at	ZER1	-1,10868448	2,10E-20
201812_s_at	C4orf46 /// TOMM7	-1,10913698	1,69E-15
205629_s_at	CRH	-1,10921734	2,68E-24
203936_s_at	MMP9	-1,11018438	1,52E-09
221108_at	C22orf43	-1,11103999	2,33E-23
217406_at		-1,11134766	6,89E-25
203502_at	BPGM	-1,11205583	3,50E-06
216385_at	LOXL3	-1,11266532	1,67E-22
213767_at	KSR1	-1,11298629	3,60E-23
214237_x_at	PAWR	-1,11319267	6,89E-25
220453_at	PQLC2	-1,11438877	6,37E-23
202482_x_at	RANBP1	-1,11749183	1,06E-24
207827_x_at	SNCA	-1,1187723	9,06E-10
206302_s_at	NUDT4 /// NUDT4P1	-1,12072711	3,75E-08
217801_at	ATP5E	-1,12386781	4,60E-14
214403_x_at	SPDEF	-1,12388931	3,63E-24
205917_at	ZNF264	-1,12391571	1,88E-17
209730_at	SEMA3F	-1,12425912	1,35E-26
217661_x_at	SIX5	-1,12619262	3,75E-25
213013_at	LOC644172 /// MAPK8IP1	-1,12701263	4,98E-19
205117_at	FGF1	-1,12893475	1,66E-23
202029_x_at	RPL38	-1,13040148	6,33E-17
208242_at	RAX	-1,13100127	8,52E-24
221379_at		-1,13162791	2,37E-23
216078_at		-1,13185234	3,28E-25
221478_at	BNIP3L	-1,13375618	2,19E-09
206368_at	CPLX2	-1,13400124	1,23E-24
216855_s_at	HNRNPU	-1,13561904	3,19E-27
206739_at	HOXC5	-1,13611374	3,59E-23
205998_x_at	CYP3A4	-1,13634149	2,68E-23
219488_at	A4GALT	-1,13757612	8,30E-27
201406_at	RPL36A	-1,13760856	4,30E-10
214349_at		-1,13891957	4,54E-24
214927_at	ITGBL1	-1,14035591	7,43E-25
206328_at	CDH15	-1,14074702	7,12E-26
220743_at		-1,1419759	3,91E-24
208134_x_at	PSG2	-1,14368652	4,79E-24
219919_s_at	SSH3	-1,14374531	8,21E-28

211657_at	CEACAM6	-1,1441458	3,49E-11
214609_at	PHOX2A	-1,14468621	2,83E-24
212937_s_at	COL6A1	-1,14693209	4,28E-22
221901_at	KIAA1644	-1,14699431	3,57E-25
220209_at	PYY2	-1,14738429	2,54E-23
220819_at	FRMD1	-1,14829757	1,17E-24
212148_at	PBX1	-1,14845695	1,15E-08
207311_at	DOC2B	-1,1491859	5,16E-24
203439_s_at	STC2	-1,14945662	5,39E-25
216310_at	TAOK1	-1,15044777	3,49E-21
214443_at	PVR	-1,15192982	9,05E-24
210532_s_at	C14orf2	-1,1529143	1,01E-15
210242_x_at	ST20	-1,15364142	4,36E-25
215389_s_at	TNNT2	-1,15387267	1,86E-24
216220_s_at	ADORA1	-1,15454869	4,26E-24
205360_at		-1,15547297	1,21E-23
219314_s_at	ZNF219	-1,15620317	2,21E-23
203661_s_at	TMOD1	-1,15693689	4,68E-11
208495_at	TLX3	-1,15908233	3,75E-25
211372_s_at	IL1R2	-1,15943123	4,74E-09
222378_at		-1,16112636	1,92E-15
205557_at	BPI	-1,16183731	4,32E-10
200781_s_at	RPS15A	-1,16207272	7,52E-12
215930_s_at	CTAGE5	-1,16233082	1,13E-22
221672_s_at	TRAPPC9	-1,16499246	3,32E-23
222080_s_at	SIRT5	-1,16580201	8,76E-25
221099_at		-1,16716532	4,06E-25
205389_s_at	ANK1	-1,16754501	2,03E-10
222104_x_at	GTF2H3	-1,16943144	2,53E-19
209235_at	CLCN7	-1,17101028	4,35E-21
220653_at	ZIM2	-1,17433615	5,68E-24
211821_x_at	GYPA	-1,17521964	1,71E-08
205390_s_at	ANK1	-1,17546222	1,02E-14
220024_s_at	PRX	-1,1767155	1,06E-25
206657_s_at	MYOD1	-1,17675936	6,53E-22
207193_at	AGRP	-1,18039765	3,55E-24
201249_at	SLC2A1	-1,18054004	5,86E-17
202129_s_at	RIOK3	-1,18345239	7,47E-12
205754_at	F2	-1,18647026	1,40E-24
200727_s_at	ACTR2	-1,18680227	6,73E-19
219046_s_at	PKNOX2	-1,18708241	1,43E-22
209996_x_at	PCM1	-1,18883985	1,20E-23
207567_at	SLC13A2	-1,19002825	2,21E-23
207281_x_at	VCX2	-1,19123895	1,04E-23
202701_at	BMP1	-1,19423464	2,26E-24
207041_at	MASP2	-1,19515426	2,10E-23
215420_at	IHH	-1,19534763	1,12E-22
217009_at	PGK2	-1,19569627	4,35E-25
212768_s_at	OLFM4	-1,19587913	0,000127018
215492_x_at	PTCRA	-1,20125896	2,04E-21

202219_at	SLC6A8	-1,20832927	2,84E-11
221870_at	EHD2	-1,2096554	9,87E-23
221477_s_at	SOD2	-1,21080863	2,56E-19
214242_at	MAN1A2	-1,21115667	2,49E-25
200741_s_at	RPS27	-1,21224776	1,65E-13
213148_at	C2orf72	-1,21234443	5,22E-26
200834_s_at	RPS21	-1,214122	1,88E-12
210884_s_at	SPAG11A	-1,21968333	2,49E-23
220561_at	IGF2AS	-1,22194755	4,52E-23
221205_at		-1,22500178	5,11E-17
204600_at	EPHB3	-1,22583099	1,96E-23
205650_s_at	FGA	-1,22835483	1,56E-22
204750_s_at	DSC2	-1,22967853	4,11E-17
216400_at	GBA /// GBAP1	-1,23009064	3,55E-24
221226_s_at	ACCN4	-1,23332137	5,38E-23
221979_at	LOC100129250	-1,23364299	3,47E-27
205863_at	S100A12	-1,23741898	3,99E-09
216500_at		-1,23798119	2,42E-24
208607_s_at	SAA1 /// SAA2	-1,23944008	2,67E-24
218401_s_at	ZNF281	-1,23947316	2,43E-22
215280_s_at	PPFIA3	-1,23956262	2,83E-25
211199_s_at	ICOSLG	-1,24154471	1,73E-23
201333_s_at	ARHGEF12	-1,24499257	2,74E-13
204611_s_at	PPP2R5B	-1,24634646	5,29E-22
210598_at		-1,24781475	4,37E-22
201655_s_at	HSPG2	-1,24909697	3,69E-24
215123_at	NPIPL3	-1,25508046	6,09E-15
211050_x_at	LOC100134822 /// LOC100288069	-1,25675042	3,68E-25
209273_s_at	ISCA1	-1,25770486	7,32E-12
214433_s_at	SELENBP1	-1,26074392	3,57E-10
204787_at	VSIG4	-1,26126837	3,69E-17
214575_s_at	AZU1	-1,26382505	9,43E-10
211546_x_at	SNCA	-1,26622219	2,21E-11
221368_at	NEU2	-1,26647257	4,06E-25
217799_x_at	UBE2H	-1,26745238	2,21E-20
214614_at	MNX1	-1,27745226	3,54E-27
207459_x_at	GYPB	-1,2808026	7,97E-11
216229_x_at	HCG2P7	-1,28218339	9,24E-20
209185_s_at	IRS2	-1,28242638	8,86E-16
221747_at	TNS1	-1,28322625	5,04E-11
213059_at	CREB3L1	-1,28613662	5,83E-24
217381_s_at	TRGV5	-1,29383197	6,41E-23
217274_x_at	MYL4	-1,29703964	2,47E-19
213446_s_at	IQGAP1	-1,29950131	8,96E-22
220863_at	MIP	-1,30301045	4,35E-25
206844_at	FBP2	-1,31086646	5,33E-24
203458_at	SPR	-1,31846298	2,51E-24
213846_at	COX7C	-1,32120348	1,67E-18
220811_at	PRG3	-1,32229646	4,79E-24
202425_x_at	PPP3CA	-1,32483694	1,70E-28

210655_s_at	FOXO3 /// FOXO3B	-1,33130765	1,09E-21
217071_s_at	MTHFR	-1,33181433	3,25E-24
215499_at	MAP2K3	-1,33193961	2,02E-23
203115_at	FECH	-1,33241301	4,58E-09
220712_at	C8orf60	-1,33487511	3,55E-23
214535_s_at	ADAMTS2	-1,33534495	7,05E-26
215150_at	YOD1	-1,33618995	1,61E-11
202210_x_at	GSK3A	-1,33662247	6,66E-25
206847_s_at	HOXA7	-1,34640485	1,38E-23
219672_at	AHSP	-1,34655712	2,12E-10
207499_x_at	UNC45A	-1,34818732	1,42E-22
218864_at	TNS1	-1,35113952	6,56E-12
201568_at	UQCRQ	-1,35175085	1,35E-13
219897_at	RNF122	-1,36128073	8,32E-26
209423_s_at	PHF20	-1,36174547	1,06E-25
215498_s_at	MAP2K3	-1,36503344	5,97E-23
214898_x_at	MUC3B	-1,36833863	2,55E-25
210177_at	TRIM15	-1,37102704	8,45E-24
206195_x_at	GH2	-1,37461727	1,35E-24
215687_x_at	PLCB1	-1,3827848	1,93E-23
220807_at	HBQ1	-1,38362814	1,87E-16
212531_at	LCN2	-1,39137437	1,12E-10
62987_r_at	CACNG4	-1,39365507	9,81E-24
222229_x_at	RPL26	-1,39660528	2,55E-17
209364_at	BAD	-1,40952007	1,31E-26
205040_at	ORM1	-1,41028754	2,25E-13
216082_at	NEU3	-1,41054538	1,31E-25
206676_at	CEACAM8	-1,41634329	2,10E-07
206578_at	NKX2-5	-1,41641877	2,02E-23
47571_at	ZNF236	-1,41904801	5,36E-22
204467_s_at	SNCA	-1,42446307	4,70E-13
202124_s_at	TRAK2	-1,42497796	2,09E-17
208695_s_at	RPL39	-1,43092242	1,33E-13
202018_s_at	LTF	-1,43116908	5,90E-09
213946_s_at	OBSL1	-1,43820824	8,24E-24
205592_at	SLC4A1	-1,43843237	2,08E-13
214407_x_at	GYPB	-1,4439607	5,15E-11
210172_at	SF1	-1,4533716	6,75E-15
221929_at		-1,45619285	8,39E-25
212472_at	MICAL2	-1,46863039	2,35E-22
206834_at	HBD	-1,46871245	4,36E-12
205033_s_at	DEFA1 /// DEFA1B /// DEFA3	-1,47058295	4,38E-12
202831_at	GPX2	-1,4722914	2,39E-24
216439_at	TNK2	-1,4787326	6,48E-25
220193_at	C1orf113	-1,47950669	1,29E-26
202203_s_at	AMFR	-1,48110296	3,20E-11
205041_s_at	ORM1 /// ORM2	-1,48422339	3,71E-09
217572_at		-1,48909437	2,83E-22
AFFX-M27830_M_at		-1,49110688	4,27E-23
217442_at	LOC100131825	-1,49229528	2,33E-25

208416_s_at	SPTB	-1,49697124	1,34E-20
202090_s_at	UQCR11	-1,49772222	2,40E-20
206698_at	XK	-1,51158291	2,30E-09
203116_s_at	FECH	-1,51732824	3,50E-12
202148_s_at	PYCR1	-1,51990881	1,96E-24
211696_x_at	HBB	-1,53053768	2,62E-15
206303_s_at	NUDT4	-1,5386816	8,22E-16
206123_at	LLGL1	-1,5646914	7,90E-23
216833_x_at	GYPB	-1,56597301	3,30E-15
215838_at	LILRA5	-1,56763494	1,68E-12
203154_s_at	PAK4	-1,5704984	7,30E-23
205653_at	CTSG	-1,5711923	2,72E-17
203911_at	RAP1GAP	-1,58302651	2,56E-09
203021_at	SLPI	-1,58829968	1,75E-17
205986_at	AATK	-1,59094135	1,79E-25
205021_s_at	FOXN3	-1,61988782	1,38E-23
216022_at		-1,62443839	1,39E-23
221748_s_at	TNS1	-1,63292706	2,78E-12
46947_at	GNL3L	-1,65245101	4,63E-22
209116_x_at	HBB	-1,6527746	4,29E-15
206450_at	DBH	-1,66697972	1,47E-24
212183_at	NUDT4	-1,68674196	1,89E-17
206416_at	ZNF205	-1,6931748	1,25E-24
204351_at	S100P	-1,70127657	3,96E-11
206177_s_at	ARG1	-1,70235498	4,66E-11
217232_x_at	HBB	-1,71742311	1,51E-16
201094_at	RPS29	-1,78147316	2,49E-19
209293_x_at	ID4	-1,79560887	3,47E-23
AFFX-M27830_5_at		-1,8728552	8,02E-17
205950_s_at	CA1	-1,99214688	5,64E-14
207269_at	DEFA4	-2,02771839	4,24E-15
AFFX-hum_alu_at		-2,0286944	6,58E-20
206871_at	ELANE	-2,11804117	1,31E-19
222084_s_at	SBF1	-2,19204799	3,58E-24
216671_x_at	MUC8	-2,67909889	1,12E-25
AFFX-HUMRGE/M10098_M_at		-2,99771565	5,51E-23
